# Insights into the *Staphylococcus aureus*-Host Interface: Global Changes in Host and Pathogen Gene Expression in a Rabbit Skin Infection Model

**DOI:** 10.1371/journal.pone.0117713

**Published:** 2015-02-26

**Authors:** Natalia Malachowa, Scott D. Kobayashi, Daniel E. Sturdevant, Dana P. Scott, Frank R. DeLeo

**Affiliations:** 1 Laboratory of Human Bacterial Pathogenesis, Rocky Mountain Laboratories, National Institute of Allergy and Infectious Diseases, National Institutes of Health, Hamilton, Montana, United States of America; 2 Research Technologies Branch, Rocky Mountain Laboratories, National Institute of Allergy and Infectious Diseases, National Institutes of Health, Hamilton, Montana, United States of America; 3 Veterinary Branch, Rocky Mountain Laboratories, National Institute of Allergy and Infectious Diseases, National Institutes of Health, Hamilton, Montana, United States of America; The Scripps Research Institute and Sorrento Therapeutics, Inc., UNITED STATES

## Abstract

*Staphylococcus aureus* is an important cause of human skin and soft tissue infections (SSTIs) globally. Notably, 80% of all SSTIs are caused by *S. aureus*, of which ∼63% are abscesses and/or cellulitis. Although progress has been made, our knowledge of the host and pathogen factors that contribute to the pathogenesis of SSTIs is incomplete. To provide a more comprehensive view of this process, we monitored changes in the *S. aureus* transcriptome and selected host proinflammatory molecules during abscess formation and resolution in a rabbit skin infection model. Within the first 24 h, *S. aureus* transcripts involved in DNA repair, metabolite transport, and metabolism were up-regulated, suggesting an increase in the machinery encoding molecules involved in replication and cell division. There was also increased expression of genes encoding virulence factors, namely secreted toxins and fibronectin and/or fibrinogen-binding proteins. Of the host genes tested, we found that transcripts encoding IL-8, IL1β, oncostatin M-like, CCR1, CXCR1 (IL8RA), CCL4 (MIP-1β) and CCL3 (MIP1α)-like proteins were among the most highly up-regulated transcripts during *S. aureus* abscess formation. Our findings provide additional insight into the pathogenesis of *S. aureus* SSTIs, including a temporal component of the host response. These results serve as a springboard for future studies directed to better understand how/why mild or moderate SSTIs progress to invasive disease.

## Introduction

Human skin is an essential first line defense against microbial pathogens and creates a continuously self-renewing interface between the host and its external environment. Skin is comprised of three primary layers: epidermis, dermis and hypodermis (subcutis), with epidermis being the most outer layer. In addition to providing a mechanical barrier to infection, skin also possesses a potent cellular defense mechanism to protect the host from invading pathogens. Skin immune cells consist of tissue-resident macrophages, dendritic cells, Langerhans cells, mast cells, T lymphocytes, and innate lymphoid cells (reviewed in [1]). Not to be overlooked, keratinocytes play an important role in host defense against skin infection. Since keratinocytes are primary building blocks of the skin, these cells are the first to encounter and respond to invading pathogens. Keratinocytes recognize pathogen-associated molecular patterns (PAMPs) present on the microbial surface through receptors such as Toll-like receptors (TLRs) [2,3] or nucleotide-binding oligomerization domain (NOD)-like receptors [4]. Recognition/binding of microbes by these receptors triggers secretion of pro-inflammatory cytokines and initiation of the innate immune response. The immune response is further shaped through receptor specificity; for example, TLR2 is a key receptor for recognition of Gram-positive bacteria such as *Staphylococcus aureus* [3,4].

*S*. *aureus* is a primary cause of bacterial skin and soft tissue infections (SSTIs) in the United States [5–7], and many of these infections are caused by strain USA300 [8]. The vast majority of human SSTIs present as skin abscesses and cellulitis [7]. In general, the success of *S*. *aureus* as a human pathogen is dependent on the ability to produce a variety of virulence factors and adapt to diverse host environments. The longstanding coexistence of *S*. *aureus* with the human host has enabled the pathogen to develop means to counteract the host immune system at virtually all stages of SSTI (reviewed in [9]), including the ability to produce high levels of secreted molecules that facilitate evasion of the innate immune response [10]. Among these molecules are staphylococcal cytolytic toxins, such as α- and γ-hemolysins, phenol-soluble modulins, and leukotoxins, which directly affect neutrophil function and can contribute to the skin infection [11–17].

Herein we investigated host-pathogen interactions in a rabbit skin and soft tissue model of infection caused by USA300, an epidemic community-associated methicillin-resistant *S*. *aureus* (CA-MRSA) strain. We monitored changes in gene expression of host inflammatory cytokines and receptors, and characterized the *S*. *aureus* transcriptome during experimental SSTI.

## Materials and Methods

### Ethics Statement

#### Animal Studies

Animal studies were approved by the Animal Care and Use Committee at Rocky Mountain Laboratories, National Institute of Allergy and Infectious Diseases (NIAID), and conformed to the guidelines of the National Institutes of Health (NIH). IACUC protocol numbers; RML #2011-23 and RML #2011-53. For shaving and subcutaneous inoculation of bacteria into rabbits, the animals were anesthetized with Acepromazine (5 mg/rabbit; all rabbits were approximately the same age and weight). At the end of the study, all animals were euthanized by i.v. administration of buthanasia (60 mg/kg) via the ear vein.

#### Human Subjects Research

Venous blood was obtained from healthy human volunteers in accordance with a protocol (01-I-N055) approved by the Institutional Review Board for Human Subjects, US National Institute of Allergy and Infectious Diseases, National Institutes of Health. Studies were conducted according to the policies provided in the Declaration of Helsinki. Each volunteer gave written informed consent prior to participation in the study.

### Bacteria Strains and Culture Conditions

*S*. *aureus* strain USA300 (LAC) and an isogenic *Δpvl/ΔlukGH* deletion strain (*Δpvl/ΔlukGH*) [18,19] were cultured in trypticase soy broth (TSB; Becton Dickinson, Franklin Lakes, NJ) in a rotary shaker at 225 rpm and 37°C. Bacteria from overnight (18 h) culture were diluted 1:200 with fresh media and grown to mid-log (OD_600_ = 0.75) or early stationary phase of growth (OD_600_ = 2.0). To prepare the inoculum, bacteria at early stationary phase of growth were collected by centrifugation, washed twice with Dulbecco’s phosphate buffered saline (DPBS; Sigma-Aldrich Company, St. Louis, MO) and suspended to 5 × 10^9^ CFUs/ml in DPBS.

### Rabbit Model of Skin and Soft Tissue Infection

The rabbit model of skin and soft tissue infection was performed as described previously [16]. Briefly, 5 groups (each group corresponds to an abscess excision day) of outbred immunocompetent female New Zealand White rabbits (strain Crlc:KBL(NZW); Western Oregon Rabbit Company, Philomath, OR) received 5 × 10^8^ CFUs of USA300 strain LAC in 0.1 ml sterile phosphate-buffered saline by subcutaneous injection in both right and left flanks. One rabbit from each group additionally received 0.1 ml of sterile saline into the right lower flank as a control. Two animals were used for each time point (2–4 abscesses/time point). Animals were allowed food and water *ad libitum* and were monitored daily. Abscesses and surrounding tissues were excised on day 1, 3, 6, 10 and 14-post infection following humane euthanasia of rabbits according to a protocol approved by the Institutional Animal Care and Use Committee (see *Animal study* section). Skin samples with underlying soft tissue were excised from two healthy rabbits and used as a baseline control in this study. Excised material was immediately flash-frozen in liquid nitrogen and stored at -80°C.

### RNA Isolation

Frozen abscess tissue was pulverized using a hammer and 3 × 50 mg of the abscess material was used to purify RNA. Samples were treated with QIAzol Lysis Reagent (Qiagen, Valencia, CA), and subsequently homogenized with a FastPrep-24 sample preparation system (MP Biomedicals, Santa Ana, CA) and RNA was extracted using an RNeasy Microarray Tissue kit (Qiagen) according to the manufacturer’s protocol. DNA was removed by treatment with TURBO DNase (Ambion/Life Technologies, Grand Island, NY) for 45 min at 37°C. RNA quality and concentration were assessed by use of a 2100 Bioanalyzer (Agilent Technologies, Inc., Santa Clara, CA).

### Microarray Experiments

Total RNA obtained from homogenized abscess samples was treated with MICROB*Enrich* (Ambion/Life Technologies) to increase the concentration of prokaryotic RNA. Approximately 500 ng of prokaryotic RNA was amplified, biotin-dUTP-labeled (Biotin-16-dUTP; Roche Applied Science, Indianapolis, IN) and fragmented using a MessageAmp II-Bacteria kit (Ambion/Life Technologies) according to the manufacturer’s protocol. Bacterial RNA was hybridized to a custom Affymetrix GeneChip (RMLchip7, Gene Expression Omnibus (GEO) platform GPL8069, http://www.ncbi.nlm.nih.gov/projects/geo/) that contains all open reading frames of the USA300 genome (100% coverage, 2560/2560 total ORFs). Hybridization, fluidics and scanning were performed as described [15], according to standard Affymetrix protocols (http://www.affymetrix.com). Command Console (CC v3.1, http://www.Affymetrix.com) software was used to convert the image files to cell intensity data (cel files). All “.cel” files, representing individual samples, were normalized by using the trimmed mean scaling method within expression console (EC v1.2, http://www.Affymetrix.com) to produce the analyzed “.cel” files (chp files) along with the report files. An ANOVA was performed within Partek (Partek, Inc. St. Louis, Mo., v6.6 6.12.0308) to obtain multiple test-corrected *P*-values using the false discovery rate method (FDR; Benjamini & Hochberg, 1995). Significance at the 0.05 confidence level and a two-fold expression difference were used to filter the probe-set list. *S*. *aureus* transcriptome analysis was performed on abscess samples excised on days 1, 3, 6, 10 and 14. The quality of bacterial RNA obtained from samples beyond day 1 was not sufficient to acquire reliable microarray data and only data from abscesses isolated 24 h post infection were used in this study. Microarray data are MIAME compliant and have been posted online at http://www.ncbi.nlm.nih.gov/projects/geo/ under series number GSE61669.

### TaqMan Real-Time Reverse Transcriptase PCR

Transcript levels of selected *S*. *aureus* genes were evaluated by TaqMan real-time RT-PCR using an ABI 7500 thermocycler (Applied Biosystems). The relative quantification of *S*. *aureus* mRNA was determined by comparing changes in target transcripts levels relative to *gyrB* (housekeeping gene), in accordance with the manufacturer’s protocol (Applied Biosystems Relative Quantification Manual). Data are expressed as the mean fold-change in transcript relative to the inoculum (control sample).

### Rabbit Inflammatory Response

cDNA was synthesized from total RNA (0.5 μg) with the RT^2^ First Strand kit (Qiagen). Changes in the expression level of genes encoding host inflammatory cytokines and receptors were detected using RT^2^ Profiler PCR Array Rabbit Inflammatory Cytokines and Receptors platform in combination with RT^2^ SybrGreen ROX qPCR Mastermix (Qiagen) in accordance with the manufacturer’s protocol. Samples were amplified using a 7500 Real Time PCR System (Applied Biosystems) and C_T_ values were determined with software version 2.0.6. Three test samples were used for abscesses obtained on days 1 through 10 post-infection, whereas only 2 test samples were available for day 14. Skin samples from two untreated healthy rabbits were used as a control. RT^2^ profiler PCR Array Data Analysis was performed using a downloaded template (Qiagen; www.sabiosciences.com/pcrarraydataanslysis.php#Excel) and appended to accommodate the controls, pertinent gene table, and our sample size for fold change (2^-ΔΔCt^) and *P*-value. *P*-value was calculated using two-tailed equal variance t-test. Data were partially analyzed through the use of QIAGEN’s Ingenuity Pathway Analysis (IPA, QIAGEN Redwood City, www.qiagen.com/ingenuity). To simplify data presentation, the LOC (or GeneID) number of each rabbit gene was substituted with the name of the human ortholog in the figures.

### *S*. *Aureus*-Induced Expression of Inflammatory Cytokines in Rabbit and Human Whole Blood

*S*. *aureus* were cultured to mid-exponential phase of growth, collected by centrifugation, washed twice and suspended in DPBS. Bacteria at a final concentration of 10^7^ CFU/ml were mixed with rabbit or human heparinized blood and incubated for 2 h with constant rotation at 37°C. Heparinized blood was used as a control. At time points specified, blood was combined with 5 volumes of erythrocyte lysis buffer (Buffer EL, Qiagen) and RNA purification was performed using QIAshredder columns in conjunction with the RNeasy Microarray Tissue Mini Kit according to the manufacturer instructions (Qiagen). The concentration and quality of purified RNA were assessed using a 2100 Bioanalyzer (Agilent Technologies, Inc.). Cytokine gene expression was assessed by use of Human or Rabbit Inflammatory Cytokines and Receptors RT² Profiler PCR Arrays (Qiagen) as described above.

### RNA *In situ* Hybridization

Detection of cytokine mRNA was performed using the RNAscope FFPE assay (Advanced Cell Diagnostics Inc., Hayward, CA) as previously described [20] and in accordance with the manufacturer’s instructions (Advanced Cell Diagnostics Inc.). Briefly, excised lesions were fixed in 10% neutral-buffered formalin for at least 48 hours and samples were prepared as described previously [12]. Whole-tissue sections were deparaffinized, processed and subsequently hybridized with target-specific probes for selected cytokines mRNA using RNAscope VS FFPE reagent kit (BROWN) in conjunction with Ventana Discovery XT slide autostaining system (Ventana Medical Systems Inc., Tucson, AZ). VS Probe–Oc-*POLR2A* (*Oryctolagus cuniculus* DNA directed RNA polymerase II polypeptide A, *POLR2A*) mRNA and VS Probe–Oc-PPIB (*O*. *c*. cyclophilin B (*CYP5*), mRNA, were used as positive and negative controls, respectively. VS Probe-*dapB* (dihydrodipicolinate reductase) against *Bacillus subtilis* strain SMY was used as an additional negative control. Target probes against *IL17A* (VS Probe–Oc-IL17A), *IL17F* (VS Probe–Oc-IL17F), *IL6* (VS Probe–Oc-IL6), *IL8* (VS Probe–Oc-IL8), *TNF* (VS Probe–Oc-TNF) and *IL1B* (VS Probe–Oc-IL1B) rabbit specific cytokines and control probes were purchased from Advanced Cell Diagnostics Incorporated. All tissue samples were evaluated by a veterinary pathologist (D.P.S.). Images of tissue sections were captured using an Olympus model BX-51 microscope (Olympus, Center Valley, PA), and brightness and contrast of images was adjusted in Adobe Photoshop CS5.1 (Adobe Systems Inc., San Jose, CA). We attempted to quantitate samples by morphometric analysis; however, the small number of positive cells was insufficient to permit statistical analysis.

## Results

### Changes in the *S*. *aureus* Transcriptome during Rabbit Skin Infection

**Fig 1:**
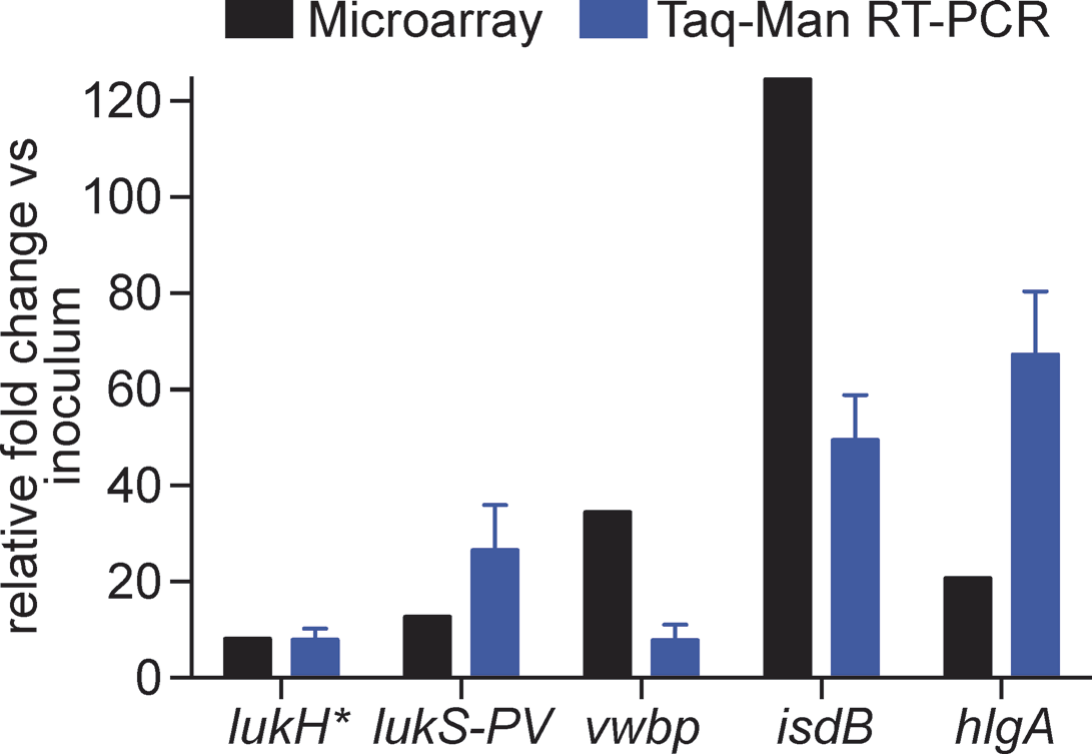
TaqMan real-time PCR verification of changes in *S*. *aureus* transcript levels. Results are expressed as an average relative fold change in bacterial transcripts from three abscesses excised 24 h post infection and compared to the level of bacterial transcript expression in the inoculum. The relative quantification of *S*. *aureus* transcript was determined by the change in expression of target transcripts relative to that of *gyrB*. *Also known as *lukA*.

To increase our understanding of host-pathogen interactions during the progression of *S*. *aureus* SSTIs, we evaluated global changes in pathogen gene expression and changes in expression of genes encoding host inflammatory cytokines and receptors in a rabbit model of *S*. *aureus* skin infection. Subcutaneous inoculation of *S*. *aureus* strain USA300 induced formation of typical skin abscesses, characterized by an influx of polymorphonuclear leukocytes (PMNs or neutrophils) and development of a fibrous pseudocapsule during abscess maturation (described in detail in our previous studies [14,16]). Abscess samples were obtained on Days 1, 3, 6, 10 and 14 post infection, and RNA was used for microarray and real time PCR analysis. Microarray analysis of the USA300 samples obtained 24 h post infection indicated that bacteria undergo a major metabolic switch, perhaps as an attempt to survive in a new environment (S1 Table). Significant differentiation of the bacterial transcriptomes from the inoculum and 24 h abscess samples was also reflected by principal component analysis (PCA) (S1 Fig.). For example, numerous genes involved in transport of metabolites and nutrients, such as ABC transporters, multidrug resistance efflux pumps, oligopeptide transporters, as well as genes involved in DNA repair, were up-regulated and there were changes in expression of genes encoding molecules involved in major metabolic pathways (S1 Table). In addition, transcript levels of *S*. *aureus* virulence factors including those encoding leukocidins, proteases and fibronectin or fibrinogen-binding proteins was increased (Table 1 and Fig. 1). The microarray results were confirmed by TaqMan real-time reverse-transcriptase PCR analysis of selected *S*. *aureus* genes (*lukH*, *lukS-PV*, *vwbp*, *isdB* and *hlgA*) (Fig. 1). Comparison of *S*. *aureus* transcript levels in rabbit skin in vivo and broth culture in vitro does not provide information about the function or relative contribution of these molecules to disease in the rabbit. Rather, the data identify molecules that potentially contribute to disease in vivo and that can be investigated in future studies.

**Table 1:** Changes in USA300 transcript levels of selected virulence factor-encoding genes in rabbit skin abscesses at 24 h post infection.

Gene name/ ORF	Description	Fold change Day1/inoculum
***isdB***	Iron transport associated domain	124.58
***hlgA***	Hemolysin γ, S subunit	20.8
***hlgC***	Hemolysin γ, S subunit	27.44
***hlgB***	Hemolysin γ, F subunit	76.31
***sbi***	IgG-binding protein SBI	24.12
***lukH***	Leukocidin GH, S subunit	8.19
***lukG***	Leukocidin GH, F subunit	5.24
***lukS-PV***	PVL, S subunit	12.74
***lukF-PV***	PVL, F subunit	6.64
**SAUSA300_1061**	Exotoxin (Exotoxin 3)	35.5
**SAUSA300_0370**	Exotoxin (Enterotoxin)^a^	9.3
***splB***	Serine protease	17.6
***splF***	Serine protease	4.4
***SAUSA300_0773***	Putative staphylocoagulase (vWbp)^a^	34.52
***empbp***	Extracellular matrix binding protein	11.71
***efb***	Fibrinogen-binding protein precursor	5.31
***sdrE***	Fibronectin-binding protein	12.04
***cap1A***	Chain length regulator (capsular polysaccharide biosynthesis)	124.1
***clfA***	Clumping factor A	-6.52
***SAUSA300_1052***	Fibrinogen-binding protein (Extracellular complement-binding protein/Ecb)^a^	5.86

Microarray results are presented as the mean fold-change from 3 abscesses. Only transcripts with significant fold-change (*P* ≤ 0.05 and more than 2-fold change in transcriptome) are included.

^a^Determined by BLASTProtein or BLASTGenomic available at http://www.ncbi.nlm.nih.gov.

### Rabbit Immune Response During USA300 SSTI

**Fig 2:**
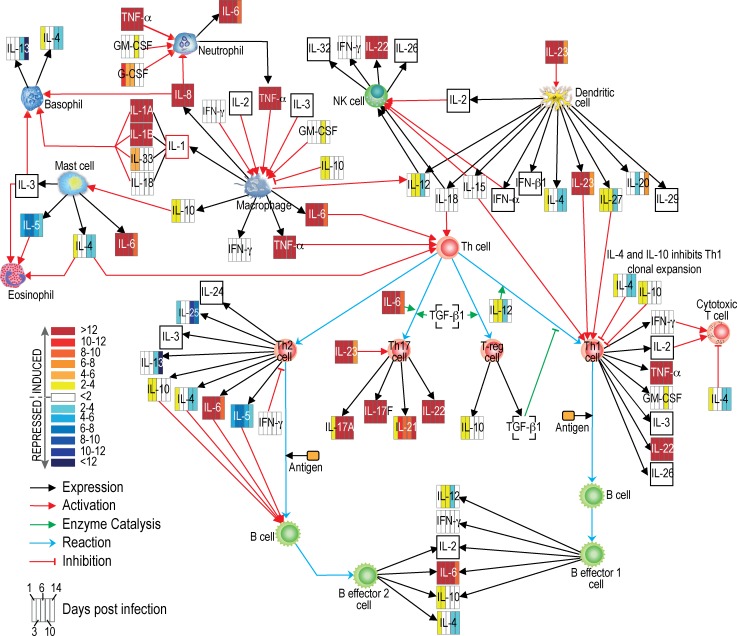
Changes in inflammatory molecule transcript levels during formation and resolution of *S*. *aureus* skin abscess. Gene expression data were obtained using RT^2^ Profiler PCR Array Rabbit Inflammatory Cytokines and Receptors platform. The networks/functional analyses were generated through the use of QIAGEN’s Ingenuity Pathway Analysis (IPA, QIAGEN Redwood City, www.qiagen.com/ingenuity). The heat map represents changes in transcript levels of molecules on days 1, 3, 6, 10 and 14 of a 14-day course of infection (compared to uninfected skin), and was created using Adobe Illustrator CS5.1 software. Unfilled squares; transcript levels were not available. For simplicity, the encoded proteins (rather than transcripts) and potential pathways are shown. The interactions and functions shown here are hypothetical based upon the known (published) general function of each molecule. Further studies of *S*. *aureus*-host interaction are necessary to verify these pathways and protein function during infection.

We employed a PCR array approach to evaluate the host immune response triggered by subcutaneous injection of *S*. *aureus* in a rabbit SSTI model. Among a panel of 84 genes encoding rabbit pro-inflammatory cytokines and receptors, there were 11 rabbit genes down-regulated and 52 genes up-regulated 24 h after USA300 infection relative to uninfected skin control. At times early following infection, multiple transcripts encoding proinflammatory molecules were increased, including *IL8* (8196-fold), *IL1B* (1559-fold), *oncostatin M* (*OSM*, 799-fold), *CCR1* (588-fold), *CXCR1* (625-fold), *CXCR2* (596-fold), *CCL4* (514-fold), and *CCL3* (658-fold) (S2 Table). The expression level of some proinflammatory molecules (e.g., *CCR5*, *CXCR4*, *IL1A*, *IL1R1*, *IL8*, and *TNF*) peaked on day 3 or 6 following infection. We observed that the pronounced early up-regulation of transcripts encoding many pro-inflammatory cytokines and receptors subsided by day 14 (e.g., *IL1*, *IL8*, *CXCR1*, and *CXCR2*, decreased 10-fold by day 14, and *IL6* decreased ∼25-fold by that timepoint; Fig. 2, S2 Table). Moreover there is a group of molecules, including *LTA*, *LTB*, *IL21*, *IFNAR2*, *CXCR3*, *IL17F* and *CD40LG*, whose expression increased over the course of USA300 infection.

### Host Immune Response to LukGH and Panton-Valentine Leukocidin (PVL)

Genes encoding PVL and LukGH were among the virulence factor genes up-regulated during the first 24 h post infection. To determine the relative contribution of these toxins to the host immune response, we employed RNA *in situ* hybridization approach to compare expression of genes encoding IL-17A, IL-17F, IL-6, TNFα, IL-1β and IL-8 in inflammatory lesions following subcutaneous injection of USA300 wild type or isogenic *Δpvl/ΔlukGH* mutant strains. Surprisingly, there was virtually no difference in number, type or location of the cells testing positive for cytokine mRNA (Table 2). Next, we evaluated the ability of purified LukGH or PVL to promote expression of the selected cytokines. The immune response elicited by purified leukotoxin was notably weaker than that caused by *S*. *aureus* (Table 2, S2 Fig.), albeit it was greater than that in uninfected PBS control tissues (data not shown). One possible factor contributing to the noted difference in response (at the level of transcript) between purified toxin and intact *S*. *aureus* is that the distribution and type of cells expressing cytokine mRNA was different between the samples tested. Cytokine mRNAs were detected throughout inflammatory lesions caused by purified toxins, whereas cytokine signal was detected primarily at the margin of *S*. *aureus* abscesses (Table 2). Increased expression of mRNA encoding IL-17A and IL-17F was detected predominantly within macrophages in areas of dermal inflammation caused by intradermal injection of purified toxins or at the deep border of abscesses in those animals inoculated with either USA300 strain. Similar findings were noted for *IL6*; however, the morphology of *IL6*-positive cells suggests these cells were macrophages, lymphocytes, and endothelial cells (Table 2). *TNF* was also present in small numbers of macrophages and lymphocytes within areas of inflammation or at the margins of abscesses (Table 2). *IL1B* and *IL8* transcripts were detected in a larger number of cells than the other cytokines, and they were primarily expressed by macrophages and neutrophils in areas of dermal inflammation in rabbits injected with LukGH or PVL (Table 2, S2 Fig.). In the subcutaneous lesions caused by USA300 wild type or *Δpvl/ΔlukGH* strains, the levels of *IL1B* and *IL8* were increased in numerous macrophages and neutrophils at and within the margins of abscesses. In addition, *IL8* was found in endothelial cells of vessels adjacent to abscesses (Table 2).

**Table 2:** Cytokine profiling *in vivo*.

Condition tested	Transcript					
	IL17A	IL17F	IL6	TNF	IL1B	IL8
**Strain USA300 (Day 6 p.i.)**	few macrophages at deep abscess margin	few macrophages at deep abscess margin	few dermal mononuclear and endothelial cells around the abscess	few mononuclear cells at abscess margin	many macrophages and PMNs at abscess margin	many macrophages, PMNs and endothelial cells
**Strain USA300*Δpvl/ΔlukGH* (Day 6 p.i.)**	few macrophages at deep abscess margin	few macrophages at deep abscess margin	few dermal mononuclear and endothelial cells around the abscess	few mononuclear cells at abscess margin	many macrophages and PMNs at abscess margin	many macrophages, PMNs and endothelial cells
**Purified LukGH at 24 h** ^**a**^	rare dermal macrophages in areas of inflammation	few dermal mononuclear cells in areas of inflammation	few dermal mononuclear and endothelial cells in areas of inflammation	few dermal mononuclear cells in areas of inflammation	many mononuclear cells and PMNs in dermis	few mononuclear cells and PMNs in dermis
**Purified PVL at 24 h** ^**a**^	rare dermal macrophages in areas of inflammation	few dermal mononuclear cells in areas of inflammation	few dermal mononuclear and endothelial cells in areas of inflammation	rare dermal macrophages in areas of inflammation	few mononuclear cells and PMNs in dermis	rare mononuclear cells and PMNs in dermis

*In situ* analysis of host gene expression at the site of inflammatory lesion caused by intradermal injection of 1000 ng of purified LukGH or PVL or by subcutaneous injection of 5×10^8^ CFU of USA300 wild type or *Δpvl/ΔlukGH* strains. Inflammation lesions with surrounding tissue were excised on day 1 after protein injection and day 6 after bacteria injection, which corresponded with the maximum size of the lesion.

^a^Proteins were purified from *S*. *aureus* USA300 culture and 1000 ng was administered by intradermal injection.

### Transcriptional Response of Rabbit and Human Blood Cells to *S*. *aureus*

**Fig 3:**
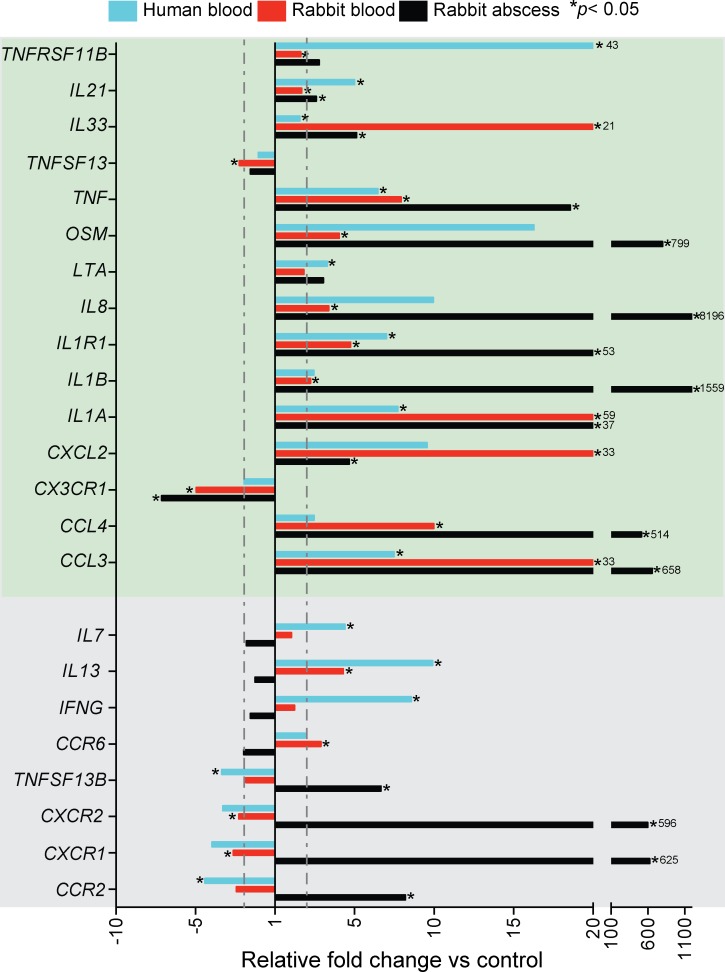
Real-time PCR analyses of the host inflammatory response to *S*. *aureus*. Transcript levels in 24 h rabbit abscess samples are presented as fold change relative to those in healthy skin. Results are the average fold-change of 3 abscesses. Changes in human and rabbit blood transcript levels following culture with *S*. *aureus* (10^7^ CFU/ml) are expressed as average fold change relative to transcript levels in heparinized blood alone (no bacteria) (S2 and S3 Tables). All blood samples were incubated for 2 h at 37°C and results are average of three blood donors. Relative transcript levels of molecules within the green box increased or decreased coordinately in all conditions tested. Transcript levels of the molecules in the grey box are discordant between blood and abscess tissue. The dotted lines indicate a 2-fold change in expression compared to control samples. *P<0.05 versus control samples using a two-tailed equal variance t-test.

To better understand the contribution of leukocytes to the production of inflammatory mediators in infected tissues, and to determine whether rabbit and human leukocytes have similar inflammatory responses to *S*. *aureus*, we measured changes in transcripts encoding selected inflammatory mediators in rabbit or human whole blood after incubation with *S*. *aureus* (S3 Table). First, we compared mRNA changes in rabbit and human blood after incubation with *S*. *aureus in vitro* to those in rabbit skin tissue 24 h after inoculation with the pathogen (Fig. 3). Fifteen of the 51 genes tested met criteria for being differentially expressed (i.e., at least 2-fold change compared to control and increased or decreased significantly in at least one of the conditions tested), and were similarly up- or down-regulated following infection with- or exposure to *S*. *aureus* under all conditions (Fig. 3, green box). Although many of the differentially expressed genes tested changed similarly in rabbit and human cells, or in blood leukocytes and abscess tissues, the relative expression of 4 cytokine and 4 receptor genes was varied between blood and abscess samples (S3 Table, Fig. 3, grey box).

## Discussion

*S*. *aureus* strain USA300 is a leading cause of human SSTIs, and these infections frequently manifest as abscesses. To better understand the dynamics of host-pathogen interaction during *S*. *aureus* SSTIs, we queried the USA300 and host transcriptomes in a rabbit *S*. *aureus* skin infection (abscess) model. Our data revealed that 24 h after s.c. infection with *S*. *aureus*, there were significant changes in expression of genes encoding molecules involved in metabolism and transport. It is not uncommon for bacteria to undergo a metabolic shift upon entering the host, where essential nutrients are often limited [15,21,22]. A recent study by Date et al. reported a similar trend in the expression of USA300 genes encoding transport and metabolism molecules recovered from human abscess material and mouse kidney abscesses [23]. In addition, it has been previously shown that transcripts encoding secreted virulence factors such as proteases or cytolytic toxins are up-regulated during infection [15,23–26]. Increased levels of leukotoxin transcripts during the first 24 h of infection suggests that they play a role in abscess formation. Our *in situ* analysis reinforced the notion that purified LukGH and PVL triggers a profound inflammatory response, as evidenced by increased expression of mRNAs encoding IL-8, IL-6, IL-1β, TNFα, IL-17A and IL-17F in a rabbit abscess model—a finding consistent with previous *in vivo* and *in vitro* studies [14,27]. Nevertheless, the rabbit aggregate proinflammatory response (based on the relative change in the expression level of selected cytokines) to USA300 and USA300*Δpvl/ΔlukGH* was nearly identical, suggesting that the ability of *S*. *aureus* to survive and cause disease is influenced by a well-orchestrated network of virulence factors rather than one or two virulence molecules.

**Fig 4:**
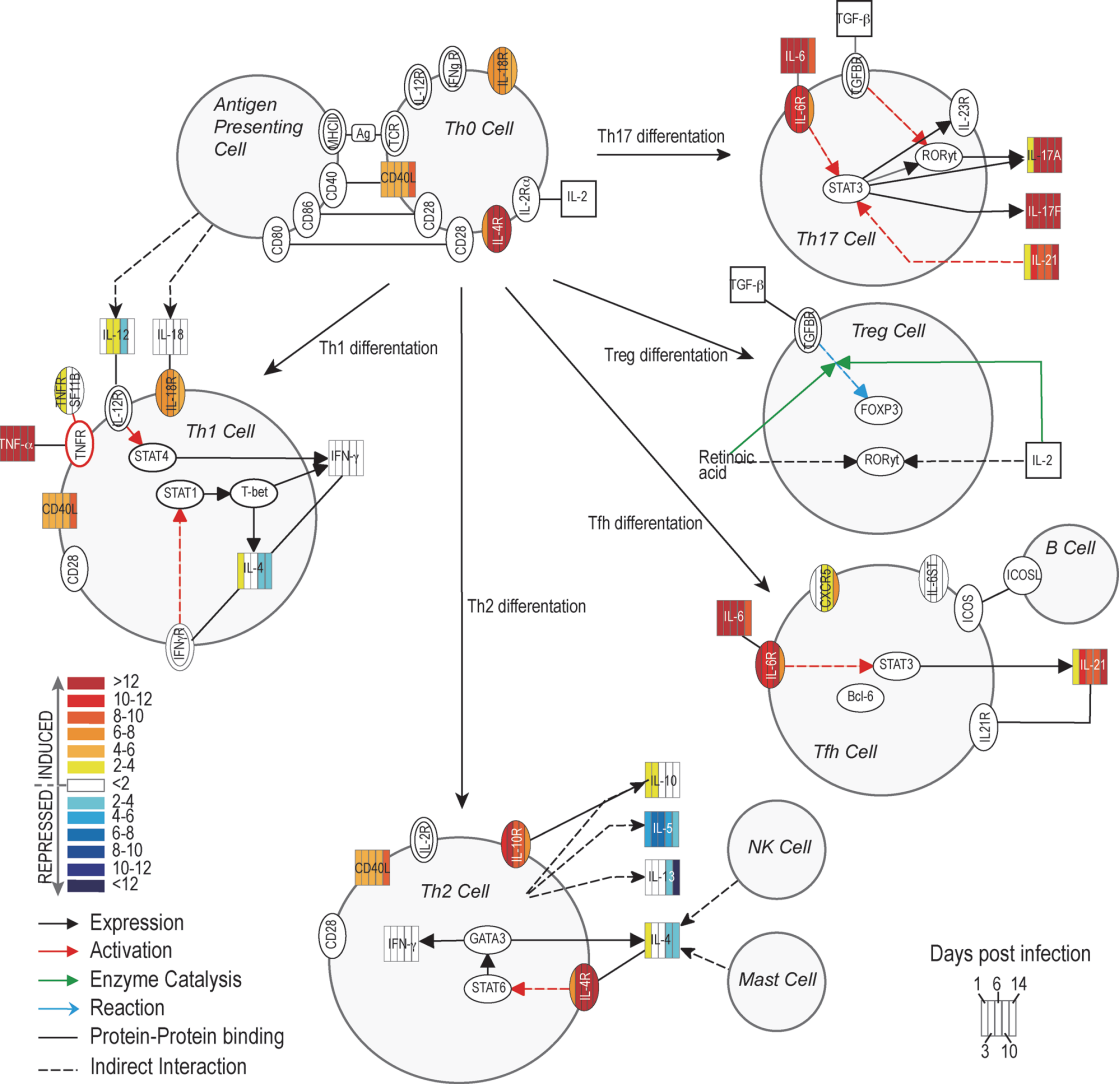
*S*. *aureus* skin infection causes changes in transcripts encoding molecules involved in Th lymphocyte differentiation. The networks/functional analyses were generated through the use of QIAGEN’s Ingenuity Pathway Analysis (IPA, QIAGEN Redwood City, www.qiagen.com/ingenuity). The heat map representing changes in transcript levels of molecules on day 1, 3, 6, 10 and 14 during 14-day course of infection, was created using Adobe Illustrator CS5.1 software. Empty symbols indicate molecules not evaluated by the RT² Profiler PCR Array.

Gram-positive bacteria, particularly *S*. *aureus*, are recognized in the skin by keratinocytes via the TLR2 receptor [3]. Subsequently, pro-inflammatory cytokines such as IL-1β are released and these molecules promote an influx of neutrophils to the site of infection and play a distinct role in skin abscess formation [28]. The initial up-regulation of pro-inflammatory cytokines corresponds with up-regulation of factors involved in expression of adhesion molecules on endothelial cells, which subsequently promote rolling and extravasation of leukocytes and their migration to the site of infection. These factors include OSM, IL-4 (gene up-regulated on day 1 post infection) and MIF (macrophage migration inhibitory factor) that induces P- and E-selectin and vascular cell adhesion molecule 1 (VCAM-1) [29–34]. Transcripts encoding a number of potent pro-inflammatory cytokines, namely IL-8, macrophage inflammatory protein 1α (MIP-1α or CCL3), and 1β (MIP-1β or CCL4), were highly expressed in abscesses on day 6, which correlates with the maximum size of abscesses as reported previously [16]. MIPs and their receptors also play a crucial role in T lymphocyte recruitment and differentiation [35,36]. The Ingenuity Pathway Analysis of changes in the host transcriptome revealed that many components of the Th17 pathway are up-regulated in *S*. *aureus* SSTIs (Fig. 4). We note that IL-12 and IFNγ (a Th1 cytokine response), and IL-10, IL-4, IL-13 or IL-5 (Th2 cytokine response) were either down-regulated during the latter phase of infection or remained unchanged. By contrast, cytokines involved in Th17 signal transduction, such as IL-17A, IL-17F or IL-21, were highly up-regulated after infection, and the Th17 pathway is known to contribute to inflammation and influx of neutrophils to the site of infection [37,38].

In this study, we provide a global view of the *S*. *aureus*-host interaction during SSTI. A comprehensive understanding of *S*. *aureus*-host interface is an important step toward development of new prophylactics and therapeutics directed to prevent or treat infections caused by MRSA.

## Supporting Information

S1 Fig Principal component analysis (PCA) of the *S*. *aureus* transcriptome in rabbit skin abscesses versus broth culture. Data are from abscess samples 24 h post-infection (red spheres) compared to *S*. *aureus* cultured to early stationary phase in TSB (blue spheres). Each sphere represents an individual sample, with the plotted location based upon the correlation of each sample relative to the others. The total amount of variation within the data set is 85% and each axis represents a different principal component.
(TIF)

S2 Fig *S*. *aureus*-induced expression of *IL1B* in the rabbit skin infection model. USA300 abscess, day 6 following infection (A, B, G and H). Inflammatory lesions caused by injection of purified LukGH (C, D, I and J) or PVL (E, F, K and L) 24 h post injection. A-F represent cross-sections of inflammatory lesions stained with H&E. (G-L) *in situ* hybridization analysis of *IL1B* mRNA in tissues from the same sample blocks as those shown in A, C, and E. Original magnification of images A, C, E, G, I and K is ×20. B, D, F, H, J and L are magnified ×1000 of the area depicted by the black rectangle. Black arrow, polymorphonuclear leukocyte; red arrow, endothelial cell; lymphocyte; blue arrow, macrophage.
(TIF)

S1 Table Changes in USA300 transcriptome during abscess formation in rabbit skin and soft tissue infection model at 24 h post infection. Microarray results are presented as the mean fold-change from three separate abscesses compared to the transcripts level in the inoculum.
(PDF)

S2 Table Changes in cytokine and receptor transcripts during formation and resolution of *S*. *aureus* skin abscesses in a rabbit infection model. Gene expression data was obtained using RT^2^ Profiler PCR Array Rabbit Inflammatory Cytokines and Receptors platform (QIAGEN). Cytokine transcripts that were changed significantly at any time point are in bold font.
(DOCX)

S3 Table Immune response toward *S*. *aureus* in human or rabbit whole blood. Human and rabbit blood results are expressed as an average relative fold change in rabbit or human transcripts expressed by cells in the whole blood co-cultured with 1 x 10^7^ CFU/ml of *S*. *aureus* compared to the level of transcripts expression in heparinized blood alone. Gene expression data was obtained using RT^2^ Profiler PCR Array Rabbit or Human Inflammatory Cytokines and Receptors platform (QIAGEN). Cytokines, which transcript levels changed above 2-fold and were statistically significant are highlighted in bold.
(DOCX)
