# Supplementary figures and images for: Insights into the Staphylococcus aureus-Host Interface: Global Changes in Host and Pathogen Gene Expression in a Rabbit Skin Infection Model
Source: PLoS One. 2015 Feb 26;10(2):e0117713. doi: 10.1371/journal.pone.0117713 (PMC4342162; doi:10.1371/journal.pone.0117713)

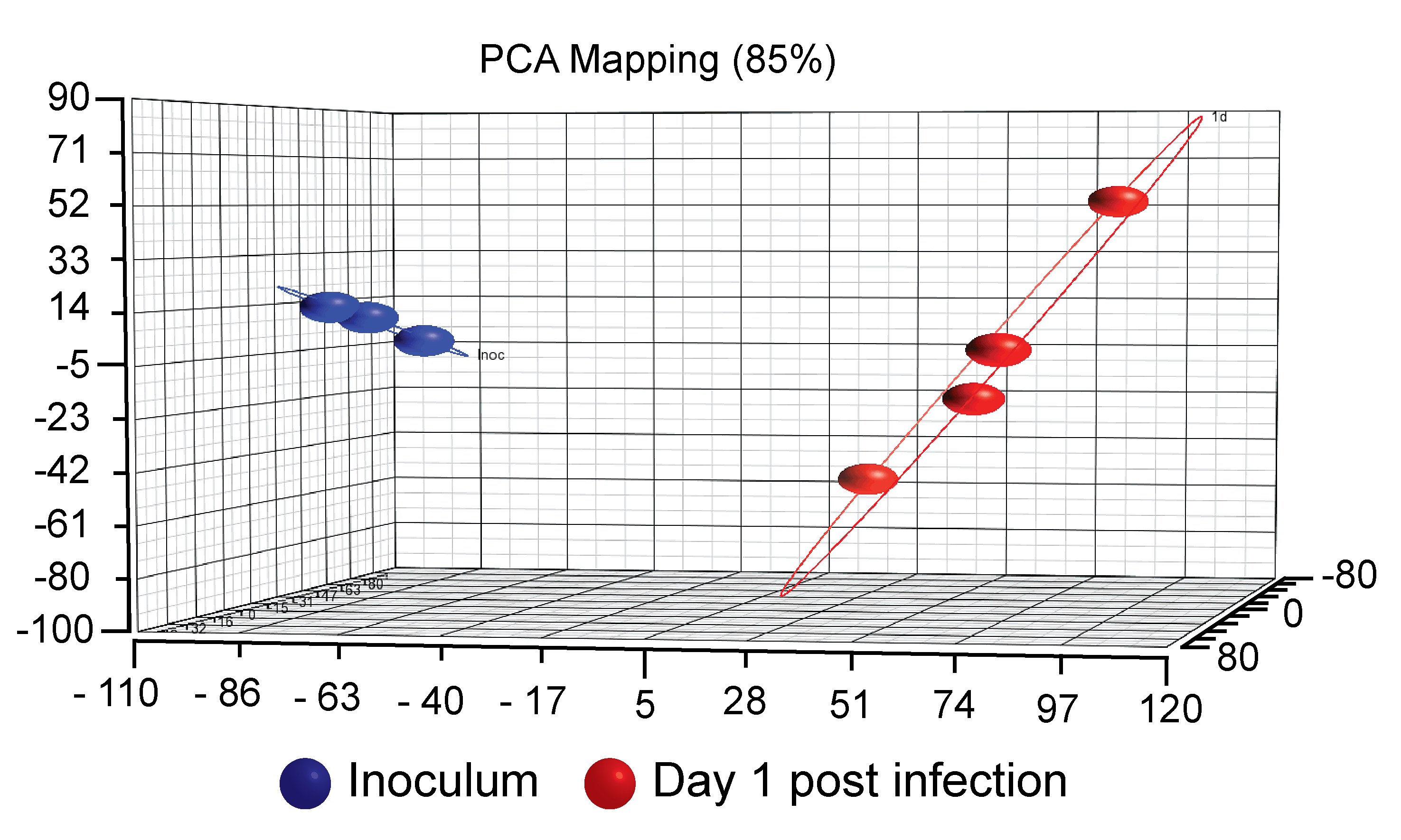

Supplement: S1 Fig — Data are from abscess samples 24 h post-infection (red spheres) compared to S. aureus cultured to early stationary phase in TSB (blue spheres). Each sphere represents an individual sample, with the plotted location based upon the correlation of each sample relative to the others. The total amount of variation within the data set is 85% and each axis represents a different principal component. (TIF) [file pone.0117713.s001.tif]

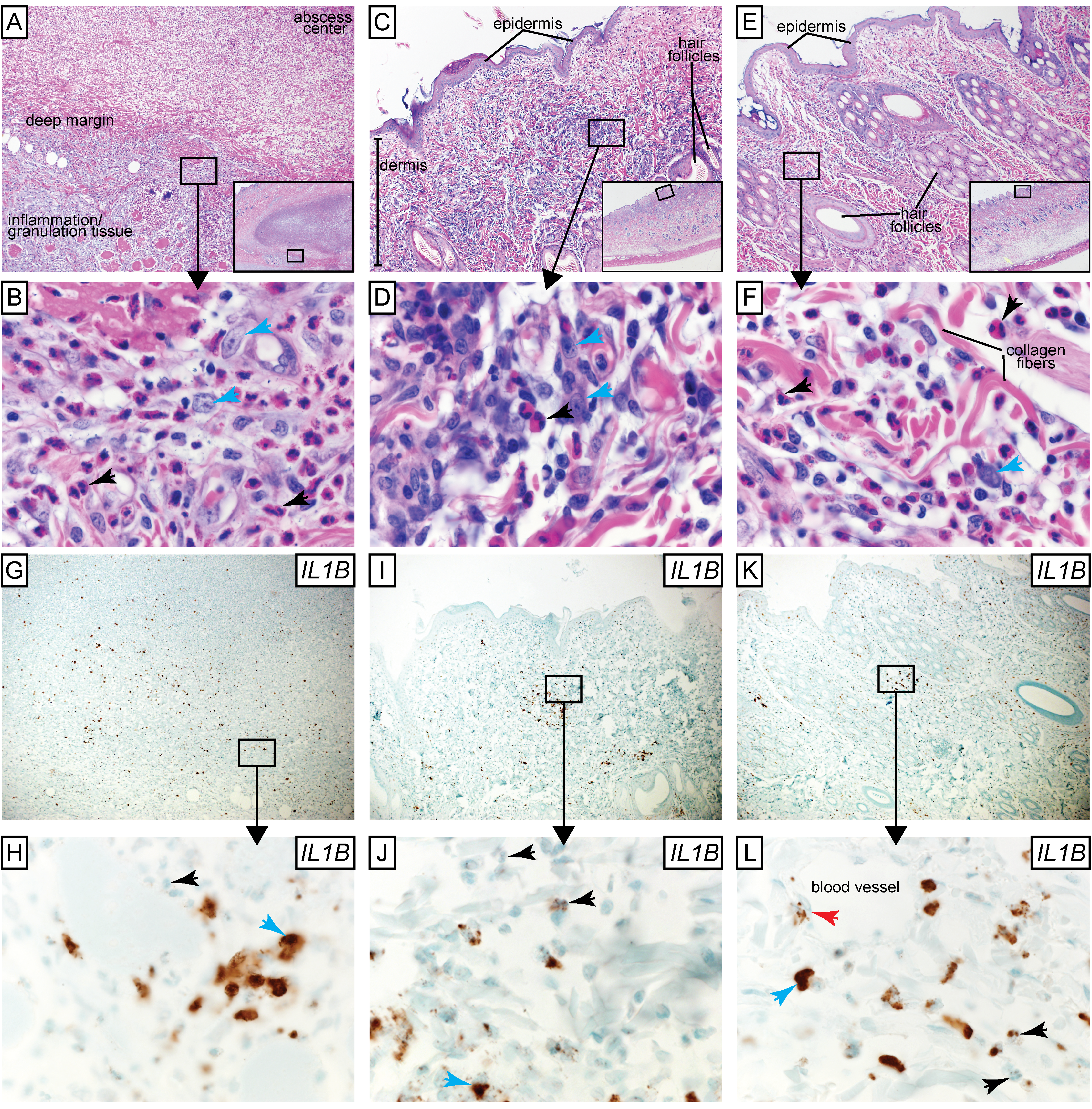

Supplement: S2 Fig — USA300 abscess, day 6 following infection (A, B, G and H). Inflammatory lesions caused by injection of purified LukGH (C, D, I and J) or PVL (E, F, K and L) 24 h post injection. A-F represent cross-sections of inflammatory lesions stained with H&E. (G-L) in situ hybridization analysis of IL1B mRNA in tissues from the same sample blocks as those shown in A, C, and E. Original magnification of images A, C, E, G, I and K is ×20. B, D, F, H, J and L are magnified ×1000 of the area depicted by the black rectangle. Black arrow, polymorphonuclear leukocyte; red arrow, endothelial cell; lymphocyte; blue arrow, macrophage. (TIF) [file pone.0117713.s002.tif]
