# Supplementary material for: Insights into the Staphylococcus aureus-Host Interface: Global Changes in Host and Pathogen Gene Expression in a Rabbit Skin Infection Model
Source: PLoS One. 2015 Feb 26;10(2):e0117713. doi: 10.1371/journal.pone.0117713 (PMC4342162; doi:10.1371/journal.pone.0117713)
Supplement: S1 Table — Microarray results are presented as the mean fold-change from three separate abscesses compared to the transcripts level in the inoculum. (PDF) [file pone.0117713.s003.pdf]

Table S1

probe set mapping	synonym	description	NCBI COG	p-value	day one / inoculum
SAUSA300_0003	-	Hypothetical cytosolic protein	Function unknown	4.74E-06	-3.93
SAUSA300_0004	recF	DNA replication and repair protein recF	Replication, recombination and repair	6.66E-04	-2.89
SAUSA300_0006	gyrA	DNA gyrase subunit A (EC 5.99.1.3)	Replication, recombination and repair	1.37E-04	-6.96
SAUSA300_0008	hutH	Histidine ammonia-lyase (EC 4.3.1.3)	Amino acid transport and metabolism	8.32E-07	11.92
SAUSA300_0009	serS	Seryl-tRNA synthetase (EC 6.1.1.11)	Translation	5.54E-04	-12.08
SAUSA300_0010	-	Branched-chain amino acid transport protein azlC	Amino acid transport and metabolism	2.03E-08	9.92
SAUSA300_0015	rplI	LSU ribosomal protein L9P	Translation	2.10E-03	-2.15
SAUSA300_0016	dnaB	Replicative DNA helicase (EC 3.6.1.-)	Replication, recombination and repair	1.87E-03	3.86
SAUSA300_0017	purA	Adenylosuccinate synthetase (EC 6.3.4.4)	Nucleotide transport and metabolism	4.02E-03	-3.01
SAUSA300_0021	-	Two-component sensor kinase yycG (EC 2.7.3.-)	Signal transduction mechanisms	2.02E-07	-3.95
SAUSA300_0022	-	hypothetical membrane protein yycH	Function unknown	5.85E-10	-3.26
SAUSA300_0032	mecA	MecA protein	---	1.38E-03	-3.34
SAUSA300_0034	-	Transposase	Replication, recombination and repair	6.07E-07	20.32
SAUSA300_0035	-	Type I restriction-modification system restriction subunit (	---	3.08E-06	-13.45
SAUSA300_0036	-	Hypothetical protein	Function unknown	2.10E-14	133.34
SAUSA300_0039	-	Hypothetical protein	Replication, recombination and repair	3.32E-13	15.45
SAUSA300_0040	-	Hypothetical protein	---	8.19E-10	14.47
SAUSA300_0044	-	Hydroxyacylglutathione hydrolase (EC 3.1.2.6)	General function prediction only	2.62E-14	10.75
SAUSA300_0045	-	Hypothetical protein	Defense mechanisms/Virulence	3.75E-03	6.27
SAUSA300_0046	-	Hypothetical protein	---	2.02E-08	-2.93
SAUSA300_0047	-	Type II restriction-modification system restriction subunit	Defense mechanisms/Virulence	3.80E-05	8.30
SAUSA300_0049	-	Hypothetical protein	---	3.55E-07	23.86
SAUSA300_0053	speG	Spermidine N1-acetyltransferase (EC 2.3.1.57)	Translation	3.88E-03	2.53
SAUSA300_0056	-	Hypothetical protein	Function unknown	1.85E-10	18.69
SAUSA300_0057	-	Pyridoxal phosphate-dependent enzymes	---	2.73E-07	11.12
SAUSA300_0059	-	Hypothetical protein	---	9.31E-06	56.51
SAUSA300_0062	arcB	Ornithine carbamoyltransferase (EC 2.1.3.3)	Amino acid transport and metabolism	2.22E-03	6.36
SAUSA300_0063	-	Transcription regulator, crp family	Signal transduction mechanisms	2.25E-06	5.26
SAUSA300_0064	arcD	Arginine/ornithine antiporter	Amino acid transport and metabolism	8.02E-06	5.16
SAUSA300_0067	-	Putative transcription factors	Signal transduction mechanisms	1.67E-08	-3.56
SAUSA300_0068	-	Lead, cadmium, zinc and mercury transporting ATPase (E	Inorganic ion transport and metabolism	7.77E-03	4.49
SAUSA300_0072	-	Hypothetical protein	Coenzyme transport and metabolism	8.09E-04	9.20
SAUSA300_0073	-	Nickel-binding protein	Amino acid transport and metabolism	7.95E-07	5.49
SAUSA300_0074	opp-3B	Hypothetical protein	Amino acid transport and metabolism	4.88E-10	7.01
SAUSA300_0076	-	Hypothetical protein	Amino acid transport and metabolism	2.34E-14	10.63
SAUSA300_0077	-	Nickel transport ATP-binding protein nikE	Amino acid transport and metabolism	3.44E-06	15.76
SAUSA300_0078	copA	Copper-silver efflux ATPase (EC 3.6.3.-)	Inorganic ion transport and metabolism	2.51E-08	-9.41
SAUSA300_0083	-	Hypothetical membrane spanning protein	---	3.30E-12	3.30
SAUSA300_0084	-	Hypothetical cytosolic protein	Function unknown	3.00E-04	9.52
SAUSA300_0085	-	Rhodanese-related sulfurtransferases / Hypothetical protei	Function unknown	3.24E-10	12.19
SAUSA300_0086	-	Hydroxyacylglutathione hydrolase (EC 3.1.2.6)	General function prediction only	3.05E-10	10.35
SAUSA300_0092	-	Trp repressor binding protein	General function prediction only	1.03E-02	5.39
SAUSA300_0095	-	Transcriptional regulators, LysR family	Transcription	4.52E-04	2.75
SAUSA300_0101	-	membrane lipoprotein	---	5.42E-05	16.43
SAUSA300_0109	-	Transporter, drug/metabolite exporter family	---	6.02E-10	23.83
SAUSA300_0110	-	Transcriptional regulator, GntR family / TYROSINE AMI	Transcription	9.93E-07	2.92
SAUSA300_0111	-	Hypothetical protein	---	4.51E-08	-13.20
SAUSA300_0113	-	Immunoglobulin G binding protein A precursor	---	4.81E-05	3.98
SAUSA300_0115	sirC	Staphylobactin transport system permease protein	Inorganic ion transport and metabolism	1.42E-04	10.67
SAUSA300_0116	sirB	Staphylobactin transport system permease protein	Inorganic ion transport and metabolism	4.32E-13	25.94
SAUSA300_0117	sirA	Staphylobactin-binding protein	---	2.78E-09	36.21
SAUSA300_0118	-	Cysteine synthase (EC 2.5.1.47)	Amino acid transport and metabolism	6.10E-15	124.60
SAUSA300_0119	-	Ornithine cyclodeaminase family protein	Amino acid transport and metabolism	1.62E-17	47.62
SAUSA300_0120	sbnC	Siderophore biosynthesis IucC protein (EC 6.-.-.-)	Secondary metabolites biosynthesis, transpor	9.93E-11	24.32
SAUSA300_0121	-	Multidrug resistance efflux pump	---	9.03E-12	40.08
SAUSA300_0122	-	Amino-acid citrate synthetase (EC 6.-.-.-)	Secondary metabolites biosynthesis, transpor	6.14E-09	11.84
SAUSA300_0123	-	Siderophore synthase (EC 6.-.-.-)	Secondary metabolites biosynthesis, transpor	1.43E-13	13.71
SAUSA300_0124	-	4-hydroxy-2-oxovalerate aldolase (EC 4.1.2.-)	Carbohydrate transport and metabolism	5.21E-08	6.60
SAUSA300_0125	-	Diaminopimelate decarboxylase (EC 4.1.1.20)	Amino acid transport and metabolism	1.42E-13	25.66
SAUSA300_0126	-	Siderphore biosynthesis protein SbnI	Transcription	1.95E-11	30.10
SAUSA300_0130	-	Hypothetical protein	Cell wall/membrane biogenesis	5.32E-06	16.34
SAUSA300_0131	-	Undecaprenyl-phosphate galactosephosphotransferase (EC	Cell wall/membrane biogenesis	4.58E-08	9.31
SAUSA300_0132	-	beta-D-Glcp alpha-1,6-galactosyltransferase (EC 2.4.1.-)	Cell wall/membrane biogenesis	5.62E-12	7.10
SAUSA300_0133	-	Secreted polysaccharide polymerase	---	2.50E-05	5.49
SAUSA300_0134	-	Transcriptional activator amrA	General function prediction only	7.58E-04	2.57
SAUSA300_0135	-	Superoxide dismutase (EC 1.15.1.1)	Inorganic ion transport and metabolism	1.70E-07	24.73
SAUSA300_0137	-	Transcriptional regulator, GntR family	Transcription	8.51E-03	-4.67
SAUSA300_0138	deoD	Purine nucleoside phosphorylase (EC 2.4.2.1)	Nucleotide transport and metabolism	9.38E-05	19.18
SAUSA300_0139	-	Permease	---	2.80E-04	16.67
SAUSA300_0142	phnE	Phosphonates transport system permease protein phnE	Inorganic ion transport and metabolism	2.23E-12	10.44
SAUSA300_0143	phnE	Phosphonates transport system permease protein phnE	Inorganic ion transport and metabolism	3.73E-06	34.99
SAUSA300_0144	phnC	Phosphonates transport ATP-binding protein phnC	Inorganic ion transport and metabolism	2.84E-11	25.77
SAUSA300_0146	-	Hypothetical protein	---	2.08E-08	6.25
SAUSA300_0151	adhE	Alcohol dehydrogenase (EC 1.1.1.1) / Acetaldehyde dehyd	Energy production and conversion	8.82E-09	9.79
SAUSA300_0152	cap5A	Hypothetical protein	Cell wall/membrane biogenesis	2.21E-04	2.59
SAUSA300_0154	cap5C	Phosphotyrosine-protein phosphatase (capsular polysaccha	Carbohydrate transport and metabolism	1.06E-07	5.61
SAUSA300_0156	cap5E	UDP-N-acetylglucosamine 4,6-dehydratase (EC 4.2.1.-) /	Cell wall/membrane biogenesis	9.17E-08	33.39
SAUSA300_0157	cap5F	UDP-2-acetamido-2,6-dideoxy-beta-L-talose 4-dehydroge	Cell wall/membrane biogenesis	4.67E-08	12.83

6
5
4
3
2
1
-2
-3
-4
-5
-6

probe set mapping	synonym	description	NCBI COG	p-value	day one / inoculum
SAUSA300_0158	cap5G	UDP-2-acetamido-2,6-dideoxy-beta-L-talose 2-epimerase	Cell wall/membrane biogenesis	1.61E-05	12.16
SAUSA300_0159	cap5H	O-acetyl transferase (EC 2.3.1.-)	General function prediction only	5.51E-06	18.66
SAUSA300_0163	cap5L	Hypothetical protein	Cell wall/membrane biogenesis	9.44E-03	2.98
SAUSA300_0164	cap5M	Undecaprenyl-phosphate beta-N-acetyl-D-fucosaminepho	Cell wall/membrane biogenesis	5.28E-03	11.84
SAUSA300_0165	cap5N	UDP-N-acetyl-D-quinovosamine 4-epimerase (EC 5.1.3.-)	Cell wall/membrane biogenesis	3.05E-04	5.07
SAUSA300_0168	-	Hypothetical protein	General function prediction only	9.35E-04	4.94
SAUSA300_0169	-	Hypothetical membrane spanning protein	Function unknown	1.22E-04	2.64
SAUSA300_0173	-	alpha-helical coiled-coil protein SrpF	---	5.79E-03	-2.48
SAUSA300_0179	-	NAD-dependent formate dehydrogenase (EC 1.2.1.2)	Energy production and conversion	4.90E-04	3.71
SAUSA300_0184	argB	Acetylglutamate kinase (EC 2.7.2.8)	Amino acid transport and metabolism	1.08E-08	9.04
SAUSA300_0186	argC	N-acetyl-gamma-glutamyl-phosphate reductase (EC 1.2.1	Amino acid transport and metabolism	9.04E-08	6.46
SAUSA300_0187	rocD	Ornithine aminotransferase (EC 2.6.1.13)	Amino acid transport and metabolism	1.30E-10	39.96
SAUSA300_0192	-	outer surface protein	Function unknown	6.93E-10	-56.15
SAUSA300_0193	murQ	Glucokinase regulatory protein	General function prediction only	3.92E-14	-9.64
SAUSA300_0194	-	PTS system, sucrose-specific IIBC component (EC 2.7.1.6	Carbohydrate transport and metabolism	9.97E-10	-17.50
SAUSA300_0195	-	Transcriptional regulator, RpiR family	Transcription	1.64E-11	-16.38
SAUSA300_0197	-	Hypothetical cytosolic protein	---	3.61E-03	2.69
SAUSA300_0200	-	Oligopeptide transport ATP-binding protein oppD / Oligop	General function prediction only	8.58E-07	45.61
SAUSA300_0201	-	Oligopeptide transport system permease protein oppB	Amino acid transport and metabolism	2.68E-05	9.76
SAUSA300_0203	-	Oligopeptide-binding protein oppA	Amino acid transport and metabolism	5.61E-06	10.16
SAUSA300_0207	-	LIPOPROTEIN NLPD/LPPB HOMOLOG	Cell wall/membrane biogenesis	2.94E-10	26.45
SAUSA300_0208	-	Hypothetical protein	Carbohydrate transport and metabolism	1.73E-07	7.24
SAUSA300_0209	-	Maltose/maltodextrin-binding protein	Carbohydrate transport and metabolism	1.61E-11	67.64
SAUSA300_0210	-	Maltodextrin transport system permease protein malC	Carbohydrate transport and metabolism	1.20E-04	11.66
SAUSA300_0212	-	NAD-dependent oxidoreductase	General function prediction only	4.11E-06	3.61
SAUSA300_0215	-	Hypothetical membrane spanning protein	Function unknown	2.09E-11	29.77
SAUSA300_0223	-	Hypothetical protein	---	6.78E-04	57.53
SAUSA300_0224	coa	Staphylocoagulase precursor	---	6.22E-08	12.31
SAUSA300_0230	-	Hypothetical membrane spanning protein	Function unknown	1.83E-06	6.15
SAUSA300_0232	-	Hypothetical cytosolic protein	Function unknown	1.98E-02	2.60
SAUSA300_0233	-	Hypothetical protein	---	4.35E-03	-2.31
SAUSA300_0235	-	L-lactate dehydrogenase (EC 1.1.1.27)	Energy production and conversion	3.12E-02	-2.07
SAUSA300_0236	-	PTS system, glucose-specific IIBC component (EC 2.7.1.6	Carbohydrate transport and metabolism	6.65E-11	23.45
SAUSA300_0238	-	Transcription antiterminator, BglG family / PTS system, n	Transcription	1.68E-07	3.38
SAUSA300_0239	-	PTS system, galactitol-specific IIA component (EC 2.7.1.6	Carbohydrate transport and metabolism	1.51E-04	6.51
SAUSA300_0241	-	PTS system, galactitol-specific IIC component (EC 2.7.1.6	Carbohydrate transport and metabolism	3.25E-07	8.85
SAUSA300_0242	gutB	Sorbitol dehydrogenase (EC 1.1.1.14)	Amino acid transport and metabolism	2.90E-03	7.79
SAUSA300_0244	-	Sorbitol dehydrogenase (EC 1.1.1.14)	Amino acid transport and metabolism	1.09E-06	2.37
SAUSA300_0251	-	CDP-ribitol ribitolphosphotransferase (EC 2.7.8.14)	Cell wall/membrane biogenesis	3.55E-03	-2.98
SAUSA300_0253	scdA	Nitric oxide-dependent regulator DnrN	Cell cycle control, mitosis and meiosis	3.56E-04	-3.24
SAUSA300_0256	-	Murein hydrolase exporter	General function prediction only	2.67E-03	3.06
SAUSA300_0257	-	Murein hydrolase export regulator	Cell wall/membrane biogenesis	1.52E-03	-7.42
SAUSA300_0259	-	PTS system, beta-glucoside-specific IIABC component (E	Carbohydrate transport and metabolism	2.73E-16	19.89
SAUSA300_0260	bglA	6-phospho-beta-glucosidase (EC 3.2.1.86)	Carbohydrate transport and metabolism	1.25E-11	8.69
SAUSA300_0261	-	Hypothetical protein	Coenzyme transport and metabolism	8.69E-07	3.64
SAUSA300_0262	rbsK	Ribokinase (EC 2.7.1.15)	Carbohydrate transport and metabolism	1.55E-06	2.89
SAUSA300_0267	-	Transposase	Replication, recombination and repair	3.57E-05	35.08
SAUSA300_0268	-	Multidrug resistance protein B	---	5.93E-13	4.87
SAUSA300_0269	-	Choloylglycine hydrolase (EC 3.5.1.24)	Cell wall/membrane biogenesis	1.90E-07	23.41
SAUSA300_0270	lytM	Peptidoglycan hydrolase	General function prediction only	2.06E-08	10.63
SAUSA300_0272	-	Hypothetical protein	---	1.39E-03	7.13
SAUSA300_0273	-	Hypothetical protein	---	2.52E-03	15.41
SAUSA300_0275	-	Hypothetical protein	---	9.44E-06	5.26
SAUSA300_0276	-	Hypothetical protein	---	5.15E-03	13.63
SAUSA300_0277	-	Secretory antigen precursor SsaA	---	3.22E-06	8.66
SAUSA300_0278	-	Hypothetical protein	Function unknown	5.48E-11	-3.08
SAUSA300_0280	-	Hypothetical protein	---	1.27E-04	5.71
SAUSA300_0282	-	Hypothetical membrane associated protein	Function unknown	3.69E-04	2.86
SAUSA300_0283	-	DNA segregation ATPase and related proteins (FtsK/SpoII	Cell cycle control, mitosis and meiosis	3.06E-02	3.12
SAUSA300_0284	-	Hypothetical protein	---	3.37E-06	10.64
SAUSA300_0286	-	Hypothetical protein	---	3.43E-02	4.22
SAUSA300_0287	-	Hypothetical protein	---	6.00E-03	2.05
SAUSA300_0289	-	Hypothetical cytosolic protein	---	4.10E-03	-2.01
SAUSA300_0290	-	Hypothetical protein	---	1.55E-02	11.99
SAUSA300_0292	-	Hypothetical protein	---	1.10E-04	2.10
SAUSA300_0294	-	Hypothetical cytosolic protein	---	5.37E-04	16.45
SAUSA300_0299	-	Hypothetical cytosolic protein	---	1.79E-06	-3.83
SAUSA300_0302	-	Hypothetical cytosolic protein	---	2.69E-04	3.73
SAUSA300_0303	-	Hypothetical protein	---	1.51E-04	14.58
SAUSA300_0306	brnQ	Branched-chain amino acid transport system carrier protei	Amino acid transport and metabolism	5.09E-03	8.03
SAUSA300_0307	-	Acid phosphatase (EC 3.1.3.2)	General function prediction only	9.33E-06	17.23
SAUSA300_0308	-	ABC transporter permease protein	Cell wall/membrane biogenesis	1.22E-10	-17.95
SAUSA300_0309	-	ABC transporter ATP-binding protein	Defense mechanisms/Virulence	2.42E-10	-2.05
SAUSA300_0310	pfoR	Transcriptional regulator pfoR	Carbohydrate transport and metabolism	4.58E-03	2.62
SAUSA300_0311	-	Ribokinase (EC 2.7.1.15)	Carbohydrate transport and metabolism	4.02E-11	21.02
SAUSA300_0312	-	Sugar kinases	Secondary metabolites biosynthesis, transpo	2.84E-10	4.17
SAUSA300_0313	-	Nucleoside permease nupC	Nucleotide transport and metabolism	1.17E-08	15.98
SAUSA300_0315	nanA	N-acetylneuraminate lyase (EC 4.1.3.3)	Amino acid transport and metabolism	1.87E-02	2.89
SAUSA300_0317	-	Transcriptional regulator, RpiR family	Transcription	1.80E-03	4.28
SAUSA300_0321	-	Acetyl esterase (EC 3.1.1.-)	Lipid transport and metabolism	1.98E-02	-2.03

probe set mapping	synonym	description	NCBI COG	p-value	day one / inoculum
SAUSA300_0322	-	Probable NADH-dependent flavin oxidoreductase yqiG (E	Energy production and conversion	1.47E-08	19.65
SAUSA300_0323	-	Hypothetical protein	---	2.96E-08	82.10
SAUSA300_0329	-	CPRD14 PROTEIN	Cell wall/membrane biogenesis	1.93E-07	-3.50
SAUSA300_0330	ulaA	Putative transport protein sgaT	Function unknown	9.21E-14	40.38
SAUSA300_0332	-	PTS system, mannitol-specific IIA component (EC 2.7.1.6	Carbohydrate transport and metabolism	1.20E-03	2.39
SAUSA300_0336	-	Hypothetical cytosolic protein	Function unknown	1.18E-05	9.94
SAUSA300_0340	-	FMN reductase (EC 1.5.1.29)	General function prediction only	3.20E-03	-11.12
SAUSA300_0342	-	Hypothetical protein	---	1.73E-04	32.14
SAUSA300_0343	-	Ribosomal-protein-serine acetyltransferase (EC 2.3.1.-)	Translation	1.19E-02	4.94
SAUSA300_0344	-	Lipoprotein	Inorganic ion transport and metabolism	4.89E-03	3.14
SAUSA300_0345	-	Dyp-type peroxidase family protein	Inorganic ion transport and metabolism	8.40E-11	16.12
SAUSA300_0346	-	High-affinity iron permease	Inorganic ion transport and metabolism	2.43E-09	2.56
SAUSA300_0348	-	Sec-independent protein translocase protein tatA	Intracellular trafficking and secretion	3.63E-04	3.10
SAUSA300_0350	-	Transcriptional regulator, Cro/CI family	Transcription	3.84E-04	8.28
SAUSA300_0357	metE	5-methyltetrahydropteroyltriglutamate--homocysteine met	Amino acid transport and metabolism	6.50E-05	9.61
SAUSA300_0358	-	5-methyltetrahydrofolate--homocysteine methyltransferase	Amino acid transport and metabolism	2.61E-08	9.84
SAUSA300_0359	-	Cystathionine beta-lyase (EC 4.4.1.8)	Amino acid transport and metabolism	4.23E-13	21.24
SAUSA300_0360	-	Cystathionine gamma-synthase (EC 2.5.1.48)	Amino acid transport and metabolism	1.25E-09	35.96
SAUSA300_0361	-	Chromosome partitioning protein parB	Transcription	1.79E-08	22.43
SAUSA300_0362	-	Mechanosensitive ion channel	Cell wall/membrane biogenesis	1.01E-02	4.41
SAUSA300_0363	-	Hypothetical cytosolic protein	Function unknown	8.64E-03	4.52
SAUSA300_0369	-	Hypothetical protein	Defense mechanisms/Virulence	2.45E-07	16.79
SAUSA300_0370	-	Exotoxin	---	1.20E-05	9.30
SAUSA300_0371	-	Hypothetical protein	Function unknown	1.07E-06	70.46
SAUSA300_0372	-	Hypothetical exported protein	Function unknown	3.39E-08	-3.13
SAUSA300_0374	-	Hypothetical membrane spanning protein	---	2.59E-10	-3.46
SAUSA300_0375	-	Phosphoglycerate mutase family protein	Carbohydrate transport and metabolism	1.62E-02	5.24
SAUSA300_0376	-	Hypothetical membrane spanning protein	---	6.96E-11	-2.58
SAUSA300_0378	-	Hypothetical protein	---	2.02E-08	-2.18
SAUSA300_0385	-	Transcriptional regulator	---	1.79E-06	-2.95
SAUSA300_0386	xpt	Xanthine phosphoribosyltransferase (EC 2.4.2.-)	Nucleotide transport and metabolism	1.27E-02	-2.15
SAUSA300_0387	pbuX	Xanthine permease	Nucleotide transport and metabolism	4.52E-08	-10.08
SAUSA300_0388	guaB	Inosine-5'-monophosphate dehydrogenase (EC 1.1.1.205)	Nucleotide transport and metabolism	6.86E-07	-2.33
SAUSA300_0396	set7	Exotoxin	---	6.36E-09	10.00
SAUSA300_0397	-	Exotoxin	---	1.47E-10	27.58
SAUSA300_0398	-	Exotoxin	---	3.27E-13	21.87
SAUSA300_0399	-	Exotoxin	---	1.06E-07	22.98
SAUSA300_0400	-	Hypothetical protein	---	1.41E-13	148.27
SAUSA300_0401	-	Exotoxin	---	1.45E-03	7.02
SAUSA300_0402	-	Exotoxin	---	7.53E-06	5.43
SAUSA300_0403	-	Exotoxin	---	3.15E-09	23.08
SAUSA300_0404	-	Exotoxin	---	6.16E-07	49.41
SAUSA300_0407	-	Exotoxin	---	2.36E-08	67.54
SAUSA300_0408	-	Hypothetical cytosolic protein	---	5.95E-07	5.35
SAUSA300_0410	-	membrane lipoprotein	---	2.10E-09	8.28
SAUSA300_0411	-	membrane lipoprotein	---	1.26E-04	5.55
SAUSA300_0413	-	membrane lipoprotein	---	1.98E-04	30.71
SAUSA300_0415	lpl3	membrane lipoprotein	---	1.94E-03	8.43
SAUSA300_0416	-	membrane lipoprotein	---	1.28E-06	15.75
SAUSA300_0417	-	membrane lipoprotein	---	5.36E-06	34.37
SAUSA300_0420	-	Hypothetical cytosolic protein	---	6.99E-08	-2.99
SAUSA300_0421	-	Hypothetical cytosolic protein	---	1.21E-03	7.56
SAUSA300_0427	-	Hypothetical cytosolic protein	Function unknown	5.80E-04	-7.58
SAUSA300_0432	-	Sodium/neurotransmitter symporter family protein	General function prediction only	1.28E-11	23.49
SAUSA300_0433	cysM	Cysteine synthase (EC 2.5.1.47)	Amino acid transport and metabolism	2.96E-07	11.04
SAUSA300_0434	metB	Cystathionine beta-lyase (EC 4.4.1.8) / Cystathionine gam	Amino acid transport and metabolism	3.66E-05	3.29
SAUSA300_0435	-	ABC transporter ATP-binding protein	Inorganic ion transport and metabolism	3.47E-02	3.60
SAUSA300_0438	-	Putative endopeptidase lytE precursor	General function prediction only	1.09E-08	-14.18
SAUSA300_0441	-	Acetyltransferase (EC 2.3.1.-)	General function prediction only	1.81E-02	3.28
SAUSA300_0442	-	Hypothetical membrane spanning protein	Function unknown	8.08E-05	5.56
SAUSA300_0443	-	Hypothetical membrane spanning protein	Function unknown	2.42E-02	6.37
SAUSA300_0444	gltC	Transcriptional regulatory protein gltC	Transcription	9.60E-07	8.29
SAUSA300_0445	gltB	Glutamate synthase [NADPH] large chain (EC 1.4.1.13)	Amino acid transport and metabolism	3.11E-09	8.54
SAUSA300_0446	gltD	Glutamate synthase [NADPH] small chain (EC 1.4.1.13)	Amino acid transport and metabolism	2.21E-11	23.67
SAUSA300_0458	-	Arginine decarboxylase (EC 4.1.1.19)	Amino acid transport and metabolism	8.95E-05	2.88
SAUSA300_0459	tmk	Thymidylate kinase (EC 2.7.4.9)	Nucleotide transport and metabolism	2.07E-03	-3.93
SAUSA300_0461	holB	DNA polymerase III, delta' subunit (EC 2.7.7.7)	Replication, recombination and repair	1.67E-08	-2.50
SAUSA300_0462	-	Phosphorelay inhibitor	Function unknown	6.95E-07	-10.09
SAUSA300_0463	-	Initiation-control protein	Function unknown	1.39E-05	-2.99
SAUSA300_0471	-	VEG protein	Function unknown	1.14E-03	-2.24
SAUSA300_0472	ipk	4-diphosphocytidyl-2-C-methyl-D-erythritol kinase (EC 2	Lipid transport and metabolism	5.25E-06	-5.15
SAUSA300_0474	-	Translation initiation inhibitor	Translation	1.88E-10	-5.64
SAUSA300_0475	-	Stage V sporulation protein G	Cell wall/membrane biogenesis	8.25E-15	-8.31
SAUSA300_0477	glmU	Glucosamine-1-phosphate acetyltransferase (EC 2.3.1.157	Cell wall/membrane biogenesis	3.37E-04	-5.60
SAUSA300_0480	pth	Peptidyl-tRNA hydrolase (EC 3.1.1.29)	Translation	5.48E-04	3.14
SAUSA300_0481	mfd	Transcription-repair coupling factor	Replication, recombination and repair	1.04E-04	3.52
SAUSA300_0483	-	Hypothetical protein	General function prediction only	5.19E-12	4.34
SAUSA300_0488	hpt	Hypoxanthine-guanine phosphoribosyltransferase (EC 2.4	Nucleotide transport and metabolism	2.72E-02	2.64
SAUSA300_0491	cysK	Cysteine synthase (EC 2.5.1.47)	Amino acid transport and metabolism	3.14E-04	-3.10
SAUSA300_0503	-	Transcriptional regulator, GntR family / AMINOTRANSF	Transcription	8.14E-09	6.55

probe set mapping	synonym	description	NCBI COG	p-value	day one / inoculum
SAUSA300_0505	-	pyridoxine biosynthesis amidotransferase (EC 2.4.2.-)	Coenzyme transport and metabolism	1.19E-06	-6.09
SAUSA300_0508	-	CLPC ATPASE	Function unknown	8.89E-05	8.62
SAUSA300_0509	-	Arginine kinase (EC 2.7.3.3)	Amino acid transport and metabolism	5.97E-03	8.83
SAUSA300_0510	clpC	Negative regulator of genetic competence clpC/mecB / He	---	1.32E-05	5.91
SAUSA300_0514	cysE	Serine acetyltransferase (EC 2.3.1.30)	Amino acid transport and metabolism	1.64E-03	-2.16
SAUSA300_0515	cysS	Cysteinyl-tRNA synthetase (EC 6.1.1.16)	Translation	7.92E-04	-2.19
SAUSA300_0516	-	Hypothetical protein	Function unknown	1.80E-06	-4.11
SAUSA300_0519	-	RNA polymerase sigma-H factor	Transcription	1.59E-06	-2.60
SAUSA300_0527	rpoB	DNA-directed RNA polymerase beta chain (EC 2.7.7.6)	Transcription	1.26E-10	-5.32
SAUSA300_0528	rpoC	DNA-directed RNA polymerase beta' chain (EC 2.7.7.6)	Transcription	4.00E-04	-2.93
SAUSA300_0535	-	2-amino-3-ketobutyrate coenzyme A ligase (EC 2.3.1.29)	Coenzyme transport and metabolism	9.07E-07	-2.77
SAUSA300_0537	-	L-ribulokinase (EC 2.7.1.16)	Energy production and conversion	4.27E-10	5.33
SAUSA300_0539	ilvE	Branched-chain amino acid aminotransferase (EC 2.6.1.42	Amino acid transport and metabolism	4.25E-09	3.93
SAUSA300_0542	-	Deoxyguanosine kinase (EC 2.7.1.113)	Nucleotide transport and metabolism	1.43E-06	-3.47
SAUSA300_0544	-	Hydrolase (HAD superfamily)	General function prediction only	3.14E-03	3.43
SAUSA300_0546	sdrC	Fibronectin-binding protein	---	2.10E-10	47.36
SAUSA300_0547	sdrD	Fibronectin-binding protein	---	3.08E-07	41.39
SAUSA300_0548	sdrE	Fibronectin-binding protein	---	4.76E-07	12.04
SAUSA300_0551	-	Hypothetical cytosolic protein	Function unknown	2.43E-04	-3.17
SAUSA300_0556	-	Hexulose-6-phosphate isomerase (EC 5.-.-.-)	Cell wall/membrane biogenesis	4.46E-13	-3.04
SAUSA300_0557	-	Putative phosphatase	General function prediction only	2.97E-10	24.85
SAUSA300_0559	-	Long-chain-fatty-acid--CoA ligase (EC 6.2.1.3)	Lipid transport and metabolism	1.85E-07	15.46
SAUSA300_0560	vraB	Acetyl-CoA acetyltransferase (EC 2.3.1.9)	Lipid transport and metabolism	7.60E-07	2.30
SAUSA300_0566	-	Arginine permease	Amino acid transport and metabolism	8.20E-13	-11.31
SAUSA300_0568	-	Threonine/Serine Exporter	Function unknown	3.13E-08	-8.31
SAUSA300_0569	-	Hypothetical protein	Function unknown	7.81E-03	3.31
SAUSA300_0572	mvk	Mevalonate kinase (EC 2.7.1.36)	Lipid transport and metabolism	2.09E-04	-8.58
SAUSA300_0574	-	Phosphomevalonate kinase (EC 2.7.4.2)	Lipid transport and metabolism	6.35E-10	-2.84
SAUSA300_0576	-	Pyridine nucleotide-disulphide oxidoreductase family prot	Energy production and conversion	1.19E-03	2.79
SAUSA300_0577	-	Rrf2 family protein	Transcription	8.95E-09	10.14
SAUSA300_0581	-	Hypothetical membrane spanning protein	---	3.78E-07	-4.98
SAUSA300_0582	-	Hypothetical membrane spanning protein	---	2.00E-02	-3.28
SAUSA300_0583	-	Hypothetical membrane spanning protein	---	1.37E-02	2.16
SAUSA300_0588	-	Hypothetical membrane spanning protein	---	2.68E-04	3.68
SAUSA300_0590	-	Trp repressor binding protein	General function prediction only	4.20E-05	-2.10
SAUSA300_0596	argS	Arginyl-tRNA synthetase (EC 6.1.1.19)	Translation	7.43E-07	-4.28
SAUSA300_0602	-	Hypothetical exported protein	---	2.85E-04	-4.72
SAUSA300_0603	-	Hypothetical protein	---	9.04E-07	-2.81
SAUSA300_0604	-	Esterase (EC 3.1.1.-)	General function prediction only	6.68E-11	-2.47
SAUSA300_0607	-	Hypothetical protein	---	2.48E-06	131.62
SAUSA300_0608	-	Hypothetical protein	---	6.30E-07	77.85
SAUSA300_0609	-	DNA integration/recombination/inversion protein	Replication, recombination and repair	4.65E-08	190.00
SAUSA300_0610	-	Sodium/proton antiporter protein shaA	Energy production and conversion	1.12E-11	138.67
SAUSA300_0611	-	Sodium/proton antiporter protein shaB	Inorganic ion transport and metabolism	5.80E-11	63.78
SAUSA300_0612	-	Sodium/proton antiporter protein shaC	Inorganic ion transport and metabolism	2.72E-03	7.36
SAUSA300_0613	-	Hypothetical protein	Energy production and conversion	9.77E-08	5.04
SAUSA300_0615	-	Sodium/proton antiporter protein shaF	Inorganic ion transport and metabolism	7.53E-05	9.58
SAUSA300_0620	-	Manganese transport system ATP-binding protein	Inorganic ion transport and metabolism	4.55E-03	-3.08
SAUSA300_0621	-	Iron-dependent repressor	Transcription	5.39E-06	-4.76
SAUSA300_0622	-	Hypothetical membrane spanning protein	---	1.79E-11	26.19
SAUSA300_0626	tagB	Teichoic acid biosynthesis protein B precursor	Cell wall/membrane biogenesis	1.13E-05	-3.99
SAUSA300_0627	tagX	TAGX	General function prediction only	4.88E-04	-2.33
SAUSA300_0629	pbp4	Hypothetical protein	Cell wall/membrane biogenesis	1.95E-02	4.94
SAUSA300_0632	-	Hypothetical membrane spanning protein	Function unknown	1.03E-04	-4.30
SAUSA300_0634	fhuB	Ferrichrome transport system permease protein fhuB	Inorganic ion transport and metabolism	3.13E-07	2.52
SAUSA300_0638	-	Dihydroxyacetone kinase phosphotransfer protein	Function unknown	2.54E-04	-2.37
SAUSA300_0643	-	Acetyltransferase, GNAT family	Translation	1.79E-02	-5.27
SAUSA300_0646	-	Hypothetical protein	Signal transduction mechanisms	4.58E-06	-4.92
SAUSA300_0647	-	Bacitracin transport ATP-binding protein ytsC	Defense mechanisms/Virulence	1.55E-04	2.07
SAUSA300_0648	-	ABC transporter permease protein	---	3.85E-03	-2.04
SAUSA300_0650	-	Low-affinity inorganic phosphate transporter	Inorganic ion transport and metabolism	3.28E-08	-20.89
SAUSA300_0651	-	Secretory antigen precursor SsaA	---	9.16E-05	-2.72
SAUSA300_0653	-	Transcriptional regulator, AraC family	Transcription	5.60E-03	-2.37
SAUSA300_0664	-	Hypothetical protein	---	9.53E-11	-30.96
SAUSA300_0667	-	EMG2 protein	Function unknown	3.35E-12	-3.54
SAUSA300_0673	-	Low-affinity zinc transport protein	General function prediction only	3.50E-05	-2.46
SAUSA300_0674	-	Oxidoreductase (EC 1.1.1.-)	General function prediction only	5.65E-08	-3.78
SAUSA300_0675	-	Hypothetical protein	---	4.75E-06	-2.71
SAUSA300_0678	-	Permease	Function unknown	2.86E-02	3.11
SAUSA300_0680	norA	Quinolone resistance protein norA	---	2.51E-08	-4.27
SAUSA300_0681	-	Hypothetical protein	---	2.88E-05	-3.85
SAUSA300_0682	ybaK	Regulatory protein	Function unknown	2.68E-05	2.85
SAUSA300_0683	-	Fructose repressor	Transcription	4.10E-10	13.81
SAUSA300_0684	fruB	1-phosphofructokinase (EC 2.7.1.56)	Carbohydrate transport and metabolism	5.42E-15	11.54
SAUSA300_0685	fruA	PTS system, fructose-specific IIABC component (EC 2.7.	Carbohydrate transport and metabolism	3.88E-07	8.88
SAUSA300_0687	-	Magnesium and cobalt efflux protein corC	General function prediction only	1.69E-07	-2.94
SAUSA300_0688	-	2,5-diketo-D-gluconic acid reductase (EC 1.1.1.274)	General function prediction only	1.25E-09	-9.06
SAUSA300_0689	-	Undecaprenyl-phosphate beta-N-acetylglucosaminyltransf	Cell wall/membrane biogenesis	3.21E-14	-5.67
SAUSA300_0694	-	Hypothetical protein	---	2.07E-02	2.39
SAUSA300_0697	-	Queuosine biosynthesis protein QueC	General function prediction only	1.97E-02	11.12

probe set mapping	synonym	description	NCBI COG	p-value	day one / inoculum
SAUSA300_0698	pabA	Anthranilate synthase component II (EC 4.1.3.27) / Para-a	Amino acid transport and metabolism	9.46E-04	10.99
SAUSA300_0699	-	Para-aminobenzoate synthetase component I (EC 6.3.5.8)	Amino acid transport and metabolism	2.18E-03	2.11
SAUSA300_0703	-	Sulfatase family protein	Cell wall/membrane biogenesis	1.63E-03	-2.46
SAUSA300_0706	-	Glycine betaine transport ATP-binding protein	Amino acid transport and metabolism	8.32E-04	-2.08
SAUSA300_0710	-	Hypothetical protein	---	4.27E-10	14.69
SAUSA300_0714	-	Transporter, drug/metabolite exporter family	---	4.40E-05	-2.89
SAUSA300_0715	nrdI	NrdI protein	Nucleotide transport and metabolism	2.80E-06	-13.55
SAUSA300_0716	-	Ribonucleoside-diphosphate reductase alpha chain (EC 1.	Nucleotide transport and metabolism	1.36E-04	-3.27
SAUSA300_0717	nrdF	Ribonucleoside-diphosphate reductase beta chain (EC 1.1	Nucleotide transport and metabolism	1.06E-07	-21.54
SAUSA300_0718	-	Ferric anguibactin transport system permease protein fatD	Inorganic ion transport and metabolism	1.23E-11	3.09
SAUSA300_0719	-	Ferric anguibactin transport system permease protein fatC	Inorganic ion transport and metabolism	8.90E-03	3.98
SAUSA300_0720	-	Ferric anguibactin transport ATP-binding protein	Inorganic ion transport and metabolism	1.43E-07	16.72
SAUSA300_0721	-	Ferric anguibactin-binding protein	Inorganic ion transport and metabolism	9.18E-07	21.22
SAUSA300_0723	-	Glutamate-rich protein grpB	---	2.64E-07	2.39
SAUSA300_0725	-	Hypothetical cytosolic protein	---	3.10E-07	-5.13
SAUSA300_0726	-	Glycerate kinase (EC 2.7.1.31)	Carbohydrate transport and metabolism	1.55E-02	-2.27
SAUSA300_0729	-	Threonine/Serine Exporter	Function unknown	3.56E-04	2.17
SAUSA300_0731	-	Undecaprenyl-phosphate alpha-N-acetylglucosaminephos	Cell wall/membrane biogenesis	8.27E-06	3.83
SAUSA300_0732	-	Hypothetical protein	Function unknown	4.95E-03	3.21
SAUSA300_0734	-	COMF operon protein 1	Replication, recombination and repair	9.88E-10	25.76
SAUSA300_0735	-	COMF operon protein 3	---	7.34E-03	2.96
SAUSA300_0739	-	Secretory antigen precursor SsaA	General function prediction only	1.65E-09	18.73
SAUSA300_0741	uvrB	Excinuclease ABC subunit B	Replication, recombination and repair	8.76E-06	-2.77
SAUSA300_0743	hprK	HPR(SER) KINASE (EC 2.7.1.-) / PHOSPHATASE (EC	Signal transduction mechanisms	1.07E-08	-6.13
SAUSA300_0744	lgt	Prolipoprotein diacylglyceryl transferase (EC 2.4.99.-)	Cell wall/membrane biogenesis	2.63E-07	-2.53
SAUSA300_0745	-	O-acetyltransferase (EC 2.3.1.-)	General function prediction only	5.95E-05	-2.56
SAUSA300_0746	-	Tetratricopeptide repeat family protein	General function prediction only	6.72E-04	-2.48
SAUSA300_0747	trxB	Thioredoxin reductase (EC 1.8.1.9)	Posttranslational modification, protein turno	2.80E-02	2.24
SAUSA300_0749	-	Hypothetical membrane associated protein	Function unknown	7.87E-09	-6.96
SAUSA300_0750	-	Hypothetical cytosolic protein	Function unknown	2.82E-11	-2.64
SAUSA300_0753	-	Cell division inhibitor	General function prediction only	1.99E-07	-3.99
SAUSA300_0754	-	Hypothetical protein	---	1.24E-08	-2.19
SAUSA300_0757	pgk	Phosphoglycerate kinase (EC 2.7.2.3)	Carbohydrate transport and metabolism	1.24E-05	-2.37
SAUSA300_0758	tpiA	Triosephosphate isomerase (EC 5.3.1.1)	Carbohydrate transport and metabolism	3.62E-04	-2.12
SAUSA300_0759	gpmI	Phosphoglycerate mutase (EC 5.4.2.1)	Carbohydrate transport and metabolism	3.26E-09	-2.07
SAUSA300_0760	eno	Enolase (EC 4.2.1.11)	Carbohydrate transport and metabolism	2.13E-03	-2.31
SAUSA300_0761	-	Hypothetical membrane spanning protein	---	1.76E-06	-3.45
SAUSA300_0762	secG	Protein translocase subunit secG	Intracellular trafficking and secretion	3.26E-04	-2.17
SAUSA300_0765	smpB	SsrA-binding protein	Posttranslational modification, protein turno	1.63E-09	-2.04
SAUSA300_0767	-	Hypothetical protein	---	6.91E-03	2.86
SAUSA300_0768	-	Hypothetical protein	---	2.79E-03	-2.42
SAUSA300_0771	-	Acetyltransferase (EC 2.3.1.-)	General function prediction only	1.48E-04	-6.87
SAUSA300_0772	clfA	Fibronectin-binding protein	---	7.92E-16	-6.52
SAUSA300_0773	-	Staphylocoagulase precursor	---	4.63E-06	34.52
SAUSA300_0774	empbp	Extracellular matrix binding protein / Fibrinogen-binding	---	3.36E-07	11.71
SAUSA300_0775	-	extracellular matrix and plasma binding protein	---	9.40E-09	20.72
SAUSA300_0777	-	Cold shock protein	Transcription	1.37E-10	-22.62
SAUSA300_0778	-	Hypothetical protein	---	2.41E-03	-2.09
SAUSA300_0779	-	Phage protein	---	3.87E-05	-2.17
SAUSA300_0780	-	Hypothetical exported protein	Function unknown	4.00E-04	2.38
SAUSA300_0783	-	Phosphoglycerate mutase family protein	Carbohydrate transport and metabolism	6.88E-05	5.85
SAUSA300_0786	-	Organic hydroperoxide resistance protein	Posttranslational modification, protein turno	6.48E-11	-61.85
SAUSA300_0787	aroD	3-dehydroquinate dehydratase (EC 4.2.1.10)	Amino acid transport and metabolism	3.57E-02	13.01
SAUSA300_0788	-	Nitroreductase family	Energy production and conversion	4.52E-04	2.81
SAUSA300_0792	-	Hypothetical protein	---	2.28E-02	8.52
SAUSA300_0794	-	DNA primase (bacterial type) and small primase-like prote	Replication, recombination and repair	7.21E-07	-9.72
SAUSA300_0796	-	ABC transporter ATP-binding protein	Inorganic ion transport and metabolism	1.74E-06	8.65
SAUSA300_0797	-	ABC transporter permease protein	Inorganic ion transport and metabolism	2.86E-04	2.40
SAUSA300_0799	int	DNA integration/recombination/inversion protein	Replication, recombination and repair	2.76E-06	13.11
SAUSA300_0800	sek	Enterotoxin	---	5.62E-03	-2.57
SAUSA300_0802	-	Zn-dependent alcohol dehydrogenases and related dehydro	---	5.93E-04	-2.80
SAUSA300_0804	-	Transcriptional regulator, Cro family	---	1.00E-03	3.89
SAUSA300_0809	-	DNA primase	General function prediction only	1.02E-02	3.58
SAUSA300_0811	-	Hypothetical protein	---	1.39E-02	3.10
SAUSA300_0813	-	Hypothetical protein	---	2.94E-04	2.46
SAUSA300_0815	ear	Hypothetical protein	---	3.47E-07	7.26
SAUSA300_0817	-	Integral membrane protein	Function unknown	2.68E-06	-2.16
SAUSA300_0819	sufD	SufD protein	Posttranslational modification, protein turno	8.21E-08	-4.32
SAUSA300_0826	-	Hypothetical cytosolic protein	Function unknown	1.47E-02	-2.70
SAUSA300_0828	-	5'-nucleotidase (EC 3.1.3.5)	Nucleotide transport and metabolism	6.17E-04	-4.16
SAUSA300_0832	-	Hypothetical cytosolic protein	Function unknown	3.44E-06	-3.67
SAUSA300_0833	-	Hydrolase (HAD superfamily)	Carbohydrate transport and metabolism	4.15E-06	-2.33
SAUSA300_0834	-	Glyoxylate reductase (NADP+) (EC 1.1.1.79) / Glyoxylat	Energy production and conversion	1.84E-06	-5.90
SAUSA300_0836	dltB	Protein dltB	Cell wall/membrane biogenesis	1.25E-04	-3.21
SAUSA300_0839	-	NifU-like protein	Posttranslational modification, protein turno	2.11E-02	3.09
SAUSA300_0840	-	Hypothetical protein	Function unknown	2.06E-02	2.57
SAUSA300_0844	-	NADH dehydrogenase family	Energy production and conversion	5.64E-08	-2.96
SAUSA300_0848	-	Thioredoxin reductase/alkyl hydroperoxide reductase	Energy production and conversion	2.64E-07	93.90
SAUSA300_0849	mnhG	Sodium/proton antiporter protein shaG	Inorganic ion transport and metabolism	1.87E-02	-2.41
SAUSA300_0850	mnhF	Sodium/proton antiporter protein shaF	Inorganic ion transport and metabolism	8.63E-03	2.31

probe set mapping	synonym	description	NCBI COG	p-value	day one / inoculum
SAUSA300_0858	-	S1-type RNA-binding domain	Translation	4.02E-09	-5.32
SAUSA300_0860	rocD	Ornithine aminotransferase (EC 2.6.1.13)	Amino acid transport and metabolism	5.40E-12	-2.83
SAUSA300_0861	gudB	NAD-specific glutamate dehydrogenase (EC 1.4.1.2)	Amino acid transport and metabolism	1.96E-07	-3.16
SAUSA300_0862	glpQ	Glycerophosphoryl diester phosphodiesterase (EC 3.1.4.46	Energy production and conversion	2.13E-03	2.11
SAUSA300_0863	argH	Argininosuccinate lyase (EC 4.3.2.1)	Amino acid transport and metabolism	6.01E-11	12.69
SAUSA300_0864	argG	Argininosuccinate synthase (EC 6.3.4.5)	Amino acid transport and metabolism	1.08E-11	10.95
SAUSA300_0872	-	Hypothetical protein	---	1.08E-09	-2.05
SAUSA300_0874	-	Hydrolase (HAD superfamily)	General function prediction only	2.77E-05	-4.41
SAUSA300_0876	-	Acyltransferase family	Lipid transport and metabolism	1.23E-04	3.08
SAUSA300_0877	clpB	ClpB protein	Posttranslational modification, protein turnov	7.84E-06	13.16
SAUSA300_0878	-	Citrate synthase I repressor	Transcription	2.13E-05	33.31
SAUSA300_0879	-	2-isopropylmalate synthase (EC 2.3.3.13)	Amino acid transport and metabolism	2.16E-11	16.35
SAUSA300_0880	-	SUA5 protein	Translation	6.16E-08	36.90
SAUSA300_0881	-	Major Facilitator Superfamily	---	9.23E-03	5.79
SAUSA300_0882	-	Putative phosphatidylethanolamine-binding protein	General function prediction only	3.13E-07	3.01
SAUSA300_0883	-	Outer membrane protein	---	6.18E-09	-2.79
SAUSA300_0885	fabH	3-oxoacyl-[acyl-carrier-protein] synthase III (EC 2.3.1.41)	Lipid transport and metabolism	1.02E-08	4.92
SAUSA300_0887	oppB	Oligopeptide transport system permease protein oppB	Amino acid transport and metabolism	5.51E-09	-9.30
SAUSA300_0890	oppF	Oligopeptide transport ATP-binding protein oppF	General function prediction only	4.68E-06	-3.50
SAUSA300_0891	oppA	Oligopeptide-binding protein oppA	Amino acid transport and metabolism	3.10E-08	-4.85
SAUSA300_0892	oppA	Oligopeptide-binding protein oppA	Amino acid transport and metabolism	3.97E-10	86.20
SAUSA300_0893	oppF	Oligopeptide transport ATP-binding protein oppD	Amino acid transport and metabolism	7.16E-07	29.61
SAUSA300_0894	oppD	Oligopeptide transport ATP-binding protein oppF	Amino acid transport and metabolism	5.17E-06	28.79
SAUSA300_0896	oppC	Oligopeptide transport system permease protein oppC	Amino acid transport and metabolism	4.03E-08	4.53
SAUSA300_0897	trpS	Tryptophanyl-tRNA synthetase (EC 6.1.1.2)	Translation	8.21E-03	3.09
SAUSA300_0900	-	Putative competence protein/transcription factor	General function prediction only	5.02E-13	61.81
SAUSA300_0901	-	Putative competence protein/transcription factor	General function prediction only	1.69E-08	37.56
SAUSA300_0902	pepF	Oligoendopeptidase F (EC 3.4.24.-)	Amino acid transport and metabolism	5.01E-04	3.56
SAUSA300_0903	-	Hypothetical cytosolic protein	Secondary metabolites biosynthesis, transpor	1.12E-06	-2.08
SAUSA300_0907	-	GTP pyrophosphokinase homolog	Function unknown	1.82E-02	2.93
SAUSA300_0909	-	Ribosomal large subunit pseudouridine synthase D (EC 4.	Translation	2.39E-04	-2.45
SAUSA300_0914	-	Sodium/proton-dependent alanine carrier protein	Amino acid transport and metabolism	2.08E-09	3.84
SAUSA300_0916	-	2'-5' RNA ligase (EC 6.5.1.-)	Translation	6.77E-08	-2.12
SAUSA300_0918	-	1,2-diacylglycerol 3-glucosyltransferase (EC 2.4.1.157) / g	Cell wall/membrane biogenesis	1.29E-03	-3.02
SAUSA300_0920	-	Hypothetical protein	---	1.94E-05	-4.31
SAUSA300_0922	-	Tellurite resistance protein terC	Inorganic ion transport and metabolism	1.76E-06	6.72
SAUSA300_0923	htrA	Endopeptidase degP (EC 3.4.21.-)	Posttranslational modification, protein turnov	3.92E-11	3.04
SAUSA300_0924	-	Potassium uptake protein ktrB	Inorganic ion transport and metabolism	5.65E-03	4.82
SAUSA300_0930	-	Lipoate-protein ligase A (EC 6.3.2.-)	Coenzyme transport and metabolism	1.50E-04	9.83
SAUSA300_0934	-	Hypothetical protein	Function unknown	6.65E-09	15.88
SAUSA300_0936	-	ABC transporter ATP-binding protein	Defense mechanisms/Virulence	1.48E-09	23.88
SAUSA300_0941	-	Iron(III) dicitrate-binding protein	Inorganic ion transport and metabolism	6.99E-04	9.88
SAUSA300_0942	-	Hypothetical membrane spanning protein	Function unknown	1.51E-07	4.61
SAUSA300_0944	menA	1,4-dihydroxy-2-naphthoate polyprenyltransferase (EC 2.5	Coenzyme transport and metabolism	1.74E-04	2.39
SAUSA300_0947	-	Menaquinone biosynthesis related protein	General function prediction only	1.14E-02	-3.12
SAUSA300_0950	sspB	Staphopain (EC 3.4.22.-)	---	1.13E-06	-5.80
SAUSA300_0952	-	Aromatic amino acid aminotransferase (EC 2.6.1.57) / Ace	Amino acid transport and metabolism	5.13E-10	2.17
SAUSA300_0956	-	Acetyltransferase, GNAT family	General function prediction only	5.47E-08	12.70
SAUSA300_0959	fmt	Beta-lactamase family protein	Defense mechanisms/Virulence	2.01E-02	11.71
SAUSA300_0960	qoxD	Cytochrome aa3 quinol oxidase polypeptide IV (EC 1.9.3.	Energy production and conversion	6.88E-07	-13.20
SAUSA300_0961	qoxC	Cytochrome aa3 quinol oxidase polypeptide III (EC 1.9.3.	Energy production and conversion	1.25E-06	-3.12
SAUSA300_0962	qoxB	Cytochrome aa3 quinol oxidase polypeptide I (EC 1.9.3.-)	Energy production and conversion	9.22E-11	-2.49
SAUSA300_0963	qoxA	Cytochrome aa3 quinol oxidase polypeptide II (EC 1.9.3.-	Energy production and conversion	5.63E-09	-3.40
SAUSA300_0966	purE	Phosphoribosylaminoimidazole carboxylase carboxyltrans	Nucleotide transport and metabolism	1.43E-10	-7.11
SAUSA300_0967	purK	Phosphoribosylaminoimidazole carboxylase NCAIR muta	Nucleotide transport and metabolism	1.57E-08	-3.14
SAUSA300_0968	purC	Phosphoribosylamidoimidazole-succinocarboxamide syntl	Nucleotide transport and metabolism	4.99E-08	-2.32
SAUSA300_0969	purS	Phosphoribosylformylglycinamidine synthase, purS compo	Nucleotide transport and metabolism	2.22E-10	-10.50
SAUSA300_0970	purQ	Phosphoribosylformylglycinamidine synthase (EC 6.3.5.3	Nucleotide transport and metabolism	1.99E-10	-28.25
SAUSA300_0971	purL	Phosphoribosylformylglycinamidine synthase (EC 6.3.5.3	Nucleotide transport and metabolism	1.22E-10	-4.03
SAUSA300_0972	purF	Amidophosphoribosyltransferase (EC 2.4.2.14)	Nucleotide transport and metabolism	2.43E-15	-24.54
SAUSA300_0973	purM	Phosphoribosylformylglycinamidine cyclo-ligase (EC 6.3.	Nucleotide transport and metabolism	3.25E-13	-7.26
SAUSA300_0974	purN	Phosphoribosylglycinamide formyltransferase (EC 2.1.2.2	Nucleotide transport and metabolism	1.07E-13	-15.31
SAUSA300_0975	purH	Phosphoribosylaminoimidazolecarboxamide formyltransfe	Nucleotide transport and metabolism	1.26E-10	-44.59
SAUSA300_0976	purD	Phosphoribosylamine--glycine ligase (EC 6.3.4.13)	Nucleotide transport and metabolism	6.00E-12	-4.46
SAUSA300_0977	-	Hydroxymethylpyrimidine transport system permease prot	Inorganic ion transport and metabolism	5.39E-03	4.51
SAUSA300_0978	-	Hydroxymethylpyrimidine transport ATP-binding protein	General function prediction only	8.37E-08	3.65
SAUSA300_0979	-	Hydroxymethylpyrimidine transport system permease prot	Function unknown	3.27E-08	22.40
SAUSA300_0982	-	Radical activating enzymes proteins	---	1.42E-12	-4.02
SAUSA300_0985	-	Hypothetical protein	Posttranslational modification, protein turnov	1.89E-04	18.59
SAUSA300_0986	-	Cytochrome d ubiquinol oxidase subunit I (EC 1.10.3.-)	Energy production and conversion	1.43E-08	11.38
SAUSA300_0990	-	Putative transcriptional regulator	Function unknown	6.27E-04	-4.37
SAUSA300_0991	def	Peptide deformylase (EC 3.5.1.88)	Translation	4.81E-04	4.11
SAUSA300_0992	-	Hypothetical protein	---	7.77E-03	16.04
SAUSA300_0995	-	Dihydrolipoamide acetyltransferase component of pyruvat	Energy production and conversion	1.38E-03	-2.19
SAUSA300_0999	potA	Spermidine/putrescine transport ATP-binding protein potA	Amino acid transport and metabolism	4.53E-03	10.17
SAUSA300_1003	-	Hypothetical protein	---	2.30E-11	-8.37
SAUSA300_1004	-	Hypothetical protein	---	2.08E-02	2.21
SAUSA300_1005	-	Manganese transport protein mntH	Inorganic ion transport and metabolism	7.06E-03	-5.11
SAUSA300_1009	typA	GTP-binding protein TypA/BipA	Signal transduction mechanisms	1.42E-05	-5.12
SAUSA300_1015	ctaA	Heme O monooxygenase (EC 1.-.-.-)	Posttranslational modification, protein turnov	4.50E-05	-4.56

probe set mapping	synonym	description	NCBI COG	p-value	day one / inoculum
SAUSA300_1017	-	Hypothetical membrane spanning protein	Function unknown	5.89E-05	-6.25
SAUSA300_1020	-	Glycerophosphoryl diester phosphodiesterase (EC 3.1.4.4(	Energy production and conversion	1.79E-09	5.50
SAUSA300_1027	rpmF	LSU ribosomal protein L32P	Translation	2.92E-03	2.02
SAUSA300_1028	-	Fibronectin-binding protein	---	9.96E-10	124.58
SAUSA300_1029	-	Fibronectin-binding protein	---	2.95E-06	13.90
SAUSA300_1030	-	Cell surface protein	---	1.20E-09	13.52
SAUSA300_1031	-	Hypothetical membrane associated protein	---	6.34E-11	44.84
SAUSA300_1032	-	Ferrichrome-binding protein	Inorganic ion transport and metabolism	3.44E-10	38.15
SAUSA300_1033	-	Transporter	Inorganic ion transport and metabolism	1.19E-12	11.64
SAUSA300_1034	srtB	Sortase B family protein	Function unknown	3.63E-07	13.73
SAUSA300_1035	-	Hypothetical cytosolic protein	General function prediction only	2.67E-05	16.32
SAUSA300_1036	-	23S rRNA methyltransferase (EC 2.1.1.-)	Translation	5.96E-03	2.43
SAUSA300_1037	pheS	Phenylalanyl-tRNA synthetase alpha chain (EC 6.1.1.20)	Translation	6.34E-06	5.27
SAUSA300_1046	sdhC	Succinate dehydrogenase cytochrome b558 subunit (EC 1.	Energy production and conversion	1.65E-07	-2.23
SAUSA300_1047	sdhA	Succinate dehydrogenase flavoprotein subunit (EC 1.3.99.	Energy production and conversion	8.24E-10	-2.26
SAUSA300_1048	sdhB	Succinate dehydrogenase iron-sulfur protein (EC 1.3.99.1)	Energy production and conversion	2.91E-10	-3.31
SAUSA300_1051	-	putative phosphoesterase	General function prediction only	9.50E-07	-5.09
SAUSA300_1053	-	Hypothetical protein	---	5.41E-04	8.35
SAUSA300_1054	-	Hypothetical membrane spanning protein	---	2.29E-06	4.47
SAUSA300_1057	-	Hypothetical protein	---	2.61E-04	-3.71
SAUSA300_1059	-	Exotoxin	---	2.99E-14	23.97
SAUSA300_1060	-	Exotoxin	---	4.95E-10	6.88
SAUSA300_1061	-	Exotoxin	---	1.49E-08	35.50
SAUSA300_1062	argF	Ornithine carbamoyltransferase (EC 2.1.3.3)	Amino acid transport and metabolism	7.78E-06	12.12
SAUSA300_1063	arcC	Carbamate kinase (EC 2.7.2.2)	Amino acid transport and metabolism	1.25E-07	10.69
SAUSA300_1067	-	Antibacterial protein 3	---	2.04E-10	-18.22
SAUSA300_1068	-	PHENOL SOLUBLE MODULIN BETA 1	---	5.18E-12	-25.45
SAUSA300_1069	-	Hydrolase (HAD superfamily)	General function prediction only	9.36E-03	2.42
SAUSA300_1070	-	Acetyltransferase (EC 2.3.1.-)	---	3.50E-03	-2.89
SAUSA300_1071	-	Hypothetical membrane spanning protein	Function unknown	1.12E-03	3.45
SAUSA300_1073	mraW	S-adenosyl-methyltransferase mraW (EC 2.1.1.-)	Cell wall/membrane biogenesis	2.83E-04	-4.03
SAUSA300_1074	ftsL	Cell division protein ftsL	Cell cycle control, mitosis and meiosis	1.48E-03	-2.14
SAUSA300_1087	ileS	Isoleucyl-tRNA synthetase (EC 6.1.1.5)	Translation	9.87E-07	5.41
SAUSA300_1091	pyrR	Uracil phosphoribosyltransferase (EC 2.4.2.9) / Pyrimidin	Nucleotide transport and metabolism	1.75E-05	-2.50
SAUSA300_1093	pyrB	Aspartate carbamoyltransferase (EC 2.1.3.2)	Nucleotide transport and metabolism	1.37E-06	-2.83
SAUSA300_1095	carA	Carbamoyl-phosphate synthase small chain (EC 6.3.5.5)	Amino acid transport and metabolism	7.86E-10	-2.14
SAUSA300_1097	pyrF	Orotidine 5'-phosphate decarboxylase (EC 4.1.1.23)	Nucleotide transport and metabolism	5.27E-05	-13.66
SAUSA300_1098	pyrE	Orotate phosphoribosyltransferase (EC 2.4.2.10)	Nucleotide transport and metabolism	2.37E-10	-6.32
SAUSA300_1099	-	Hypothetical protein	---	8.10E-08	2.86
SAUSA300_1105	priA	Primosomal protein N'	Replication, recombination and repair	4.52E-04	-3.34
SAUSA300_1110	sun	16S rRNA m(5)C 967 methyltransferase (EC 2.1.1.-)	Translation	2.80E-06	-4.22
SAUSA300_1112	-	Protein phosphatase 2C (EC 3.1.3.16)	Signal transduction mechanisms	9.25E-06	-3.18
SAUSA300_1113	pknB	Serine/threonine protein kinase (EC 2.7.11.1)	Function unknown	3.89E-04	-3.27
SAUSA300_1117	rpmB	LSU ribosomal protein L28P	Translation	1.43E-09	-3.42
SAUSA300_1118	-	General stress protein, Gls24 family	---	2.58E-02	2.20
SAUSA300_1120	recG	ATP-dependent DNA helicase recG (EC 3.6.1.-)	Replication, recombination and repair	1.79E-02	4.52
SAUSA300_1128	ftsY	Cell division protein ftsY	Intracellular trafficking and secretion	5.11E-03	-5.98
SAUSA300_1135	-	Integral membrane protein	Function unknown	2.50E-04	2.61
SAUSA300_1138	sucC	Succinyl-CoA synthetase beta chain (EC 6.2.1.5)	Energy production and conversion	5.54E-03	-2.90
SAUSA300_1140	lytN	Cell wall hydrolase LytN	---	2.87E-05	12.38
SAUSA300_1141	-	UDP-N-acetylmuramoylheptapeptide-glycine L-seryltrans	Defense mechanisms/Virulence	2.45E-09	12.29
SAUSA300_1142	dprA	DNA processing protein	Replication, recombination and repair	2.54E-08	18.00
SAUSA300_1143	topA	DNA topoisomerase I (EC 5.99.1.2)	Replication, recombination and repair	1.75E-02	2.65
SAUSA300_1146	hslV	ATP-dependent endopeptidase hsl proteolytic subunit hslV	---	2.15E-05	-2.89
SAUSA300_1147	hslU	ATP-dependent endopeptidase hsl ATP-binding subunit hs	---	5.73E-06	-2.73
SAUSA300_1155	-	Membrane endopeptidase, M50 family	Cell wall/membrane biogenesis	4.77E-03	-2.73
SAUSA300_1156	proS	Prolyl-tRNA synthetase (EC 6.1.1.15)	Translation	2.10E-03	-2.46
SAUSA300_1157	polC	DNA polymerase III alpha subunit (EC 2.7.7.7)	Replication, recombination and repair	3.68E-11	2.30
SAUSA300_1163	rbfA	Ribosome-binding factor A	Translation	2.49E-02	2.31
SAUSA300_1164	truB	tRNA pseudouridine synthase B (EC 4.2.1.70)	Translation	1.14E-04	-3.22
SAUSA300_1165	ribF	Riboflavin kinase (EC 2.7.1.26) / FMN adenylyltransferas	Coenzyme transport and metabolism	3.40E-09	-2.09
SAUSA300_1167	pnpA	Polyribonucleotide nucleotidyltransferase (EC 2.7.7.8) / P	Translation	2.81E-03	-2.13
SAUSA300_1174	-	Protein with ACT domain	---	5.07E-05	-2.13
SAUSA300_1176	pgsA	CDP-diacylglycerol--glycerol-3-phosphate 3-phosphatidyl	Lipid transport and metabolism	3.91E-09	-8.99
SAUSA300_1182	-	2-oxoacid ferredoxin oxidoreductase, alpha subunit (EC 1.	Energy production and conversion	1.63E-05	2.01
SAUSA300_1183	-	2-oxoacid ferredoxin oxidoreductase, beta subunit (EC 1.2	Energy production and conversion	4.35E-04	5.96
SAUSA300_1184	-	Hypothetical protein	Function unknown	2.85E-03	3.71
SAUSA300_1187	-	Hydroxyethylthiazole permease	Function unknown	1.53E-03	-7.88
SAUSA300_1189	mutL	DNA mismatch repair protein mutL	Replication, recombination and repair	1.91E-04	-2.48
SAUSA300_1194	-	Lysophospholipase L2 (EC 3.1.1.5)	Lipid transport and metabolism	3.82E-03	5.99
SAUSA300_1196	hfq	RNA-binding protein, Hfq family	General function prediction only	1.43E-02	-2.09
SAUSA300_1200	glnR	Transcriptional regulator, MerR family	Transcription	1.51E-07	-3.55
SAUSA300_1201	glnA	Glutamine synthetase (EC 6.3.1.2)	Amino acid transport and metabolism	6.24E-09	-3.65
SAUSA300_1205	-	Hypothetical protein	---	1.04E-04	-4.76
SAUSA300_1206	-	Hypothetical protein	---	2.72E-03	18.84
SAUSA300_1211	-	Hypothetical protein	---	6.64E-03	5.23
SAUSA300_1217	-	ABC transporter ATP-binding protein	Defense mechanisms/Virulence	1.89E-04	22.63
SAUSA300_1218	-	ABC transporter permease protein	Defense mechanisms/Virulence	2.26E-02	21.30
SAUSA300_1222	nuc	Thermonuclease (EC 3.1.31.1)	Replication, recombination and repair	4.15E-08	14.90
SAUSA300_1225	-	Aspartokinase (EC 2.7.2.4)	Amino acid transport and metabolism	6.33E-07	2.82

probe set mapping	synonym	description	NCBI COG	p-value	day one / inoculum
SAUSA300_1230	-	Hypothetical protein	---	2.18E-03	-2.17
SAUSA300_1231	-	Lysine-specific permease	Amino acid transport and metabolism	3.33E-07	-2.18
SAUSA300_1233	rpmG	LSU ribosomal protein L33P	Translation	2.42E-03	-2.18
SAUSA300_1234	rpsN	SSU ribosomal protein S14P	Translation	2.35E-03	9.52
SAUSA300_1235	guaC	GMP reductase (EC 1.7.1.7)	Nucleotide transport and metabolism	1.03E-11	5.56
SAUSA300_1237	lexA	LexA repressor (EC 3.4.21.88)	Transcription	5.52E-07	-3.44
SAUSA300_1246	acnA	Aconitate hydratase (EC 4.2.1.3)	Energy production and conversion	2.87E-11	-2.13
SAUSA300_1247	-	Thioesterase (EC 3.1.2.-)	General function prediction only	2.24E-05	-3.04
SAUSA300_1248	-	Hypothetical cytosolic protein	Function unknown	7.14E-05	2.30
SAUSA300_1251	parC	Topoisomerase IV subunit A (EC 5.99.1.-)	Replication, recombination and repair	6.07E-05	-2.47
SAUSA300_1255	fmtC	Lysyltransferase (EC 2.3.2.3)	---	4.90E-04	-2.17
SAUSA300_1256	msrA	Peptide methionine sulfoxide reductase msrA (EC 1.8.4.11	Posttranslational modification, protein turnov	7.81E-05	2.31
SAUSA300_1257	msrR	Transcriptional regulator, LytR family	Transcription	5.29E-03	2.28
SAUSA300_1259	-	ImpB/MucB/SamB family protein	Replication, recombination and repair	6.79E-09	4.56
SAUSA300_1261	-	Deblocking aminopeptidase (EC 3.4.11.-)	Carbohydrate transport and metabolism	7.57E-03	-2.73
SAUSA300_1262	trpE	Anthranilate synthase component I (EC 4.1.3.27)	Amino acid transport and metabolism	1.25E-04	4.81
SAUSA300_1263	trpG	Anthranilate synthase component II (EC 4.1.3.27) / Para-a	Amino acid transport and metabolism	3.57E-08	5.08
SAUSA300_1264	trpD	Anthranilate phosphoribosyltransferase (EC 2.4.2.18)	Amino acid transport and metabolism	3.02E-09	5.79
SAUSA300_1265	trpC	Indole-3-glycerol phosphate synthase (EC 4.1.1.48)	Amino acid transport and metabolism	1.01E-02	8.98
SAUSA300_1266	trpF	N-(5'-phosphoribosyl)anthranilate isomerase (EC 5.3.1.24	Amino acid transport and metabolism	3.47E-13	34.62
SAUSA300_1267	trpB	Tryptophan synthase beta chain (EC 4.2.1.20)	Amino acid transport and metabolism	5.50E-12	26.98
SAUSA300_1268	trpA	Tryptophan synthase alpha chain (EC 4.2.1.20)	Amino acid transport and metabolism	1.04E-06	15.92
SAUSA300_1270	femB	UDP-N-acetylmuramoylpentapeptide-triglycine glycyltran	Defense mechanisms/Virulence	8.93E-05	-2.19
SAUSA300_1277	-	Permease	---	2.28E-07	7.28
SAUSA300_1286	-	Aspartokinase (EC 2.7.2.4)	Amino acid transport and metabolism	2.18E-14	25.20
SAUSA300_1287	asd	Aspartate-semialdehyde dehydrogenase (EC 1.2.1.11)	Amino acid transport and metabolism	5.83E-03	3.97
SAUSA300_1288	dapA	Dihydrodipicolinate synthase (EC 4.2.1.52)	Amino acid transport and metabolism	1.16E-04	4.76
SAUSA300_1289	dapB	Dihydrodipicolinate reductase (EC 1.3.1.26)	Amino acid transport and metabolism	4.56E-05	4.48
SAUSA300_1290	dapD	Tetrahydrodipicolinate N-acetyltransferase (EC 2.3.1.89)	Amino acid transport and metabolism	1.11E-06	3.73
SAUSA300_1291	-	Putative N-acetyldiaminopimelate deacetylase (EC 3.5.1.4	General function prediction only	7.23E-10	7.10
SAUSA300_1293	lysA	Diaminopimelate decarboxylase (EC 4.1.1.20)	Amino acid transport and metabolism	2.95E-05	5.33
SAUSA300_1295	-	Cold shock protein	---	1.12E-03	-3.10
SAUSA300_1300	brnQ	Branched-chain amino acid transport system carrier protei	Amino acid transport and metabolism	1.20E-11	29.57
SAUSA300_1305	sucB	Dihydrolipoamide succinyltransferase component (E2) of	Energy production and conversion	2.89E-05	-2.47
SAUSA300_1306	sucA	2-oxoglutarate dehydrogenase E1 component (EC 1.2.4.2)	Energy production and conversion	9.97E-11	-3.74
SAUSA300_1307	arlS	Two-component sensor kinase arlS (EC 2.7.3.-)	Signal transduction mechanisms	1.03E-12	-3.72
SAUSA300_1311	murG	UDP-N-acetylglucosamine--N-acetylmuramyl-(pentapepti	Cell wall/membrane biogenesis	1.14E-04	2.40
SAUSA300_1319	folA	Dihydrofolate reductase (EC 1.5.1.3)	Coenzyme transport and metabolism	1.32E-06	-2.83
SAUSA300_1320	thyA	Thymidylate synthase (EC 2.1.1.45)	Nucleotide transport and metabolism	3.02E-13	-3.93
SAUSA300_1321	-	Hypothetical protein	---	2.43E-05	-9.67
SAUSA300_1325	-	Hypothetical protein	---	3.18E-13	66.06
SAUSA300_1326	-	Ribonuclease HI (EC 3.1.26.4) / Cell wall enzyme EBSB	Replication, recombination and repair	7.19E-10	40.47
SAUSA300_1327	-	Erythrocyte membrane binding protein	Replication, recombination and repair	9.14E-10	21.04
SAUSA300_1328	-	Multidrug resistance protein B	---	1.30E-09	8.27
SAUSA300_1329	-	Amino acid permease	Amino acid transport and metabolism	8.25E-03	7.66
SAUSA300_1330	ilvA	Threonine dehydratase (EC 4.3.1.19)	Amino acid transport and metabolism	3.44E-06	5.31
SAUSA300_1331	ald	Alanine dehydrogenase (EC 1.4.1.1)	Amino acid transport and metabolism	1.40E-06	37.90
SAUSA300_1333	-	putative GTPases (dynamin-related)	General function prediction only	4.25E-04	2.81
SAUSA300_1335	-	Hypothetical protein	---	1.64E-06	4.11
SAUSA300_1342	-	Hypothetical protein	---	1.21E-03	2.61
SAUSA300_1345	asnC	Asparaginyl-tRNA synthetase (EC 6.1.1.22)	Translation	2.45E-06	-5.08
SAUSA300_1346	-	ATP-dependent helicase, DinG family	Transcription	8.59E-16	9.37
SAUSA300_1353	-	Hypothetical cytosolic protein	Function unknown	1.28E-02	-5.98
SAUSA300_1359	-	Farnesyl pyrophosphate synthetase (EC 2.5.1.1) / Geranyl	Coenzyme transport and metabolism	1.53E-07	-2.39
SAUSA300_1360	ubiE	S-adenosylmethionine:2-demethylmenaquinone methyltra	Coenzyme transport and metabolism	1.68E-06	-5.10
SAUSA300_1363	gpsA	Glycerol-3-phosphate dehydrogenase [NAD(P)+] (EC 1.1	Energy production and conversion	2.92E-04	-2.56
SAUSA300_1367	cmk	Cytidylate kinase (EC 2.7.4.14)	Nucleotide transport and metabolism	8.77E-04	-3.21
SAUSA300_1368	ansA	L-asparaginase (EC 3.5.1.1)	Amino acid transport and metabolism	4.39E-06	18.18
SAUSA300_1371	recQ	ATP-dependent DNA helicase recQ (EC 3.6.1.-)	Replication, recombination and repair	2.57E-04	-2.63
SAUSA300_1372	-	Hypothetical protein	Function unknown	1.03E-02	-2.30
SAUSA300_1375	-	Hypothetical protein	---	1.17E-02	7.60
SAUSA300_1377	-	Hypothetical protein	---	2.84E-02	13.38
SAUSA300_1378	-	Hypothetical cytosolic protein	---	7.78E-06	173.60
SAUSA300_1379	-	Hypothetical cytosolic protein	---	4.28E-07	10.33
SAUSA300_1380	-	Hypothetical protein	---	1.66E-13	23.81
SAUSA300_1382	lukS-PV	Leukocidin S subunit	---	4.94E-03	12.74
SAUSA300_1383	-	N-acetylmuramoyl-L-alanine amidase (EC 3.5.1.28)	---	1.15E-15	71.91
SAUSA300_1384	-	Holin	---	2.95E-06	81.74
SAUSA300_1385	-	Phage protein	---	1.20E-05	34.38
SAUSA300_1387	-	Phage protein	---	8.08E-07	68.50
SAUSA300_1388	-	Phage protein	---	1.93E-09	50.40
SAUSA300_1389	-	Teichoic acid biosynthesis protein C	---	3.54E-10	47.58
SAUSA300_1390	-	Hypothetical protein	---	3.81E-06	10.21
SAUSA300_1393	-	Phage protein	General function prediction only	1.33E-11	8.85
SAUSA300_1394	-	Hypothetical protein	---	4.64E-11	37.97
SAUSA300_1395	-	Hypothetical protein	---	5.56E-07	92.08
SAUSA300_1397	-	Major tail protein	---	2.83E-08	13.61
SAUSA300_1398	-	Hypothetical protein	---	3.23E-07	21.18
SAUSA300_1400	-	Phage protein	---	4.14E-06	19.39
SAUSA300_1401	-	Phage Prohead Protease	---	1.90E-08	26.17

probe set mapping	synonym	description	NCBI COG	p-value	day one / inoculum
SAUSA300_1402	-	ATP-dependent endopeptidase clp proteolytic subunit clpP	Posttranslational modification, protein turnov	8.20E-06	10.72
SAUSA300_1404	-	Terminase large subunit	General function prediction only	1.02E-13	52.14
SAUSA300_1406	-	Phage endonuclease	Defense mechanisms/Virulence	3.55E-08	20.06
SAUSA300_1408	-	Phage-related DNA helicase	Transcription	2.96E-05	4.81
SAUSA300_1410	-	Virulence-associated protein E	---	5.37E-12	37.73
SAUSA300_1411	-	Phage protein	---	6.58E-04	2.64
SAUSA300_1417	-	Hypothetical cytosolic protein	Function unknown	4.06E-08	44.23
SAUSA300_1419	-	Hypothetical protein	---	1.34E-06	49.85
SAUSA300_1420	-	Phage protein	---	1.30E-06	26.61
SAUSA300_1421	-	Phage protein	---	1.88E-11	20.87
SAUSA300_1423	polA	PHAGE-RELATED DNA POLYMERASE (EC 2.7.7.7)	Replication, recombination and repair	4.00E-09	17.50
SAUSA300_1424	-	Phage protein	---	5.73E-09	29.06
SAUSA300_1425	-	Phage protein	---	4.50E-11	23.24
SAUSA300_1426	-	Hypothetical protein	---	6.12E-08	242.90
SAUSA300_1430	-	Phage protein	---	5.57E-05	9.64
SAUSA300_1431	-	Phage protein	---	3.78E-08	202.86
SAUSA300_1433	-	Transcriptional regulator, pbsX family	Transcription	5.09E-10	78.53
SAUSA300_1442	srrA	Transcriptional regulatory protein resD	Signal transduction mechanisms	1.37E-03	-3.00
SAUSA300_1443	rluB	Ribosomal large subunit pseudouridine synthase B (EC 4.2	Translation	4.37E-09	-3.89
SAUSA300_1444	scpB	Segregation and condensation protein ScpB	Transcription	2.05E-06	-3.43
SAUSA300_1446	-	Hypothetical protein	Function unknown	4.97E-07	15.45
SAUSA300_1448	-	Ferric uptake regulation protein	Inorganic ion transport and metabolism	1.48E-02	-2.09
SAUSA300_1452	proC	Pyrroline-5-carboxylate reductase (EC 1.5.1.2)	Amino acid transport and metabolism	3.06E-02	2.64
SAUSA300_1458	-	Lactoylglutathione lyase (EC 4.4.1.5)	Amino acid transport and metabolism	1.74E-04	-4.52
SAUSA300_1463	-	Hypothetical protein	---	3.79E-05	3.03
SAUSA300_1469	argR	Arginine repressor, argR	Transcription	4.96E-04	-2.25
SAUSA300_1471	xseB	Exodeoxyribonuclease VII small subunit (EC 3.1.11.6)	Replication, recombination and repair	1.81E-02	2.33
SAUSA300_1474	-	General stress protein, Gls24 family	---	5.93E-04	-2.82
SAUSA300_1480	-	Secretory antigen precursor SsaA	---	4.51E-04	8.45
SAUSA300_1481	-	Hypothetical protein	---	5.97E-07	10.57
SAUSA300_1482	-	FtsK/SpoIIIE family	Cell cycle control, mitosis and meiosis	3.81E-05	36.51
SAUSA300_1483	-	DNA segregation ATPase and related proteins	Intracellular trafficking and secretion	9.26E-11	10.41
SAUSA300_1484	-	Hypothetical protein	---	5.99E-03	2.60
SAUSA300_1486	-	Hypothetical protein	---	7.21E-08	26.53
SAUSA300_1487	-	Transcriptional regulator, Cro/CI family	Replication, recombination and repair	4.38E-09	6.64
SAUSA300_1488	-	Hypothetical protein	---	1.83E-02	4.56
SAUSA300_1489	-	Hypothetical protein	---	8.44E-05	9.63
SAUSA300_1490	efp	Protein Translation Elongation Factor P (EF-P)	Translation	6.78E-07	-2.50
SAUSA300_1492	-	Hypothetical protein	---	1.96E-02	6.04
SAUSA300_1496	-	Glycine dehydrogenase [decarboxylating] (EC 1.4.4.2)	Amino acid transport and metabolism	2.28E-03	3.26
SAUSA300_1498	gcvT	Aminomethyltransferase (EC 2.1.2.10)	Amino acid transport and metabolism	9.63E-09	3.50
SAUSA300_1500	-	Hypothetical membrane associated protein	Intracellular trafficking and secretion	2.78E-04	40.18
SAUSA300_1501	-	ComG operon protein 4	---	1.03E-04	6.41
SAUSA300_1502	-	ComG operon protein 3	---	1.90E-05	4.38
SAUSA300_1503	-	ComG operon protein 2	---	3.06E-03	22.61
SAUSA300_1504	-	ComG operon protein 1	---	2.85E-04	9.04
SAUSA300_1506	-	Hypothetical cytosolic protein	Function unknown	9.08E-06	-6.63
SAUSA300_1507	glk	Glucokinase (EC 2.7.1.2)	Transcription	1.65E-13	-18.82
SAUSA300_1508	-	Hypothetical cytosolic protein	Function unknown	5.82E-11	-10.20
SAUSA300_1512	pbp3	Penicillin binding protein transpeptidase	Cell wall/membrane biogenesis	5.23E-07	2.99
SAUSA300_1513	-	Superoxide dismutase (EC 1.15.1.1)	Inorganic ion transport and metabolism	3.09E-06	-2.59
SAUSA300_1519	-	NIF3-related protein	Function unknown	2.86E-03	2.39
SAUSA300_1520	-	Hypothetical cytosolic protein	General function prediction only	5.34E-09	-2.27
SAUSA300_1521	rpoD	RNA polymerase sigma factor rpoD	Transcription	2.20E-07	-2.35
SAUSA300_1522	dnaG	DNA primase (EC 2.7.7.-)	Replication, recombination and repair	2.92E-06	-13.52
SAUSA300_1525	glyS	Glycyl-tRNA synthetase (EC 6.1.1.14)	Translation	1.27E-14	-3.93
SAUSA300_1526	recO	DNA repair protein recO	Replication, recombination and repair	2.76E-06	-2.73
SAUSA300_1529	dgkA	Diacylglycerol kinase (EC 2.7.1.107)	Cell wall/membrane biogenesis	5.18E-07	-3.18
SAUSA300_1530	-	Hypothetical metal-binding protein	General function prediction only	2.27E-05	-9.72
SAUSA300_1531	phoH	PhoH protein	Signal transduction mechanisms	7.18E-08	-11.08
SAUSA300_1534	-	Transporter	Posttranslational modification, protein turnov	2.60E-03	-2.49
SAUSA300_1535	rpsU	SSU ribosomal protein S21P	Translation	1.30E-03	-2.51
SAUSA300_1538	prmA	Ribosomal protein L11 methyltransferase (EC 2.1.1.-)	Translation	2.58E-05	7.21
SAUSA300_1539	dnaJ	Chaperone protein dnaJ	---	1.18E-09	4.81
SAUSA300_1540	dnaK	Chaperone protein dnaK	---	6.91E-05	18.07
SAUSA300_1541	grpE	GrpE protein	---	8.42E-04	15.90
SAUSA300_1542	hrcA	Heat-inducible transcription repressor hrcA	---	1.25E-07	32.28
SAUSA300_1545	rpsT	SSU ribosomal protein S20P	Translation	2.18E-06	-2.10
SAUSA300_1546	holA	DNA polymerase III, delta subunit (EC 2.7.7.7)	Replication, recombination and repair	2.18E-03	9.50
SAUSA300_1547	-	COME operon protein 3	---	4.92E-03	6.33
SAUSA300_1549	-	COME operon protein 1	Replication, recombination and repair	7.01E-08	30.27
SAUSA300_1550	-	Methyltransferase (EC 2.1.1.-)	---	9.27E-05	-2.96
SAUSA300_1554	-	Hypothetical RNA binding protein	Translation	9.93E-05	-3.26
SAUSA300_1558	mtnN	5'-methylthioadenosine nucleosidase (EC 3.2.2.16) / S-ade	Nucleotide transport and metabolism	7.00E-03	5.69
SAUSA300_1559	-	Enterotoxin	---	2.31E-05	3.43
SAUSA300_1560	-	Fic family protein	Function unknown	1.33E-09	13.98
SAUSA300_1561	-	Hypothetical membrane spanning protein	Inorganic ion transport and metabolism	9.10E-10	-2.78
SAUSA300_1564	accB	Biotin carboxyl carrier protein of acetyl-CoA carboxylase	Lipid transport and metabolism	3.76E-06	-3.03
SAUSA300_1570	-	Peptidase family U32	Posttranslational modification, protein turnov	3.01E-05	-3.54
SAUSA300_1572	-	Hypothetical cytosolic protein	Function unknown	4.17E-05	-2.39

probe set mapping	synonym	description	NCBI COG	p-value	day one / inoculum
SAUSA300_1573	-	Endonuclease involved in recombination	Replication, recombination and repair	1.18E-04	-2.48
SAUSA300_1584	-	ATPase, AAA family	Replication, recombination and repair	2.27E-12	-8.88
SAUSA300_1585	-	ThiF/MoeB family protein	Coenzyme transport and metabolism	7.45E-03	3.40
SAUSA300_1589	dtd	D-tyrosyl-tRNA(Tyr) deacylase (EC 3.1.-.-)	Translation	3.58E-05	-2.52
SAUSA300_1591	apt	Adenine phosphoribosyltransferase (EC 2.4.2.7)	Nucleotide transport and metabolism	2.97E-04	-3.30
SAUSA300_1592	recJ	Single-stranded-DNA-specific exonuclease recJ (EC 3.1.-.	Replication, recombination and repair	3.41E-06	6.79
SAUSA300_1595	tgt	Queuine tRNA-ribosyltransferase (EC 2.4.2.29)	Translation	7.51E-04	-3.04
SAUSA300_1596	queA	S-adenosylmethionine:tRNA ribosyltransferase-isomerase	Translation	1.39E-06	-4.07
SAUSA300_1597	ruvB	Holliday junction DNA helicase ruvB	Replication, recombination and repair	1.49E-06	-2.15
SAUSA300_1598	ruvA	Holliday junction DNA helicase ruvA	Replication, recombination and repair	3.83E-10	-2.32
SAUSA300_1606	-	Hypothetical protein	---	7.76E-06	20.47
SAUSA300_1610	folC	Folylpolyglutamate synthase (EC 6.3.2.17) / Dihydrofolat	Coenzyme transport and metabolism	3.92E-07	-2.63
SAUSA300_1615	hemB	Delta-aminolevulinic acid dehydratase (EC 4.2.1.24)	Coenzyme transport and metabolism	5.30E-03	2.03
SAUSA300_1617	hemC	Porphobilinogen deaminase (EC 2.5.1.61)	Coenzyme transport and metabolism	3.82E-08	-7.27
SAUSA300_1618	hemX	Putative heme export permease	Posttranslational modification, protein turno	3.05E-06	-6.16
SAUSA300_1620	engB	GTP-binding protein YihA	General function prediction only	1.18E-02	6.55
SAUSA300_1625	rplT	LSU ribosomal protein L20P	Translation	1.57E-05	-3.68
SAUSA300_1627	infC	Bacterial Protein Translation Initiation Factor 3 (IF-3)	Translation	8.68E-08	-2.35
SAUSA300_1628	lysP	Lysine-specific permease	Amino acid transport and metabolism	1.48E-06	2.19
SAUSA300_1630	dnaI	Primosomal protein dnaI	Replication, recombination and repair	4.82E-09	-2.52
SAUSA300_1633	gap	Glyceraldehyde 3-phosphate dehydrogenase (EC 1.2.1.12)	Carbohydrate transport and metabolism	6.17E-07	-3.28
SAUSA300_1637	-	Glucosyltransferase (side chain biosynthesis) (EC 2.4.1.-)	---	2.72E-05	5.51
SAUSA300_1638	phoR	Phosphate regulon sensor protein phoR (EC 2.7.3.-)	Signal transduction mechanisms	1.34E-09	-3.98
SAUSA300_1639	phoP	Alkaline phosphatase synthesis two-component response r	Signal transduction mechanisms	5.20E-07	-4.43
SAUSA300_1650	-	Phosphoesterase, DHH family protein	General function prediction only	1.77E-09	-3.43
SAUSA300_1651	-	Cytosolic protein containing multiple CBS domains	Transcription	1.05E-05	-2.19
SAUSA300_1652	-	Putative transcription factors	Signal transduction mechanisms	6.15E-05	-2.79
SAUSA300_1653	-	Metal-dependent hydrolase (EC 3.-.-.-)	General function prediction only	1.05E-06	-2.69
SAUSA300_1654	-	Xaa-Pro aminopeptidase (EC 3.4.11.9)	Amino acid transport and metabolism	6.17E-08	2.01
SAUSA300_1655	ald	Alanine dehydrogenase (EC 1.4.1.1)	Amino acid transport and metabolism	1.36E-06	-3.29
SAUSA300_1657	ackA	Acetate kinase (EC 2.7.2.1)	Energy production and conversion	8.18E-03	-2.01
SAUSA300_1661	thiI	Thiamine biosynthesis protein thiI	Coenzyme transport and metabolism	8.04E-05	4.37
SAUSA300_1662	-	Cysteine desulfurase (EC 2.8.1.7) / Selenocysteine lyase (	Amino acid transport and metabolism	2.80E-06	5.57
SAUSA300_1664	ezrA	Septation ring formation regulator	Cell cycle control, mitosis and meiosis	6.35E-06	-2.39
SAUSA300_1667	-	Glycerophosphoryl diester phosphodiesterase (EC 3.1.4.4	Energy production and conversion	9.15E-05	7.35
SAUSA300_1670	serA	D-3-phosphoglycerate dehydrogenase (EC 1.1.1.95)	Coenzyme transport and metabolism	5.29E-05	7.05
SAUSA300_1671	-	HPR(SER) KINASE (EC 2.7.1.-) / PHOSPHATASE (EC	General function prediction only	6.42E-05	3.17
SAUSA300_1673	-	1-acyl-sn-glycerol-3-phosphate acyltransferase (EC 2.3.1.	---	4.17E-03	7.07
SAUSA300_1674	-	Endopeptidase degP (EC 3.4.21.-)	Posttranslational modification, protein turno	1.57E-02	6.46
SAUSA300_1675	tyrS	Tyrosyl-tRNA synthetase (EC 6.1.1.1)	Translation	1.04E-03	6.36
SAUSA300_1677	-	Fibronectin-binding protein	---	2.63E-03	-3.35
SAUSA300_1679	acsA	Acetyl-coenzyme A synthetase (EC 6.2.1.1)	Lipid transport and metabolism	5.07E-06	-9.80
SAUSA300_1680	acuA	Acetoin utilization protein acuA (EC 2.3.1.-)	---	8.50E-05	2.59
SAUSA300_1684	-	Hypothetical exported protein	---	3.29E-07	-3.72
SAUSA300_1685	-	General stress protein	---	9.13E-12	-4.64
SAUSA300_1687	-	Cell division protein ftsK	Cell cycle control, mitosis and meiosis	1.16E-04	-7.38
SAUSA300_1688	-	tRNA binding domain protein	General function prediction only	9.90E-06	-2.15
SAUSA300_1692	-	Hypothetical protein	Function unknown	6.36E-07	-3.27
SAUSA300_1693	-	Metal-dependent hydrolase (EC 3.-.-.-)	General function prediction only	4.56E-08	2.12
SAUSA300_1698	-	Hypothetical protein	---	5.33E-12	-4.67
SAUSA300_1702	-	Extracellular matrix binding protein	---	5.80E-10	10.98
SAUSA300_1703	-	Rhodanese-related sulfurtransferases	Inorganic ion transport and metabolism	1.13E-02	3.55
SAUSA300_1704	leuS	Leucyl-tRNA synthetase (EC 6.1.1.4)	Translation	2.58E-04	5.37
SAUSA300_1711	putA	Proline dehydrogenase (EC 1.5.99.8)	Amino acid transport and metabolism	3.00E-05	-9.18
SAUSA300_1712	ribH	6,7-dimethyl-8-ribityllumazine synthase (EC 2.5.1.9)	Coenzyme transport and metabolism	5.09E-03	5.92
SAUSA300_1715	ribD	Diaminohydroxyphosphoribosylaminopyrimidine deamina	Coenzyme transport and metabolism	2.58E-03	3.95
SAUSA300_1722	-	Hypothetical protein	Transcription	7.80E-05	5.64
SAUSA300_1731	pckA	Phosphoenolpyruvate carboxykinase [ATP] (EC 4.1.1.49)	Energy production and conversion	2.40E-09	-2.50
SAUSA300_1736	-	Hypothetical cytosolic protein	Function unknown	8.98E-03	4.72
SAUSA300_1738	-	Hypothetical protein	---	1.35E-10	4.37
SAUSA300_1739	-	Endonuclease (EC 3.1.-.-)	---	2.80E-11	-7.69
SAUSA300_1740	-	Hypothetical protein	---	3.54E-14	-7.58
SAUSA300_1741	-	Hypothetical protein	---	3.84E-05	2.42
SAUSA300_1747	-	Hypothetical protein	---	1.96E-04	5.14
SAUSA300_1750	-	Hypothetical protein	---	1.62E-04	5.29
SAUSA300_1751	hsdS	Type I restriction-modification system specificity subunit	Defense mechanisms/Virulence	3.02E-03	2.24
SAUSA300_1753	splF	Serine protease (EC 3.4.21.-)	---	1.10E-03	4.40
SAUSA300_1754	splE	Serine protease (EC 3.4.21.-)	---	1.48E-02	10.81
SAUSA300_1755	splD	Serine protease (EC 3.4.21.-)	---	5.42E-03	7.03
SAUSA300_1756	splC	Serine protease (EC 3.4.21.-)	---	2.53E-03	8.10
SAUSA300_1757	splB	Serine protease (EC 3.4.21.-)	---	3.06E-03	17.60
SAUSA300_1760	epiG	EPIDERMIN RESISTANCE TRANSMEMBRANE PRO	---	2.17E-12	-22.86
SAUSA300_1761	epiE	Lantibiotic transport permease protein	---	2.09E-13	-9.62
SAUSA300_1762	epiF	Lantibiotic transport ATP-binding protein	Defense mechanisms/Virulence	7.10E-11	-6.28
SAUSA300_1763	epiP	Epidermin leader peptide processing serine protease EPIP	Posttranslational modification, protein turno	2.52E-03	5.64
SAUSA300_1765	epiC	Lanthionine synthetase (lantibiotic biosynthesis)	---	9.37E-05	9.19
SAUSA300_1766	epiB	Serine (threonine) dehydratase (lantibiotic biosynthesis)	---	8.10E-06	2.52
SAUSA300_1767	epiA	Lantibiotic gallidermin	---	4.74E-08	-3.22
SAUSA300_1768	lukD	Hypothetical protein	---	4.01E-07	3.58
SAUSA300_1769	lukE	Leukocidin S subunit	---	3.21E-08	25.10

probe set mapping	synonym	description	NCBI COG	p-value	day one / inoculum
SAUSA300_1771	-	Hypothetical protein	---	1.90E-07	-2.18
SAUSA300_1783	hemE	Uroporphyrinogen decarboxylase (EC 4.1.1.37)	Coenzyme transport and metabolism	3.20E-02	2.24
SAUSA300_1788	-	Hypothetical protein	General function prediction only	6.08E-12	-4.81
SAUSA300_1789	-	Hypothetical protein	---	2.55E-06	-3.33
SAUSA300_1790	prsA	Peptidyl-prolyl cis-trans isomerase (EC 5.2.1.8)	Posttranslational modification, protein turnov	2.36E-08	24.28
SAUSA300_1792	-	Hypothetical membrane associated protein	Function unknown	6.50E-05	-5.76
SAUSA300_1794	-	Hypothetical protein	---	6.21E-05	28.09
SAUSA300_1795	-	Hypothetical cytosolic protein	Function unknown	1.94E-03	-2.38
SAUSA300_1798	-	Hypothetical protein	Signal transduction mechanisms	2.49E-05	-2.26
SAUSA300_1804	-	Hypothetical protein	Carbohydrate transport and metabolism	9.25E-11	-3.78
SAUSA300_1807	-	Arginine transport ATP-binding protein artP	Amino acid transport and metabolism	2.70E-06	3.34
SAUSA300_1808	-	Arginine-binding protein / Arginine transport system perm	Amino acid transport and metabolism	3.37E-11	35.33
SAUSA300_1810	-	Transposase	Replication, recombination and repair	5.03E-11	7.28
SAUSA300_1851	-	Hypothetical protein	---	5.70E-08	-16.76
SAUSA300_1856	-	PROTEASE I (EC 3.4.-.-)	General function prediction only	7.63E-07	-3.34
SAUSA300_1858	-	Thioredoxin-like oxidoreductases	General function prediction only	2.96E-03	3.53
SAUSA300_1861	-	Hypothetical protein	Function unknown	1.86E-02	-4.74
SAUSA300_1862	-	Protein tyrosine phosphatase (EC 3.1.3.48)	Signal transduction mechanisms	9.60E-12	-2.99
SAUSA300_1863	-	Hypothetical protein	---	2.00E-11	-2.51
SAUSA300_1864	-	Ribonuclease BN (EC 3.1.-.-)	Function unknown	9.11E-12	-26.61
SAUSA300_1866	vraS	Two-component sensor protein yvqE (EC 2.7.3.-)	Signal transduction mechanisms	1.31E-02	8.37
SAUSA300_1867	-	Transporter yvqF	Function unknown	3.02E-04	7.80
SAUSA300_1868	-	Hypothetical protein	---	3.51E-02	5.97
SAUSA300_1869	map	Methionine aminopeptidase (EC 3.4.11.18)	Translation	8.34E-05	-2.24
SAUSA300_1873	-	UDP-N-acetylmuramoylalanyl-D-glutamate--2,6-diamino	---	5.75E-04	-7.79
SAUSA300_1874	-	Ferritin	Inorganic ion transport and metabolism	1.66E-03	-2.27
SAUSA300_1876	-	DNA polymerase IV (EC 2.7.7.7)	Replication, recombination and repair	9.28E-04	7.18
SAUSA300_1877	-	Permease	---	2.04E-02	11.49
SAUSA300_1881	gatA	Aspartyl/glutamyl-tRNA(Asn/Gln) amidotransferase subu	Translation	3.21E-02	-6.40
SAUSA300_1887	pcrB	GERANYLGERANYLGLYCERYL DIPHOSPHATE SY	General function prediction only	1.70E-03	2.47
SAUSA300_1890	-	Staphopain (EC 3.4.22.-)	---	9.11E-14	62.49
SAUSA300_1894	-	Nicotinate phosphoribosyltransferase (EC 2.4.2.11)	Coenzyme transport and metabolism	1.09E-04	3.82
SAUSA300_1896	pheA	Prephenate dehydratase (EC 4.2.1.51)	Amino acid transport and metabolism	2.24E-06	-2.28
SAUSA300_1897	-	Transporter, Divalent Anion:Sodium Symporter family	Inorganic ion transport and metabolism	6.62E-05	-2.23
SAUSA300_1906	-	Hypothetical protein	---	2.58E-07	-2.74
SAUSA300_1908	-	Hypothetical protein	---	9.90E-06	-2.89
SAUSA300_1910	-	Hypothetical protein	---	4.32E-11	-2.07
SAUSA300_1915	-	Hypothetical protein	---	3.40E-05	-6.11
SAUSA300_1920	chs	Hypothetical protein	---	1.39E-13	34.49
SAUSA300_1923	-	CHAP domain containing protein	---	5.75E-09	11.44
SAUSA300_1924	-	Holin	---	2.84E-07	16.67
SAUSA300_1925	-	Phage protein	---	3.31E-06	6.18
SAUSA300_1926	-	Hypothetical protein	---	1.63E-02	2.14
SAUSA300_1927	-	Hypothetical protein	---	3.39E-02	35.01
SAUSA300_1928	-	Phage-related protein	---	2.35E-05	8.18
SAUSA300_1929	-	Phage-related protein	---	1.99E-07	26.90
SAUSA300_1930	-	Hypothetical protein	Function unknown	1.99E-09	6.02
SAUSA300_1941	-	Terminase large subunit	General function prediction only	1.01E-06	5.66
SAUSA300_1942	-	Phage-related protein	---	1.75E-07	14.07
SAUSA300_1943	-	HNH endonuclease family protein	Defense mechanisms/Virulence	7.91E-03	19.93
SAUSA300_1944	-	Hypothetical protein	---	2.72E-02	7.75
SAUSA300_1946	-	Transcriptional activator rinB	---	2.20E-09	3.22
SAUSA300_1949	dut	Deoxyuridine 5'-triphosphate nucleotidohydrolase (EC 3.6	Nucleotide transport and metabolism	1.11E-05	3.19
SAUSA300_1951	-	Phage protein	---	3.02E-04	6.65
SAUSA300_1952	-	Hypothetical protein	---	3.90E-03	4.71
SAUSA300_1954	-	Phage protein	---	1.63E-02	5.27
SAUSA300_1963	-	Hypothetical cytosolic protein	---	2.00E-02	2.03
SAUSA300_1966	-	Phage antirepressor protein	Function unknown	1.30E-03	-3.23
SAUSA300_1967	-	Phage protein	---	2.10E-04	2.78
SAUSA300_1969	-	Phage transcriptional repressor	Transcription	4.10E-03	2.99
SAUSA300_1971	-	Phage protein	---	6.72E-05	2.40
SAUSA300_1972	int	DNA integration/recombination/inversion protein	Replication, recombination and repair	1.25E-08	19.46
SAUSA300_1973	-	Sphingomyelin phosphodiesterase (EC 3.1.4.12)	General function prediction only	3.25E-07	40.16
SAUSA300_1976	-	Succinyl-diaminopimelate desuccinylase (EC 3.5.1.18)	Amino acid transport and metabolism	3.32E-06	-8.82
SAUSA300_1977	-	Tetracenomycin polyketide synthesis O-methyltransferase	Secondary metabolites biosynthesis, transpor	5.49E-11	67.76
SAUSA300_1978	-	Ferrichrome-binding protein / Ferrioxamine B binding pro	Inorganic ion transport and metabolism	7.56E-13	86.08
SAUSA300_1979	-	Potassium uptake protein ktrB	Inorganic ion transport and metabolism	1.81E-12	7.68
SAUSA300_1980	-	Acetyltransferase (EC 2.3.1.-)	---	2.01E-06	-3.18
SAUSA300_1981	-	Phage Terminase Small Subunit	Replication, recombination and repair	1.85E-09	47.75
SAUSA300_1982	groEL	60 kDa chaperonin GROEL	Posttranslational modification, protein turnov	7.80E-07	9.96
SAUSA300_1983	groES	10 kDa chaperonin GROES	Posttranslational modification, protein turnov	1.31E-05	19.54
SAUSA300_1987	-	Beta-ureidopropionase (EC 3.5.1.6)	General function prediction only	1.33E-07	-9.59
SAUSA300_1989	agrB	Accessory gene regulator protein B AGRB	Posttranslational modification, protein turnov	6.10E-10	-8.57
SAUSA300_1991	agrC	Sensory transduction histidine kinase AgrC (EC 2.7.3.-)	Signal transduction mechanisms	2.98E-09	-9.91
SAUSA300_1992	agrA	Accessory gene regulator protein A AGRA	---	8.83E-11	-19.77
SAUSA300_1993	-	Fructokinase (EC 2.7.1.4)	Carbohydrate transport and metabolism	3.07E-02	3.35
SAUSA300_1996	amt	Hypothetical protein	Inorganic ion transport and metabolism	1.51E-07	10.21
SAUSA300_1998	-	putative transport system permease protein	General function prediction only	5.35E-07	2.51
SAUSA300_2001	-	DNA mismatch repair protein mutS	Replication, recombination and repair	2.47E-05	14.67
SAUSA300_2004	-	Non-proteolytic protein, peptidase family M22	Posttranslational modification, protein turnov	9.82E-04	3.67

probe set mapping	synonym	description	NCBI COG	p-value	day one / inoculum
SAUSA300_2007	ilvB	Acetolactate synthase large subunit (EC 2.2.1.6)	Amino acid transport and metabolism	2.43E-05	2.21
SAUSA300_2009	ilvC	Ketol-acid reductoisomerase (EC 1.1.1.86) / 2-dehydropar	Amino acid transport and metabolism	1.15E-04	5.81
SAUSA300_2010	leuA	2-isopropylmalate synthase (EC 2.3.3.13)	Amino acid transport and metabolism	1.06E-04	3.98
SAUSA300_2011	leuB	3-isopropylmalate dehydrogenase (EC 1.1.1.85)	Energy production and conversion	5.16E-04	3.58
SAUSA300_2012	leuC	3-isopropylmalate dehydratase large subunit (EC 4.2.1.33)	Amino acid transport and metabolism	7.98E-09	8.47
SAUSA300_2013	leuD	3-isopropylmalate dehydratase small subunit (EC 4.2.1.33	Amino acid transport and metabolism	3.52E-11	19.47
SAUSA300_2014	ilvA	Threonine dehydratase (EC 4.3.1.19)	Amino acid transport and metabolism	1.03E-04	2.24
SAUSA300_2020	-	Metallopeptidase, SprT family (EC 3.4.24.-)	Function unknown	1.36E-04	-5.71
SAUSA300_2022	rpoF	RNA polymerase sigma-B factor	---	4.09E-06	-4.47
SAUSA300_2023	rsbW	Anti-sigma B factor	Signal transduction mechanisms	3.61E-09	-2.18
SAUSA300_2024	rsbV	Anti-sigma B factor antagonist	Signal transduction mechanisms	3.89E-08	-2.45
SAUSA300_2032	kdpC	Potassium-transporting ATPase C chain (EC 3.6.3.12)	Inorganic ion transport and metabolism	1.57E-09	16.22
SAUSA300_2033	kdpB	Potassium-transporting ATPase B chain (EC 3.6.3.12)	Inorganic ion transport and metabolism	4.92E-11	15.60
SAUSA300_2034	kdpA	Potassium-transporting ATPase A chain (EC 3.6.3.12)	Inorganic ion transport and metabolism	4.47E-05	22.16
SAUSA300_2035	kdpD	Sensor protein kdpD (EC 2.7.3.-)	Signal transduction mechanisms	2.42E-03	6.93
SAUSA300_2045	-	Metal dependent hydrolase	General function prediction only	2.80E-10	-5.17
SAUSA300_2048	thiM	Hydroxyethylthiazole kinase (EC 2.7.1.50)	Coenzyme transport and metabolism	5.07E-09	30.65
SAUSA300_2049	thiD	Phosphomethylpyrimidine kinase (EC 2.7.4.7) / Hydroxyn	Coenzyme transport and metabolism	3.35E-12	40.50
SAUSA300_2050	-	Transcriptional activator tenA	Transcription	2.47E-06	5.56
SAUSA300_2051	-	SCED PRECURSOR	---	6.23E-11	17.92
SAUSA300_2052	-	Single-strand DNA binding protein	Replication, recombination and repair	1.40E-02	-2.26
SAUSA300_2054	fabZ	(3R)-hydroxyacyl-[acyl carrier protein] dehydratase (EC 4	Lipid transport and metabolism	2.38E-15	-2.63
SAUSA300_2055	murA	UDP-N-acetylglucosamine 1-carboxyvinyltransferase (EC	Cell wall/membrane biogenesis	1.81E-07	-2.56
SAUSA300_2056	-	Hypothetical membrane associated protein	Function unknown	2.46E-09	-8.25
SAUSA300_2058	atpD	ATP synthase beta chain (EC 3.6.3.14)	Energy production and conversion	6.96E-08	-7.99
SAUSA300_2059	atpG	ATP synthase gamma chain (EC 3.6.3.14)	Energy production and conversion	4.90E-05	-2.79
SAUSA300_2060	atpA	ATP synthase alpha chain (EC 3.6.3.14)	Energy production and conversion	7.32E-08	-2.31
SAUSA300_2064	atpB	ATP synthase A chain (EC 3.6.3.14)	Energy production and conversion	6.94E-04	-2.55
SAUSA300_2065	-	UDP-N-acetylglucosamine 2-epimerase (EC 5.1.3.14)	Cell wall/membrane biogenesis	4.09E-06	-2.63
SAUSA300_2069	-	Protein tyrosine phosphatase (EC 3.1.3.48)	Signal transduction mechanisms	1.43E-03	2.33
SAUSA300_2072	prfA	Bacterial Peptide Chain Release Factor 1 (RF-1)	Translation	7.79E-09	-3.35
SAUSA300_2075	rho	Transcription termination factor rho	Transcription	4.79E-06	-3.04
SAUSA300_2076	-	Aldehyde dehydrogenase (EC 1.2.1.3)	Energy production and conversion	4.89E-11	-2.15
SAUSA300_2077	-	Transcriptional regulator, MarR family	Transcription	6.29E-04	-2.76
SAUSA300_2080	-	Hypothetical protein	---	2.53E-04	3.49
SAUSA300_2087	-	Peptidase family M20/M25/M40	General function prediction only	2.44E-06	5.83
SAUSA300_2095	-	Hypothetical protein	---	9.15E-09	-5.04
SAUSA300_2096	manA	Mannose-6-phosphate isomerase (EC 5.3.1.8)	Carbohydrate transport and metabolism	1.67E-06	-2.01
SAUSA300_2097	-	Putative NAD-dependent dehydrogenase	Cell wall/membrane biogenesis	2.61E-11	-4.15
SAUSA300_2104	glmS	Glucosamine--fructose-6-phosphate aminotransferase [iso	Cell wall/membrane biogenesis	2.52E-04	-2.77
SAUSA300_2105	mtlF	PTS system, mannitol-specific IIBC component (EC 2.7.1	Carbohydrate transport and metabolism	2.34E-04	3.00
SAUSA300_2106	-	Transcription antiterminator, BglG family / PTS system, m	Transcription	6.62E-05	-2.48
SAUSA300_2107	mtlA	PTS system, mannitol-specific IIA component (EC 2.7.1.6	Carbohydrate transport and metabolism	3.10E-15	-55.06
SAUSA300_2108	mtlD	Mannitol-1-phosphate 5-dehydrogenase (EC 1.1.1.17)	Carbohydrate transport and metabolism	5.89E-09	-2.43
SAUSA300_2109	fmtB	Hypothetical protein	---	1.61E-09	42.52
SAUSA300_2110	fmtB	Methicillin resistance protein	---	1.07E-11	38.24
SAUSA300_2112	-	Hypothetical membrane associated protein	Function unknown	7.92E-09	-2.00
SAUSA300_2114	rocF	Arginase (EC 3.5.3.1)	Amino acid transport and metabolism	2.64E-04	-2.16
SAUSA300_2125	-	Iron-sulfur cluster assembly/repair protein ApbC	Cell cycle control, mitosis and meiosis	1.61E-03	4.42
SAUSA300_2127	-	Hypothetical protein	---	2.72E-04	-2.41
SAUSA300_2129	-	Conserved membrane protein (hemolysin III homolog)	General function prediction only	4.66E-06	-4.37
SAUSA300_2130	-	UDP-N-acetylglucosamine pyrophosphorylase (EC 2.7.7.2	Carbohydrate transport and metabolism	6.42E-06	-3.76
SAUSA300_2131	-	Predicted membrane-bound metal-dependent hydrolase	General function prediction only	7.67E-07	-15.41
SAUSA300_2133	-	Hypothetical membrane spanning protein	Function unknown	2.81E-11	10.41
SAUSA300_2134	-	Iron(III) dicitrate transport system permease protein fecD	Inorganic ion transport and metabolism	1.70E-07	5.58
SAUSA300_2135	-	Ferrichrome transport system permease protein fhuB	Inorganic ion transport and metabolism	1.00E-11	27.36
SAUSA300_2136	-	Iron(III) dicitrate-binding protein	Inorganic ion transport and metabolism	2.63E-03	12.57
SAUSA300_2139	-	Macrolide-efflux protein	---	4.26E-06	9.15
SAUSA300_2145	-	Glycine betaine transporter	Cell wall/membrane biogenesis	1.59E-09	-4.19
SAUSA300_2146	-	Hypothetical protein	Energy production and conversion	1.58E-04	6.11
SAUSA300_2149	lacG	6-phospho-beta-galactosidase (EC 3.2.1.85)	Carbohydrate transport and metabolism	1.68E-12	5.32
SAUSA300_2150	lacE	PTS system, lactose-specific IIBC component (EC 2.7.1.6	Carbohydrate transport and metabolism	2.09E-11	54.02
SAUSA300_2151	lacF	PTS system, lactose-specific IIA component (EC 2.7.1.69)	Carbohydrate transport and metabolism	1.18E-02	3.14
SAUSA300_2152	lacD	Tagatose-bisphosphate aldolase (EC 4.1.2.40)	Carbohydrate transport and metabolism	1.69E-06	47.72
SAUSA300_2153	lacC	Tagatose-6-phosphate kinase (EC 2.7.1.144)	Carbohydrate transport and metabolism	1.59E-13	25.72
SAUSA300_2154	lacB	Galactose-6-phosphate isomerase lacB subunit (EC 5.3.1.2	Carbohydrate transport and metabolism	2.25E-08	16.72
SAUSA300_2155	lacA	Galactose-6-phosphate isomerase lacA subunit (EC 5.3.1.2	Carbohydrate transport and metabolism	1.56E-10	4.92
SAUSA300_2157	-	SIR2 family protein	Transcription	6.04E-03	5.04
SAUSA300_2159	-	Morphine 6-dehydrogenase (EC 1.1.1.218)	General function prediction only	3.56E-03	2.36
SAUSA300_2161	hysA	Hyaluronate lyase precursor (EC 4.2.2.1)	---	1.15E-10	4.85
SAUSA300_2162	-	Peptidoglycan-specific endopeptidase, M23 family	---	2.59E-06	2.17
SAUSA300_2163	-	Hydrolase (HAD superfamily)	General function prediction only	6.99E-04	2.67
SAUSA300_2164	-	Outer membrane protein	---	2.80E-13	-12.38
SAUSA300_2166	alsS	Acetolactate synthase (EC 2.2.1.6)	Amino acid transport and metabolism	1.76E-03	5.95
SAUSA300_2172	rplM	LSU ribosomal protein L13P	Translation	2.90E-03	-2.20
SAUSA300_2174	-	Cobalt transport protein cbiQ	Inorganic ion transport and metabolism	7.67E-04	-3.34
SAUSA300_2186	rpmD	LSU ribosomal protein L30P	Translation	5.22E-03	3.05
SAUSA300_2191	rpsN	SSU ribosomal protein S14P	Translation	6.92E-04	2.01
SAUSA300_2199	rplV	LSU ribosomal protein L22P	Translation	1.67E-06	-5.46
SAUSA300_2202	rplW	LSU ribosomal protein L23P	Translation	6.64E-10	-2.39

probe set mapping	synonym	description	NCBI COG	p-value	day one / inoculum
SAUSA300_2211	-	Permease	General function prediction only	3.08E-08	10.62
SAUSA300_2213	-	Acriflavin resistance plasma membrane protein	Defense mechanisms/Virulence	7.88E-05	2.72
SAUSA300_2214	-	UDP-N-acetylmuramoylpentapeptide-lysine N(6)-glycyltr	Defense mechanisms/Virulence	3.88E-04	-8.76
SAUSA300_2215	-	Hypothetical cytosolic protein	---	2.46E-04	2.22
SAUSA300_2216	-	Transcriptional regulator, MarR family	Transcription	1.85E-04	10.45
SAUSA300_2218	-	STAPHYLOCOCCAL ACCESSORY REGULATOR A	---	4.04E-08	6.38
SAUSA300_2219	moaA	Molybdenum cofactor biosynthesis protein A	Coenzyme transport and metabolism	2.57E-04	-5.16
SAUSA300_2225	moaC	Molybdenum cofactor biosynthesis protein C	Coenzyme transport and metabolism	1.22E-06	-3.36
SAUSA300_2227	moeB	Molybdopterin biosynthesis MoeB protein	Coenzyme transport and metabolism	4.01E-03	-2.02
SAUSA300_2234	-	Inosine-uridine preferring nucleoside hydrolase (EC 3.2.2.	Nucleotide transport and metabolism	1.35E-03	18.49
SAUSA300_2235	-	Ferrichrome-binding protein / Ferrioxamine B binding pro	Inorganic ion transport and metabolism	9.98E-10	6.31
SAUSA300_2237	-	UREA TRANSPORTER	Amino acid transport and metabolism	1.65E-03	4.04
SAUSA300_2239	ureB	Urease beta subunit (EC 3.5.1.5)	Amino acid transport and metabolism	1.04E-03	24.03
SAUSA300_2242	ureF	Urease accessory protein ureF	Posttranslational modification, protein turnov	1.02E-03	8.30
SAUSA300_2249	ssaA	Secretory antigen precursor SsaA	---	1.28E-02	2.66
SAUSA300_2250	nhaC	Na+/H+ antiporter nhaC	Energy production and conversion	5.98E-07	3.25
SAUSA300_2254	-	Glyoxylate reductase (NADP+) (EC 1.1.1.79) / Glyoxylate	Energy production and conversion	1.48E-08	-4.81
SAUSA300_2255	-	Salicylate hydroxylase (EC 1.14.13.1)	Coenzyme transport and metabolism	3.29E-03	3.51
SAUSA300_2257	-	Hypothetical protein	Function unknown	3.37E-14	-19.05
SAUSA300_2258	-	Formate dehydrogenase alpha chain (EC 1.2.1.2)	General function prediction only	9.83E-13	-5.08
SAUSA300_2260	-	Myo-inositol-1(or 4)-monophosphatase (EC 3.1.3.25)	Carbohydrate transport and metabolism	1.97E-11	-4.47
SAUSA300_2263	-	Transposase	Replication, recombination and repair	4.79E-13	24.30
SAUSA300_2264	-	Transcriptional regulator, RpiR family	Transcription	6.38E-10	-3.36
SAUSA300_2268	-	Transporter, Sodium/bile acid symporter family	General function prediction only	5.01E-06	3.61
SAUSA300_2270	glvC	PTS system, maltose and glucose-specific IIBC componen	Carbohydrate transport and metabolism	5.36E-03	3.01
SAUSA300_2273	-	Na+/H+ antiporter nhaC	Energy production and conversion	4.29E-04	3.39
SAUSA300_2277	hutI	Imidazolonepropionase (EC 3.5.2.7)	Secondary metabolites biosynthesis, transpo	2.48E-08	-16.15
SAUSA300_2280	fosB	Fosfomycin resistance protein	---	1.44E-04	7.13
SAUSA300_2281	hutG	Formiminoglutamase (EC 3.5.3.8)	Amino acid transport and metabolism	4.56E-09	-6.37
SAUSA300_2282	-	Hypothetical protein	General function prediction only	4.66E-07	-2.53
SAUSA300_2290	-	DNA-3-methyladenine glycosylase II (EC 3.2.2.21)	Replication, recombination and repair	1.73E-02	6.64
SAUSA300_2291	gltS	Sodium/glutamate symport carrier protein	Amino acid transport and metabolism	8.41E-10	6.76
SAUSA300_2298	-	Multidrug resistance protein B	---	4.63E-10	12.21
SAUSA300_2299	-	Multidrug resistance protein A	Defense mechanisms/Virulence	5.26E-11	37.02
SAUSA300_2304	-	EpiH/GdmH-related protein	Function unknown	3.80E-09	3.50
SAUSA300_2305	-	Transposase	---	8.03E-05	32.53
SAUSA300_2306	-	ABC transporter ATP-binding protein	Defense mechanisms/Virulence	3.85E-12	33.30
SAUSA300_2307	-	ABC transporter permease protein	Defense mechanisms/Virulence	2.91E-05	8.85
SAUSA300_2313	-	L-lactate permease	Energy production and conversion	4.04E-07	-2.79
SAUSA300_2316	-	Protease synthase and sporulation negative regulatory prot	General function prediction only	1.18E-09	3.43
SAUSA300_2321	-	Phage infection protein	Function unknown	1.16E-06	12.81
SAUSA300_2325	-	Hypothetical protein	---	5.11E-09	-2.14
SAUSA300_2327	-	General stress protein 26	---	2.07E-12	-4.27
SAUSA300_2331	-	Transcriptional regulator, MarR family	Transcription	1.22E-06	-3.92
SAUSA300_2333	narK	Nitrite extrusion protein	Inorganic ion transport and metabolism	4.90E-04	3.32
SAUSA300_2334	-	Hypothetical cytosolic protein	Posttranslational modification, protein turnov	1.23E-07	16.27
SAUSA300_2335	-	Hypothetical cytosolic protein	Posttranslational modification, protein turnov	3.86E-11	5.55
SAUSA300_2337	-	Two-component response regulator	Signal transduction mechanisms	6.95E-07	-8.94
SAUSA300_2339	-	Transcriptional regulator	Signal transduction mechanisms	1.36E-04	-6.74
SAUSA300_2340	narI	Respiratory nitrate reductase gamma chain (EC 1.7.99.4)	Energy production and conversion	2.91E-04	-3.28
SAUSA300_2341	narJ	Respiratory nitrate reductase delta chain (EC 1.7.99.4)	Energy production and conversion	6.54E-03	5.18
SAUSA300_2342	narH	Respiratory nitrate reductase beta chain (EC 1.7.99.4)	Energy production and conversion	2.05E-06	4.62
SAUSA300_2343	-	Respiratory nitrate reductase alpha chain (EC 1.7.99.4)	Energy production and conversion	8.02E-09	6.26
SAUSA300_2345	nirD	Nitrite reductase [NAD(P)H] small subunit (EC 1.7.1.4)	Inorganic ion transport and metabolism	1.62E-05	6.99
SAUSA300_2346	nirB	Nitrite reductase [NAD(P)H] large subunit (EC 1.7.1.4)	Energy production and conversion	1.66E-05	2.65
SAUSA300_2348	-	Acetyltransferase (EC 2.3.1.-)	General function prediction only	3.14E-08	2.92
SAUSA300_2350	-	Hypothetical cytosolic protein	Function unknown	1.86E-03	2.73
SAUSA300_2351	-	Hypothetical protein	General function prediction only	7.15E-04	5.19
SAUSA300_2356	fmhA	UDP-N-acetylmuramoylheptapeptide-glycine L-seryltrans	Defense mechanisms/Virulence	2.43E-09	6.82
SAUSA300_2361	-	Hypothetical protein	---	8.19E-06	15.57
SAUSA300_2364	sbi	IgG-binding protein SBI	---	2.57E-09	24.12
SAUSA300_2365	hlgA	Leukocidin S subunit	---	5.72E-08	20.80
SAUSA300_2366	hlgC	Leukocidin S subunit	---	2.42E-05	27.44
SAUSA300_2367	hlgB	Hypothetical protein	---	8.33E-12	76.31
SAUSA300_2368	-	BioX protein	---	1.27E-06	10.64
SAUSA300_2369	-	6-carboxyhexanoate--CoA ligase (EC 6.2.1.14)	Coenzyme transport and metabolism	1.21E-07	6.42
SAUSA300_2370	-	8-amino-7-oxononanoate synthase (EC 2.3.1.47)	Coenzyme transport and metabolism	2.10E-04	4.70
SAUSA300_2371	bioB	Biotin synthase (EC 2.8.1.6)	Coenzyme transport and metabolism	3.57E-09	5.11
SAUSA300_2372	bioA	Adenosylmethionine-8-amino-7-oxononanoate aminotrans	Coenzyme transport and metabolism	1.04E-11	107.14
SAUSA300_2373	bioD	Dethiobiotin synthetase (EC 6.3.3.3)	Coenzyme transport and metabolism	1.91E-09	45.91
SAUSA300_2374	-	ABC transporter ATP-binding and permease protein	Defense mechanisms/Virulence	2.41E-06	25.73
SAUSA300_2375	-	ABC transporter ATP-binding and permease protein	Defense mechanisms/Virulence	9.05E-06	25.58
SAUSA300_2380	-	Transcriptional activator aarP	Function unknown	7.14E-04	7.25
SAUSA300_2382	-	Phosphoesterase	General function prediction only	3.20E-03	2.05
SAUSA300_2385	-	Amino acid permease	Amino acid transport and metabolism	1.86E-05	3.76
SAUSA300_2387	-	dTDP-glucose 4,6-dehydratase (EC 4.2.1.46)	Cell wall/membrane biogenesis	1.25E-07	10.25
SAUSA300_2388	panE	2-dehydropantoate 2-reductase (EC 1.1.1.169)	Coenzyme transport and metabolism	7.05E-04	-3.11
SAUSA300_2390	opuCd	Glycine betaine transport system permease protein	Amino acid transport and metabolism	2.37E-03	2.09
SAUSA300_2393	opuCa	Glycine betaine transport ATP-binding protein	Amino acid transport and metabolism	2.76E-02	5.06
SAUSA300_2396	pnbA	Para-nitrobenzyl esterase (EC 3.1.1.-)	Lipid transport and metabolism	3.29E-11	-7.89

probe set mapping	synonym	description	NCBI COG	p-value	day one / inoculum
SAUSA300_2398	-	ABC transporter permease protein	General function prediction only	4.52E-11	-3.98
SAUSA300_2399	-	ABC transporter ATP-binding protein	General function prediction only	1.30E-08	-8.95
SAUSA300_2401	-	RelE protein	Function unknown	1.61E-02	7.48
SAUSA300_2404	-	Ferredoxin-dependent glutamate synthase (EC 1.4.7.1)	Amino acid transport and metabolism	2.71E-02	-5.56
SAUSA300_2406	-	Bicyclomycin resistance protein	---	1.41E-05	6.80
SAUSA300_2407	-	Nickel transport ATP-binding protein nikE	Amino acid transport and metabolism	7.44E-04	3.13
SAUSA300_2408	-	Nickel transport ATP-binding protein nikD	Amino acid transport and metabolism	6.46E-08	16.17
SAUSA300_2409	-	Nickel transport system permease protein nikC	Amino acid transport and metabolism	9.09E-16	21.25
SAUSA300_2410	-	Nickel transport system permease protein nikB	Amino acid transport and metabolism	1.66E-05	2.80
SAUSA300_2411	opp-1A	Nickel-binding protein	Amino acid transport and metabolism	2.66E-06	12.27
SAUSA300_2412	-	Hypothetical protein	Function unknown	4.68E-08	45.29
SAUSA300_2413	-	Methyltransferase (EC 2.1.1.-)	General function prediction only	1.46E-07	13.94
SAUSA300_2414	-	Diaminopimelate epimerase (EC 5.1.1.7)	Amino acid transport and metabolism	7.96E-08	23.53
SAUSA300_2415	-	Hypothetical protein	---	7.52E-12	-2.59
SAUSA300_2416	-	Hypothetical protein	Lipid transport and metabolism	6.38E-12	-15.71
SAUSA300_2418	-	Transposase	Function unknown	4.66E-07	-2.17
SAUSA300_2420	-	Hypothetical protein	---	4.07E-04	24.89
SAUSA300_2424	-	membrane lipoprotein	---	4.16E-10	25.11
SAUSA300_2425	-	Hypothetical protein	---	1.37E-04	3.31
SAUSA300_2430	-	membrane lipoprotein	---	1.65E-05	9.46
SAUSA300_2431	-	DNA/RNA helicase (DEAD/DEAH box family)	Transcription	1.20E-05	3.03
SAUSA300_2432	-	7,8-dihydro-8-oxoguanine-triphosphatase (EC 3.6.1.-)	Replication, recombination and repair	7.17E-05	2.48
SAUSA300_2434	-	Macrolide-efflux protein	---	2.70E-10	3.69
SAUSA300_2435	-	Beta-N-acetylhexosaminidase (EC 3.2.1.52)	Function unknown	1.80E-10	15.42
SAUSA300_2436	-	Beta-N-acetylhexosaminidase (EC 3.2.1.52)	---	9.23E-09	23.99
SAUSA300_2438	sarU	STAPHYLOCOCCAL ACCESSORY REGULATOR A	---	1.78E-05	3.69
SAUSA300_2440	fnbB	Hypothetical protein	---	1.66E-11	23.61
SAUSA300_2441	fnbA	Fibronectin-binding protein	---	1.66E-08	2.30
SAUSA300_2442	gntP	Gluconate permease	Carbohydrate transport and metabolism	6.79E-08	4.64
SAUSA300_2443	gntK	Gluconokinase (EC 2.7.1.12)	Carbohydrate transport and metabolism	8.81E-15	3.03
SAUSA300_2451	-	Bicyclomycin resistance protein	---	5.36E-04	8.07
SAUSA300_2452	-	Transcriptional regulator, MarR family	Transcription	1.10E-03	23.47
SAUSA300_2453	-	Lantibiotic transport ATP-binding protein	Defense mechanisms/Virulence	5.82E-10	27.77
SAUSA300_2454	-	ABC transporter ATP-binding protein	---	1.73E-05	13.92
SAUSA300_2456	-	Hypothetical membrane spanning protein	Function unknown	1.36E-02	4.67
SAUSA300_2458	-	Glyoxalase family protein	---	6.18E-07	25.55
SAUSA300_2460	-	Acetyltransferase (EC 2.3.1.-)	General function prediction only	2.47E-11	-3.40
SAUSA300_2461	-	Glyoxalase family protein	General function prediction only	2.82E-04	6.56
SAUSA300_2463	ddh	D-2-hydroxyacid dehydrogenase (EC 1.1.1.-)	Energy production and conversion	1.48E-13	-6.84
SAUSA300_2464	-	Hydrolase (HAD superfamily)	General function prediction only	1.39E-08	11.82
SAUSA300_2465	-	ABC transporter ATP-binding protein	Defense mechanisms/Virulence	2.42E-05	21.12
SAUSA300_2466	-	Hypothetical membrane spanning protein	---	2.19E-04	5.50
SAUSA300_2468	-	Phosphinothricin N-acetyltransferase (EC 2.3.1.-)	---	1.35E-02	2.90
SAUSA300_2475	-	Thioesterase (EC 3.1.2.-)	General function prediction only	7.97E-06	8.76
SAUSA300_2477	cidC	Pyruvate dehydrogenase [cytochrome] (EC 1.2.2.2)	Amino acid transport and metabolism	1.20E-12	-2.12
SAUSA300_2478	cidB	Murein hydrolase export regulator	Cell wall/membrane biogenesis	3.12E-12	-3.01
SAUSA300_2479	cidA	Murein hydrolase exporter	General function prediction only	7.12E-11	8.24
SAUSA300_2481	-	Hypothetical cytosolic protein	---	9.76E-13	-12.25
SAUSA300_2486	-	ATP-dependent endopeptidase clp ATP-binding subunit cl	Posttranslational modification, protein turnov	3.89E-05	-3.07
SAUSA300_2487	feoB	Ferrous iron transport protein B	Inorganic ion transport and metabolism	2.80E-07	5.33
SAUSA300_2489	-	Transporter, MMPL family	General function prediction only	1.50E-13	90.27
SAUSA300_2490	-	Transcriptional regulator, TetR family	Transcription	2.49E-03	-2.08
SAUSA300_2491	-	Delta-1-pyrroline-5-carboxylate dehydrogenase (EC 1.5.1	Energy production and conversion	1.37E-04	-3.37
SAUSA300_2492	-	O-acetyltransferase (cell wall biosynthesis) (EC 2.3.1.-)	General function prediction only	1.70E-07	12.39
SAUSA300_2498	crtN	Dehydrosqualene desaturase (EC 1.3.99.-)	Secondary metabolites biosynthesis, transpor	6.65E-11	-13.84
SAUSA300_2499	crtM	dehydrosqualene synthase (EC 2.5.1.-)	Lipid transport and metabolism	2.85E-12	-18.92
SAUSA300_2500	-	HPNB PROTEIN	Cell wall/membrane biogenesis	7.64E-12	-12.54
SAUSA300_2501	-	Phytoene desaturase (EC 1.14.99.-)	Secondary metabolites biosynthesis, transpor	1.20E-08	-2.77
SAUSA300_2502	-	Hypothetical protein	---	2.35E-11	-53.38
SAUSA300_2506	isaA	Hypothetical protein	---	3.05E-06	-2.92
SAUSA300_2507	-	Hypothetical membrane spanning protein	General function prediction only	2.45E-09	5.71
SAUSA300_2508	-	Hypothetical protein	---	1.34E-02	22.00
SAUSA300_2510	-	Hypothetical protein	---	2.78E-03	2.89
SAUSA300_2511	-	Hypothetical cytosolic protein	Function unknown	1.79E-04	14.79
SAUSA300_2512	-	Glyoxalase family protein	General function prediction only	8.23E-10	10.12
SAUSA300_2513	-	putative nucleoside-diphosphate-sugar epimerases	Cell wall/membrane biogenesis	6.81E-05	19.76
SAUSA300_2514	-	Hypothetical protein	Function unknown	3.73E-08	58.68
SAUSA300_2515	-	Transcriptional regulator, TetR family	Transcription	1.34E-05	5.27
SAUSA300_2516	-	Short chain dehydrogenase	Lipid transport and metabolism	1.45E-07	4.57
SAUSA300_2517	-	5-carboxyvanillic acid decarboxylase (EC 4.1.1.-)	General function prediction only	2.41E-06	-2.40
SAUSA300_2518	-	Esterase/Lipase (EC 3.1.-.-)	General function prediction only	2.04E-12	-7.24
SAUSA300_2519	-	Low-affinity zinc transport protein	General function prediction only	1.06E-13	3.65
SAUSA300_2520	-	Ferrous iron transport protein B	Inorganic ion transport and metabolism	2.91E-09	5.83
SAUSA300_2521	-	Oxidoreductase (EC 1.1.1.-)	Posttranslational modification, protein turnov	1.78E-08	41.36
SAUSA300_2524	-	Hypothetical protein	---	4.08E-04	-2.41
SAUSA300_2525	-	Fructosamine kinase family protein	Carbohydrate transport and metabolism	2.35E-10	-29.30
SAUSA300_2526	pyrD	Dihydroorotate dehydrogenase (EC 1.3.3.1)	Nucleotide transport and metabolism	4.82E-04	2.27
SAUSA300_2529	-	PhnB protein	Function unknown	2.80E-07	-2.24
SAUSA300_2530	-	Transcriptional regulator, TetR family	Transcription	5.40E-03	2.37
SAUSA300_2533	panC	Pantoate--beta-alanine ligase (EC 6.3.2.1)	Coenzyme transport and metabolism	2.03E-02	3.02

probe set mapping	synonym	description	NCBI COG	p-value	day one / inoculum
SAUSA300_2534	panB	3-methyl-2-oxobutanoate hydroxymethyltransferase (EC 2	Coenzyme transport and metabolism	8.64E-09	3.79
SAUSA300_2535	panE	2-dehydropantoate 2-reductase (EC 1.1.1.169)	Coenzyme transport and metabolism	1.19E-07	5.33
SAUSA300_2539	-	4-aminobutyrate aminotransferase (EC 2.6.1.19)	Amino acid transport and metabolism	5.21E-05	6.31
SAUSA300_2543	-	Signal transduction protein TRAP	General function prediction only	2.04E-05	-3.96
SAUSA300_2546	betB	Betaine aldehyde dehydrogenase (EC 1.2.1.8)	Energy production and conversion	3.94E-11	-7.73
SAUSA300_2549	bccT	Choline transport protein	Cell wall/membrane biogenesis	2.87E-06	-3.88
SAUSA300_2550	nrdG	Anaerobic ribonucleoside-triphosphate reductase activatin	Posttranslational modification, protein turnov	1.61E-03	-3.12
SAUSA300_2551	nrdD	Anaerobic ribonucleoside-triphosphate reductase (EC 1.17	Nucleotide transport and metabolism	8.33E-03	-2.53
SAUSA300_2552	-	Hypothetical protein	Energy production and conversion	2.04E-03	8.09
SAUSA300_2555	-	Glutathione peroxidase (EC 1.11.1.9)	Posttranslational modification, protein turnov	6.68E-04	7.55
SAUSA300_2558	-	Two-component sensor histidine kinase (EC 2.7.3.-)	Signal transduction mechanisms	1.41E-06	-2.06
SAUSA300_2559	-	Two-component response regulator	Signal transduction mechanisms	1.08E-05	-2.02
SAUSA300_2561	phoB	Alkaline phosphatase (EC 3.1.3.1)	Inorganic ion transport and metabolism	8.64E-09	17.23
SAUSA300_2563	-	Transcriptional regulator, MarR family	Transcription	9.49E-03	-2.46
SAUSA300_2571	argR	Arginine repressor, argR	Transcription	7.81E-11	34.55
SAUSA300_2573	isaB	Hypothetical protein	---	5.04E-07	-5.72
SAUSA300_2574	-	Hypothetical exported protein	---	1.16E-05	14.76
SAUSA300_2576	-	PTS system, mannose-specific IIAB component (EC 2.7.1	Carbohydrate transport and metabolism	1.23E-07	12.55
SAUSA300_2580	-	N-carbamoylsarcosine amidase (EC 3.5.1.59)	Secondary metabolites biosynthesis, transpor	3.19E-07	-2.25
SAUSA300_2582	-	surface protein Pls	---	2.83E-13	-23.83
SAUSA300_2583	-	Poly(Glycerol-phosphate) alpha-glucosyltransferase (EC 2	Cell wall/membrane biogenesis	3.56E-11	-2.48
SAUSA300_2584	-	Protein translocase subunit secA	Intracellular trafficking and secretion	3.39E-09	-2.36
SAUSA300_2585	-	Hypothetical cytosolic protein	---	4.18E-05	4.88
SAUSA300_2587	-	Hypothetical protein	---	1.22E-02	2.17
SAUSA300_2589	-	Cell surface protein	---	2.40E-02	-2.14
SAUSA300_2592	-	Hypothetical protein	---	2.35E-10	-30.33
SAUSA300_2594	msrA	Peptide methionine sulfoxide reductase (EC 1.8.4.11)	Posttranslational modification, protein turnov	2.08E-04	6.94
SAUSA300_2596	cap1C	Phosphotyrosine-protein phosphatase (capsular polysaccha	Carbohydrate transport and metabolism	1.56E-03	17.63
SAUSA300_2597	cap1B	Tyrosine-protein kinase (capsular polysaccharide biosynth	Cell cycle control, mitosis and meiosis	6.76E-07	9.09
SAUSA300_2598	cap1A	Chain length regulator (capsular polysaccharide biosynthe	Cell wall/membrane biogenesis	5.14E-07	124.10
SAUSA300_2599	tetR	Transcriptional regulator IcaR	Transcription	5.38E-04	-2.29
SAUSA300_2600	icaA	N-acetylglucosaminyltransferase (EC 2.4.1.-)	---	1.11E-11	95.95
SAUSA300_2601	icaB	Polysaccharide deacetylase	---	9.16E-10	15.78
SAUSA300_2603	lip	Lipase (EC 3.1.1.3)	General function prediction only	3.03E-12	-21.34
SAUSA300_2605	hisIE	Phosphoribosyl-AMP cyclohydrolase (EC 3.5.4.19) / Phos	Amino acid transport and metabolism	4.89E-05	8.03
SAUSA300_2606	hisF	Imidazole glycerol phosphate synthase, cyclase subunit (E	Amino acid transport and metabolism	1.45E-08	15.72
SAUSA300_2607	hisA	1-(5-phosphoribosyl)-5-[(5-phosphoribosylamino)methyli	Amino acid transport and metabolism	1.18E-05	6.75
SAUSA300_2608	hisH	Imidazole glycerol phosphate synthase, glutamine amidotr	Amino acid transport and metabolism	6.96E-06	8.01
SAUSA300_2609	hisB	Imidazoleglycerol-phosphate dehydratase (EC 4.2.1.19)	Amino acid transport and metabolism	1.53E-09	32.55
SAUSA300_2610	hisC	Histidinol-phosphate aminotransferase (EC 2.6.1.9)	Amino acid transport and metabolism	6.70E-12	61.05
SAUSA300_2611	hisD	Histidinol dehydrogenase (EC 1.1.1.23)	Amino acid transport and metabolism	1.76E-04	15.93
SAUSA300_2612	hisG	ATP phosphoribosyltransferase (EC 2.4.2.17)	Amino acid transport and metabolism	4.50E-05	8.51
SAUSA300_2613	hisZ	Hypothetical cytosolic protein	Amino acid transport and metabolism	6.81E-03	2.04
SAUSA300_2615	-	Ribosomal-protein-alanine acetyltransferase (EC 2.3.1.128	Translation	2.81E-02	5.79
SAUSA300_2618	-	Hypothetical protein	Function unknown	1.47E-08	13.91
SAUSA300_2619	-	Hypothetical cytosolic protein	Function unknown	4.62E-10	18.97
SAUSA300_2620	-	Hypothetical exported protein	Function unknown	3.84E-13	-4.06
SAUSA300_2622	-	Rhodanese-related sulfurtransferases	General function prediction only	6.99E-07	14.14
SAUSA300_2623	pcp	Pyrrolidone-carboxylate peptidase (EC 3.4.19.3)	Posttranslational modification, protein turnov	2.44E-12	10.56
SAUSA300_2624	-	Hypothetical protein	---	4.63E-06	2.32
SAUSA300_2625	-	Transcriptional regulator, PadR family	Transcription	3.34E-11	12.21
SAUSA300_2630	nixA	High-affinity nickel transport protein	Inorganic ion transport and metabolism	3.02E-08	-3.73
SAUSA300_2631	-	Hypothetical protein	Secondary metabolites biosynthesis, transpor	8.80E-05	6.10
SAUSA300_2632	-	Hypothetical membrane spanning protein	Function unknown	2.79E-06	-8.38
SAUSA300_2633	-	Bacitracin transport ATP-binding protein ytsC	Defense mechanisms/Virulence	7.46E-07	41.80
SAUSA300_2634	-	ABC transporter permease protein	Secondary metabolites biosynthesis, transpor	2.06E-05	66.60
SAUSA300_2637	-	Hypothetical protein	---	7.20E-04	2.42
SAUSA300_2639	-	Cold shock protein	Transcription	7.96E-08	-14.25
SAUSA300_2648	rpmH	LSU ribosomal protein L34P	---	4.29E-09	-2.30
SAUSA300_pUSA020001	-	TETRACYCLINE RESISTANCE PROTEIN TETA(L)/TI	---	1.43E-13	204.47
SAUSA300_pUSA020002	-	Plasmid recombination protein, Mob family	---	1.17E-11	118.17
SAUSA300_pUSA020003	-	Replication initiation protein, RepA family	Replication, recombination and repair	1.13E-11	60.50
SAUSA300_pUSA030001	repA	Replication initiator protein	---	2.02E-06	511.47
SAUSA300_pUSA030003	ileS	Isoleucyl-tRNA synthetase, mupirocin resistant (EC 6.1.1.	Translation	5.71E-12	1392.85
SAUSA300_pUSA030006	-	Replication and maintenance protein	---	3.70E-11	1560.01
SAUSA300_pUSA030007	ermC	rRNA adenine N-6-methyltransferase (EC 2.1.1.48)	---	7.35E-07	98.15
SAUSA300_pUSA030009	-	Hypothetical protein	---	3.82E-04	44.93
SAUSA300_pUSA030010	traA	Hypothetical protein	---	3.07E-13	468.31
SAUSA300_pUSA030011	traB	Hypothetical protein	---	3.05E-14	92.86
SAUSA300_pUSA030013	traD	Hypothetical protein	---	1.64E-10	592.59
SAUSA300_pUSA030014	traE	TRSE PROTEIN	Intracellular trafficking and secretion	2.10E-11	37.45
SAUSA300_pUSA030015	traF	Hypothetical protein	---	2.52E-08	157.15
SAUSA300_pUSA030016	traG	Secretory antigen precursor SsaA	---	1.57E-14	373.31
SAUSA300_pUSA030017	traH	Hypothetical protein	---	2.41E-06	214.40
SAUSA300_pUSA030018	traI	DNA topoisomerase III (EC 5.99.1.2)	Replication, recombination and repair	2.55E-15	300.34
SAUSA300_pUSA030019	traJ	Hypothetical protein	---	3.13E-08	194.77
SAUSA300_pUSA030020	traK	TraG/TraD family	Intracellular trafficking and secretion	1.26E-11	71.06
SAUSA300_pUSA030021	traL	Hypothetical protein	---	1.71E-09	183.92
SAUSA300_pUSA030022	traM	Hypothetical protein	Replication, recombination and repair	1.73E-05	165.23
SAUSA300_pUSA030023	traO	Hypothetical protein	---	2.10E-05	965.74

probe set mapping	synonym	description	NCBI COG	p-value	day one / inoculum
SAUSA300_pUSA030024	-	Transposase	Replication, recombination and repair	1.98E-11	1034.30
SAUSA300_pUSA030025	-	Hypothetical protein	---	4.84E-10	358.06
SAUSA300_pUSA030026	-	Hypothetical protein	Function unknown	1.83E-06	49.90
SAUSA300_pUSA030027	-	Hypothetical protein	---	3.53E-14	62.75
SAUSA300_pUSA030028	-	DNA integration/recombination/inversion protein	Replication, recombination and repair	1.85E-08	18.18
SAUSA300_pUSA030029	-	Hypothetical protein	---	4.45E-11	215.73
SAUSA300_pUSA030030	-	Hypothetical protein	---	3.77E-09	81.47
SAUSA300_pUSA030031	-	LtrC	---	1.45E-08	221.20
SAUSA300_pUSA030032	nes	Conjugal transfer protein traA	Replication, recombination and repair	9.96E-10	922.68
SAUSA300_pUSA030034	-	Hypothetical protein	---	6.95E-11	277.50
SAUSA300_pUSA030035	-	Hypothetical protein	---	5.01E-13	464.23
SAUSA300_pUSA030036	-	Hypothetical protein	---	2.27E-12	1730.34
SAUSA3000050-0052.m_at	-	psuedogene	---	3.29E-07	28.44
SAUSA3000086-0088.rc_at	-	psuedogene	---	1.14E-04	3.06
SAUSA3000148-0151.m_at	-	psuedogene	---	9.08E-03	3.14
SAUSA3000154-0156.m_at	-	psuedogene	---	1.75E-10	57.40
SAUSA3000154-0156.rc_at	-	psuedogene	---	1.97E-10	28.73
SAUSA3000411-0413.m_at	-	psuedogene	---	7.19E-06	7.12
SAUSA3000411-0413.rc_at	-	psuedogene	---	8.79E-04	2.51
SAUSA3000737-0739.rc_at	-	psuedogene	---	4.99E-04	2.16
SAUSA3001438-1440.m_at	-	psuedogene	---	6.67E-05	2.10
SAUSA3001708-1710.m_at	-	psuedogene	---	8.70E-07	36.52
SAUSA3001708-1710.rc_at	-	psuedogene	---	1.04E-03	11.71
SAUSA3001726-1728.m_at	-	psuedogene	---	5.44E-09	33.07
SAUSA3001726-1728.rc_at	-	psuedogene	---	8.31E-09	41.49
SAUSA3001747-1749.m_at	-	psuedogene	---	6.06E-04	2.87
SAUSA3001747-1749.rc_at	-	psuedogene	---	7.98E-05	4.41
SAUSA3001916-1918.m_at	-	psuedogene	---	1.16E-04	3.40
SAUSA3001916-1918.rc_at	-	psuedogene	---	7.24E-07	30.13
SAUSA3002169-2171.m_at	-	psuedogene	---	2.81E-12	17.90
SAUSA3002169-2171.rc_at	-	psuedogene	---	2.28E-13	8.95
SAUSA3002426-2428.m_at	-	psuedogene	---	5.21E-11	23.65
SAUSA3002426-2428.rc_at	-	psuedogene	---	6.35E-07	12.06
