# Supplementary material for: Insights into the Staphylococcus aureus-Host Interface: Global Changes in Host and Pathogen Gene Expression in a Rabbit Skin Infection Model
Source: PLoS One. 2015 Feb 26;10(2):e0117713. doi: 10.1371/journal.pone.0117713 (PMC4342162; doi:10.1371/journal.pone.0117713)
Supplement: S2 Table — Gene expression data was obtained using RT2 Profiler PCR Array Rabbit Inflammatory Cytokines and Receptors platform (QIAGEN). Cytokine transcripts that were changed significantly at any time point are in bold font. (DOCX) [file pone.0117713.s004.docx]

**Table S2.**

| Gene | Day 1 | | Day 3 | | Day 6 | | Day 10 | | Day 14 | |
| --- | --- | --- | --- | --- | --- | --- | --- | --- | --- | --- |
|  | **Fold Up- or Down-Regulation** | **p value** | **Fold Up- or Down-Regulation** | **p value** | **Fold Up- or Down-Regulation** | **p value** | **Fold Up- or Down-Regulation** | **p value** | **Fold Up- or Down-Regulation** | **p value** |
| *AIMP1* (SCYE-1) | -1.39 | 0.2218 | -1.19 | 0.4522 | -1.15 | 0.4885 | -1.36 | 0.3169 | -1.23 | 0.4843 |
| *BMP2* | -1.77 | 0.2159 | -1.45 | 0.3077 | -1.10 | 0.5804 | -5.78 | 0.0679 | -4.12 | 0.1927 |
| *BMP4* | **-5.95** | **0.0011** | **-7.27** | **0.0004** | **-14.46** | **0.0003** | **-12.05** | **0.0004** | **-5.14** | **0.0077** |
| *BMP7* | **-57.51** | **0.0177** | **-39.50** | **0.0187** | **-60.64** | **0.0178** | **-24.86** | **0.0195** | -14.31 | 0.0718 |
| *CCL2* (MCP-1) | **28.51** | **0.0007** | **5.57** | **0.0011** | 2.15 | 0.1109 | 2.44 | 0.0636 | -1.49 | 0.5348 |
| *CCL4* (MIP-1β; ACT2) | **513.86** | **0.0000** | **508.06** | **0.0003** | 804.95 | 0.0798 | 392.39 | 0.0939 | **93.79** | **0.0072** |
| *CCR1* | **588.19** | **0.0196** | **286.30** | **0.0037** | **244.59** | **0.0040** | **302.46** | **0.0348** | **102.06** | **0.0042** |
| *CCR3* | **220.32** | **0.0163** | **146.44** | **0.0005** | 25.35 | 0.0760 | **70.04** | **0.0368** | **61.14** | **0.0379** |
| *CCR5* | **12.51** | **0.0068** | **19.44** | **0.0063** | **11.90** | **0.0080** | **12.40** | **0.0203** | **6.68** | **0.0238** |
| *CNTF* | -1.90 | 0.0277 | -1.57 | 0.0591 | **-2.61** | **0.0092** | **-2.49** | **0.0132** | -1.87 | 0.0735 |
| *CX3CR1* | **-7.12** | **0.0092** | 2.34 | 0.1212 | **3.26** | **0.0005** | 2.70 | 0.0759 | **5.99** | **0.0145** |
| *CXCL13* | **111.02** | **0.0059** | 326.59 | 0.0509 | 528.84 | 0.2366 | 1278.65 | 0.1897 | 2125.96 | 0.1188 |
| *CXCR1* | **624.68** | **0.0096** | **315.41** | **0.0087** | **352.88** | **0.0006** | **210.19** | **0.0390** | **58.47** | **0.0001** |
| *CXCR2* | **595.98** | **0.0084** | **246.64** | **0.0135** | **243.31** | **0.0005** | 174.60 | 0.0560 | **57.13** | **0.0145** |
| *CXCR4* | 5.16 | 0.0629 | **7.19** | **0.0417** | **6.22** | **0.0025** | **4.66** | **0.0485** | 3.97 | 0.0575 |
| *CXCR5* | 1.32 | 0.3252 | 1.98 | 0.0268 | **3.11** | **0.0012** | 3.74 | 0.1949 | **7.75** | **0.0108** |
| *CSF2* (GMCSF) | -1.22 | 0.4953 | 1.32 | 0.5556 | 1.57 | 0.4136 | 2.33 | 0.3763 | 1.22 | 0.7808 |
| *IFNG* | -1.54 | 0.2120 | -1.16 | 0.5328 | 1.28 | 0.4890 | -1.02 | 0.8978 | 1.32 | 0.5317 |
| *IL10* | **3.97** | **0.0404** | **2.44** | **0.0011** | 1.87 | 0.0475 | 1.49 | 0.2456 | 1.89 | 0.2368 |
| *IL15* | 1.47 | 0.4012 | 1.22 | 0.7132 | 1.18 | 0.8651 | -1.11 | 0.6716 | -1.58 | 0.3956 |
| *IL16* | -1.06 | 0.8432 | -1.02 | 0.8338 | -1.46 | 0.0844 | -1.16 | 0.7022 | 1.26 | 0.1777 |
| *IL17RA* | **16.42** | **0.0044** | **11.66** | **0.0102** | **8.68** | **0.0053** | 14.55 | 0.0669 | **7.40** | **0.0000** |
| *IL18* | -1.02 | 0.8237 | -1.08 | 0.6634 | -1.37 | 0.1877 | 1.09 | 0.7116 | 1.22 | 0.4863 |
| *IL18R1* | **7.52** | **0.0052** | **6.03** | **0.0010** | **6.31** | **0.0161** | 5.70 | 0.1039 | **4.04** | **0.0019** |
| *IL1A* | **36.81** | **0.0132** | **69.69** | **0.0059** | **92.54** | **0.0214** | 49.57 | 0.2073 | **33.95** | **0.0413** |
| *IL1B* | **1559.49** | **0.0060** | **1113.92** | **0.0019** | **1316.37** | **0.0043** | 800.13 | 0.0829 | **150.08** | **0.0017** |
| *IL1R1* | **52.99** | **0.0003** | **36.95** | **0.0002** | **69.05** | **0.0017** | 54.59 | 0.1063 | **19.15** | **0.0002** |
| *IL22RA1* | 1.23 | 0.5879 | 1.78 | 0.9910 | 2.15 | 0.8013 | -13.30 | 0.1670 | -9.66 | 0.2899 |
| *IL4* | 3.22 | 0.0863 | 1.24 | 0.9560 | -1.95 | 0.3411 | -3.34 | 0.2494 | -3.01 | 0.3374 |
| *IL4R* | **7.07** | **0.0088** | **21.15** | **0.0002** | **25.91** | **0.0007** | 26.23 | 0.0961 | **45.59** | **0.0050** |
| *IL6* | **222.12** | **0.0030** | **226.92** | **0.0353** | 87.41 | 0.2110 | 47.29 | 0.2432 | 8.96 | 0.0598 |
| *IL6ST* (GP130) | -1.28 | 0.3196 | 1.20 | 0.5268 | 1.23 | 0.4477 | 1.07 | 0.8959 | 1.30 | 0.4270 |
| *IL7R* | 1.76 | 0.4433 | 6.86 | 0.0172 | 18.56 | 0.0008 | 13.05 | 0.0001 | 16.86 | 0.0005 |
| *IL8* | **8195.87** | **0.0004** | **5111.54** | **0.0082** | **11175.46** | **0.0368** | 3923.63 | 0.0949 | **834.02** | **0.0000** |
| *ADIPOQ* (LOC100009027) | **-16.85** | **0.0213** | **-4.04** | **0.0429** | **-4.70** | **0.0392** | **-5.28** | **0.0439** | -2.37 | 0.1675 |
| *LTB* (LOC100144336) | **10.65** | **0.0101** | **8.95** | **0.0060** | **12.15** | **0.0081** | **23.82** | **0.0112** | **19.47** | **0.0002** |
| *IFNAR2* (LOC100338097) | 6.24 | 0.3558 | **99.85** | **0.0005** | **133.97** | **0.0094** | **198.20** | **0.0008** | **332.95** | **0.0023** |
| *CCR7* (LOC100338432) | **3.52** | **0.0293** | **2.91** | **0.0465** | **4.38** | **0.0177** | 4.63 | 0.1007 | **6.77** | **0.0303** |
| CSF1 (LOC100338577; MCSF) | **3.65** | **0.0102** | **2.45** | **0.0015** | 1.62 | 0.0859 | 1.34 | 0.4079 | -1.29 | 0.3037 |
| *MIF* (LOC100338701) | **2.99** | **0.0389** | **3.63** | **0.0012** | **3.89** | **0.0058** | **3.97** | **0.0126** | **3.20** | **0.0075** |
| *IL17A* (LOC100339322) | 3.49 | 0.2115 | 23.60 | 0.0871 | 46.87 | 0.2233 | **19.87** | **0.0379** | **26.52** | **0.0260** |
| *CCR6* (LOC100339537) | -1.96 | 0.3036 | 2.37 | 0.2188 | 2.56 | 0.1588 | 3.65 | 0.0995 | **10.54** | **0.0432** |
| *IL17F* (LOC100339570) | **39.05** | **0.0014** | **170.94** | **0.0286** | 112.41 | 0.1431 | **80.59** | **0.0002** | **117.91** | **0.0115** |
| *IL9* (LOC100340820) | 2.13 | 0.9471 | 2.71 | 0.7256 | 3.68 | 0.3552 | -13.45 | 0.2077 | -3.53 | 0.3718 |
| *TNFSF11* (LOC100341009) | -2.13 | 0.3841 | -1.13 | 0.5206 | 1.94 | 0.8596 | 1.29 | 0.7447 | 2.42 | 0.7502 |
| *OSM* (LOC100342491) | **798.98** | **0.0028** | **586.15** | **0.0013** | **725.86** | **0.0095** | 744.29 | 0.0617 | **154.45** | **0.0176** |
| *NAMPT* (LOC100343645) | **2.61** | **0.0033** | **2.06** | **0.0252** | **1.61** | **0.0397** | **1.30** | 0.2719 | **-1.56** | **0.0471** |
| *IL21* (LOC100344172) | 2.60 | 0.1322 | **11.91** | **0.0059** | 9.60 | 0.2268 | 8.17 | 0.2171 | **15.75** | **0.0007** |
| *IL7* (LOC100345409) | -1.80 | 0.1422 | -1.28 | 0.3804 | -1.62 | 0.2302 | -1.71 | 0.3168 | 1.12 | 0.8789 |
| *CCR8* (LOC100345500) | 1.19 | 0.5288 | 2.43 | 0.9254 | 3.81 | 0.4108 | -3.80 | 0.2873 | -1.79 | 0.4730 |
| *IL25* (LOC100345869) | -2.08 | 0.2479 | 1.07 | 0.7995 | 1.05 | 0.7642 | -11.27 | 0.0820 | -6.36 | 0.2194 |
| *CX3CL1* (LOC100346186) | -10.10 | 0.0531 | **-21.76** | **0.0462** | **-17.57** | **0.0494** | -18.46 | 0.0511 | -8.61 | 0.1375 |
| *CCR4* (LOC100347328) | **4.59** | **0.0054** | **8.91** | **0.0139** | 3.66 | 0.1928 | 4.43 | 0.1211 | **10.58** | **0.0012** |
| *IL5RA* (LOC100347606) | 2.46 | 0.1419 | **4.44** | **0.0072** | 2.20 | 0.1308 | 3.35 | 0.1122 | **4.27** | **0.0118** |
| *IL6R* (LOC100347752) | **13.01** | **0.0017** | **10.67** | **0.0308** | **11.13** | **0.0012** | **15.92** | **0.0217** | **6.54** | **0.0134** |
| *TNFSF14* (LOC100347876) | 4.28 | 0.1738 | 3.32 | 0.3002 | 5.39 | 0.0526 | 4.14 | 0.2153 | 4.45 | 0.2559 |
| *CCL19* (LOC100348362) | **4.45** | **0.0184** | **4.03** | **0.0299** | -1.82 | 0.5541 | -11.87 | 0.1049 | -1.97 | 0.3077 |
| *CCL3* (LOC100348776) | **657.69** | **0.0001** | **598.81** | **0.0056** | 1230.36 | 0.0948 | 514.89 | 0.0867 | 153.27 | 0.0817 |
| *IL22* (LOC100348980) | **20.98** | **0.0207** | **113.36** | **0.0127** | **49.63** | **0.0357** | **25.46** | **0.0219** | **25.16** | **0.0037** |
| *IL12A* (LOC100349007) | 1.54 | 0.8569 | 2.58 | 0.6461 | 2.50 | 0.6657 | -2.78 | 0.2362 | -1.39 | 0.4629 |
| *IL27* (LOC100349321) | 3.09 | 0.4985 | 2.71 | 0.6737 | 3.05 | 0.5166 | -2.23 | 0.2699 | -1.39 | 0.4554 |
| *CXCR3* (LOC100349863) | -5.12 | 0.0635 | 1.47 | 0.4190 | 2.72 | 0.1713 | 5.63 | 0.2978 | **16.14** | **0.0100** |
| *LEPR* (LOC100351924) | -2.26 | 0.3406 | -1.21 | 0.6218 | -1.57 | 0.4377 | -21.40 | 0.2754 | -17.14 | 0.3512 |
| *IL23A* (LOC100352065) | **18.46** | **0.0225** | **21.55** | **0.0008** | **27.05** | **0.0072** | 14.13 | 0.1593 | **7.03** | **0.0224** |
| *IL20* (LOC100353705) | -1.50 | 0.3320 | 1.41 | 0.9388 | -2.19 | 0.6962 | 1.62 | 0.4042 | 6.67 | 0.2801 |
| LOC100354804 *(CXCL2)* | **4.67** | **0.0366** | **9.06** | **0.0030** | **14.27** | **0.0207** | 4.38 | 0.2588 | 2.75 | 0.1305 |
| *IL10RA* (LOC100354902) | **10.83** | **0.0043** | **13.23** | **0.0082** | **8.61** | **0.0001** | **8.45** | **0.0186** | **6.85** | **0.0010** |
| *TNFSF10* (LOC100355808; TRAIL) | 1.54 | 0.4416 | **4.19** | **0.0171** | 2.36 | 0.0983 | 1.50 | 0.5069 | 1.20 | 0.9067 |
| *IL33* (LOC100356081) | **5.13** | **0.0001** | **4.66** | **0.0004** | -1.01 | 0.7881 | 1.03 | 0.9340 | 1.02 | 0.9308 |
| *CSF3* (LOC100356652; GCSF) | **10.94** | **0.0150** | **5.72** | **0.0322** | 4.42 | 0.0514 | 1.23 | 0.9223 | 1.71 | 0.8618 |
| *IL5* (LOC100358075) | -4.19 | 0.1006 | -6.36 | 0.0788 | -6.68 | 0.0766 | -5.69 | 0.0850 | -3.76 | 0.2035 |
| *CCR2* (LOC100358097) | **8.20** | **0.0038** | **10.24** | **0.0104** | **7.03** | **0.0032** | **6.50** | **0.0229** | **4.01** | **0.0261** |
| *CD40LG* (LOC100358388) | **4.21** | **0.0307** | **3.80** | **0.0076** | 5.15 | 0.1131 | 5.58 | 0.1358 | **9.01** | **0.0048** |
| *IL13* (LOC100358676) | -1.25 | 0.4986 | 1.53 | 0.6420 | -1.10 | 0.6815 | -2.80 | 0.1446 | -13.09 | 0.1853 |
| *LIF* (LOC100358914) | **10.94** | **0.0058** | **18.39** | **0.0000** | **9.55** | **0.0046** | 9.20 | 0.0662 | **3.66** | **0.0294** |
| *LTA* (TNFβ) | 3.04 | 0.8536 | 7.62 | 0.1059 | 12.56 | 0.2531 | 32.67 | 0.2005 | **89.57** | **0.0023** |
| *SPP1* (OPN) | 5.05 | 0.0531 | 25.34 | 0.2470 | 28.34 | 0.1123 | **13.20** | **0.0342** | 11.52 | 0.0564 |
| *TGFB2* | **-3.47** | **0.0176** | **-2.26** | **0.0383** | **-3.01** | **0.0333** | **-2.50** | **0.0373** | -1.76 | 0.1626 |
| *TNF* | **18.57** | **0.0018** | **40.26** | **0.0103** | **40.61** | **0.0275** | 21.12 | 0.0571 | **12.71** | **0.0001** |
| *TNFRSF11B* | 2.77 | 0.1794 | **3.00** | **0.0304** | -1.14 | 0.6146 | -1.31 | 0.4763 | -1.29 | 0.5693 |
| *TNFSF13* | -1.55 | 0.4008 | 1.27 | 0.9908 | 1.19 | 0.9915 | 2.13 | 0.2982 | 3.12 | 0.1241 |
| *TNFSF13B* | **6.67** | **0.0063** | **23.21** | **0.0037** | **22.23** | **0.0006** | **14.13** | **0.0426** | **13.01** | **0.0075** |
| *TNFSF4* (OX40L) | **4.56** | **0.0051** | **5.27** | **0.0001** | **6.32** | **0.0013** | **3.93** | **0.0008** | **3.44** | **0.0069** |
| *VEGFA* | 1.34 | 0.2558 | 2.70 | 0.0091 | 1.72 | 0.2119 | 1.74 | 0.2736 | -1.03 | 0.8353 |
| *ACTA2* | -1.88 | 0.2341 | -1.68 | 0.0016 | -1.30 | 0.0028 | -1.42 | 0.2423 | 1.34 | 0.1549 |
| *ACTB* | **2.85** | **0.0359** | **2.67** | **0.0192** | **2.84** | **0.0111** | **3.27** | **0.0057** | **2.76** | **0.0428** |
| *GAPDH* | -1.02 | 0.7175 | -1.58 | 0.2726 | -2.17 | 0.1615 | -2.41 | 0.1527 | -3.00 | 0.2153 |
| *LDHA* | -1.10 | 0.6745 | 1.12 | 0.8576 | 1.02 | 0.8793 | -1.15 | 0.7228 | -1.88 | 0.2104 |
| LOC100346936 | -1.35 | 0.3300 | -1.13 | 0.5775 | -1.02 | 0.7659 | 1.19 | 0.6985 | 1.53 | 0.3295 |
