# Supplementary material for: Insights into the Staphylococcus aureus-Host Interface: Global Changes in Host and Pathogen Gene Expression in a Rabbit Skin Infection Model
Source: PLoS One. 2015 Feb 26;10(2):e0117713. doi: 10.1371/journal.pone.0117713 (PMC4342162; doi:10.1371/journal.pone.0117713)
Supplement: S3 Table — Human and rabbit blood results are expressed as an average relative fold change in rabbit or human transcripts expressed by cells in the whole blood co-cultured with 1 x 10^7^ CFU/ml of S. aureus compared to the level of transcripts expression in heparinized blood alone. Gene expression data was obtained using RT2 Profiler PCR Array Rabbit or Human Inflammatory Cytokines and Receptors platform (QIAGEN). Cytokines, which transcript levels changed above 2-fold and were statistically significant are highlighted in bold. (DOCX) [file pone.0117713.s005.docx]

**Table S3.**

| Gene Symbol | Rabbit blood | | Human blood | |
| --- | --- | --- | --- | --- |
|  | **Fold Up- or Down-regulation** | ***p* value** | **Fold Up- or Down-regulation** | ***p* value** |
| *ACTB* | 1.04 | 0.3579 | 1.08 | 0.5790 |
| *AIMP1* | 1.18 | 0.1817 | 1.12 | 0.4572 |
| *CCL2* | 6.83 | 0.0700 | 2.66 | 0.2568 |
| *CCL3* (LOC100348776) | **32.61** | **0.0435** | 7.49 | 0.1023 |
| *CCL4* | **9.98** | **0.0100** | 2.46 | 0.1372 |
| *CCR1* | 1.27 | 0.0489 | 1.39 | 0.3219 |
| *CCR2* (LOC100358097) | -2.42 | 0.0843 | **-4.40** | **0.0499** |
| *CCR3* | -1.17 | 0.6620 | -3.00 | 0.0903 |
| *CCR4* (LOC100347328) | 1.26 | 0.1316 | 1.91 | 0.1410 |
| *CCR5* | -1.68 | 0.1422 | 1.00 | 0.9090 |
| *CCR6* (LOC100339537) | **2.89** | **0.0342** | 1.88 | 0.2674 |
| *CCR8* (LOC100345500) | 1.56 | 0.6720 | 2.74 | 0.1893 |
| *CD40LG* (LOC100358388) | 1.34 | 0.0703 | 1.09 | 0.6892 |
| *CX3CL1* (LOC100346186*)* | -2.78 | 0.2455 | 1.16 | 0.9989 |
| *CX3CR1* | **-4.95** | **0.0063** | -1.93 | 0.3904 |
| *CXCL13* | -1.12 | 0.5725 | -2.87 | 0.2491 |
| *CXCL2* (LOC100354804) | **33.14** | **0.0007** | 9.57 | 0.0637 |
| *CXCR1* | **-2.62** | **0.0237** | -3.96 | 0.1014 |
| *CXCR2* | **-2.27** | **0.0088** | -3.28 | 0.1472 |
| *GAPDH* | -1.02 | 0.9739 | -1.18 | 0.1302 |
| *IFNG* | 1.23 | 0.8174 | **8.57** | **0.0485** |
| *IL10RA* (LOC100354902) | 1.16 | 0.2371 | 1.19 | 0.2567 |
| *IL13* (LOC100358676) | **4.29** | **0.0042** | **9.92** | **0.0123** |
| *IL15* | -1.94 | 0.2634 | -1.28 | 0.1679 |
| *IL16* | -1.22 | 0.2331 | 1.34 | 0.3600 |
| *IL17A* (LOC100339322) | 17.67 | 0.2282 | 4.80 | 0.3617 |
| *IL17F* (LOC100339570) | 6.28 | 0.1405 | 5.58 | 0.1384 |
| *IL1A* | **59.47** | **0.0179** | **7.73** | **0.0396** |
| *IL1B* | **2.23** | **0.0084** | 2.44 | 0.1896 |
| *IL1R1* | **4.76** | **0.0009** | **7.01** | **0.0477** |
| *IL21* (LOC100344172) | 1.68 | 0.0811 | **5.01** | **0.0038** |
| *IL27* (LOC100349321) | 2.00 | 0.0736 | -1.13 | 0.7683 |
| *IL33* (LOC100356081) | **20.60** | **0.0060** | 1.56 | 0.1903 |
| *IL5* (LOC100358075) | -1.09 | 0.7603 | 1.04 | 0.8555 |
| *IL5RA* (LOC100347606) | 1.53 | 0.1125 | -2.97 | 0.1075 |
| *IL7* (LOC100345409) | 1.04 | 0.9330 | **4.41** | **0.0140** |
| *IL8* | **3.37** | **0.0011** | 9.95 | 0.0625 |
| *IL9* (LOC100340820) | 3.90 | 0.4726 | 5.75 | 0.3663 |
| *LTA* | 1.81 | 0.2335 | **3.28** | **0.0102** |
| *LTB* (LOC100144336) | 1.16 | 0.2991 | 1.32 | 0.2980 |
| *MIF* (LOC100338701) | 1.08 | 0.6945 | 1.17 | 0.3922 |
| *NAMPT* (LOC100343645) | 1.45 | 0.1807 | 1.43 | 0.4369 |
| *OSM (LOC100342491)* | **4.05** | **0.0117** | 16.28 | 0.1163 |
| *SPP1* | 1.23 | 0.3936 | 4.64 | 0.1535 |
| *TNF* | **7.93** | **0.0079** | **6.47** | **0.0263** |
| *TNFRSF11B* | 1.62 | **0.0217** | **42.99** | **0.0168** |
| *TNFSF11* (LOC100341009) | 1.68 | 0.0574 | 2.57 | 0.0824 |
| *TNFSF13* | **-2.24** | **0.0026** | -1.04 | 0.7725 |
| *TNFSF13B* | -1.82 | 0.0792 | **-3.35** | **0.0341** |
| *TNFSF4* | -1.37 | 0.2989 | -1.08 | 0.8017 |
| *VEGFA* | 1.99 | 0.0839 | 20.90 | 0.0640 |
